# Sources of Signal in 62 Protein-Coding Nuclear Genes for Higher-Level Phylogenetics of Arthropods

**DOI:** 10.1371/journal.pone.0023408

**Published:** 2011-08-04

**Authors:** Jerome C. Regier, Andreas Zwick

**Affiliations:** 1 Institute for Bioscience and Biotechnology Research, University of Maryland, College Park, Maryland, United States of America; 2 Department of Entomology, University of Maryland, College Park, Maryland, United States of America; 3 Center for Biosystems Research, University of Maryland Biotechnology Institute, College Park, Maryland, United States of America; 4 Entomology, State Museum of Natural History, Stuttgart, Germany; American Museum of Natural History, United States of America

## Abstract

**Background:**

This study aims to investigate the strength of various sources of phylogenetic information that led to recent seemingly robust conclusions about higher-level arthropod phylogeny and to assess the role of excluding or downweighting synonymous change for arriving at those conclusions.

**Methodology/Principal Findings:**

The current study analyzes DNA sequences from 68 gene segments of 62 distinct protein-coding nuclear genes for 80 species. Gene segments analyzed individually support numerous nodes recovered in combined-gene analyses, but few of the higher-level nodes of greatest current interest. However, neither is there support for conflicting alternatives to these higher-level nodes. Gene segments with higher rates of nonsynonymous change tend to be more informative overall, but those with lower rates tend to provide stronger support for deeper nodes. Higher-level nodes with bootstrap values in the 80% – 99% range for the complete data matrix are markedly more sensitive to substantial drops in their bootstrap percentages after character subsampling than those with 100% bootstrap, suggesting that these nodes are likely not to have been strongly supported with many fewer data than in the full matrix. Data set partitioning of total data by (mostly) synonymous and (mostly) nonsynonymous change improves overall node support, but the result remains much inferior to analysis of (unpartitioned) nonsynonymous change alone. Clusters of genes with similar nonsynonymous rate properties (e.g., faster vs. slower) show some distinct patterns of node support but few conflicts. Synonymous change is shown to contribute little, if any, phylogenetic signal to the support of higher-level nodes, but it does contribute nonphylogenetic signal, probably through its underlying heterogeneous nucleotide composition. Analysis of seemingly conservative indels does not prove useful.

**Conclusions:**

Generating a robust molecular higher-level phylogeny of Arthropoda is currently possible with large amounts of data and an exclusive reliance on nonsynonymous change.

## Introduction

**Figure 1:**
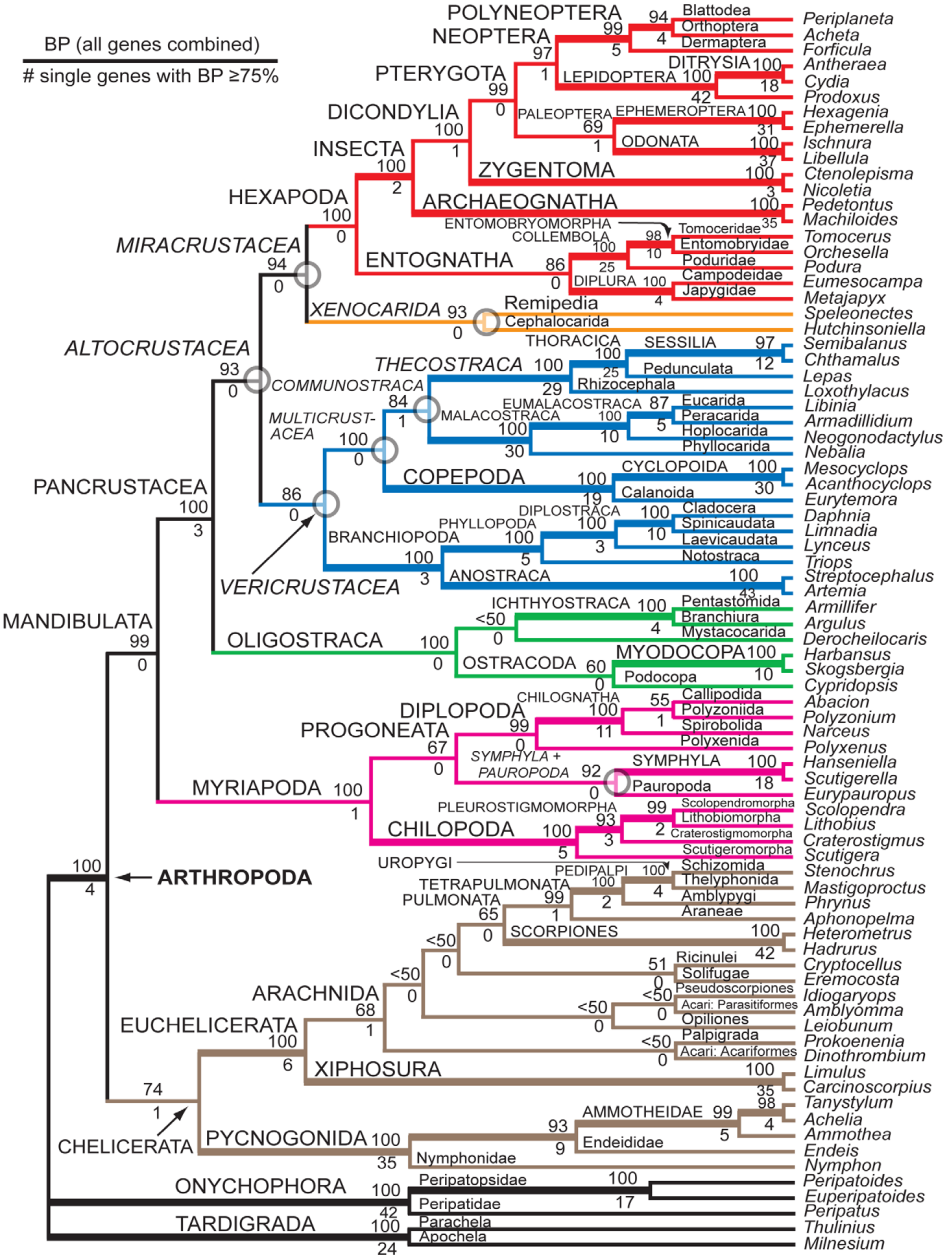
Higher-level arthropod relationships based on likelihood analysis of aligned, concatenated *nt123_degen1* sequences. The maximum-likelihood topology derived from up to 68 gene segments and 80 "panarthropod" taxa is shown with bootstrap percentages (BP) displayed above branches. Below branches is displayed the number of gene fragments that individually support the adjoining node with a BP ≥75%. Numbers ≥2 are in boldface. Terminal taxa are labeled by their genus name. Higher classificatory names are also labeled. Those higher-level taxa that were strongly supported and newly named in Regier et al., 2010 [11], plus Symphyla + Pauropoda, are italicized with an open gray circle on the node. In this figure and throughout this report, Pycnogonida: Colossendeidae: *Colossendeis* sp. used in references [5], [11] has been renamed Pycnogonida: Nymphonidae: *Nymphon* sp. due to an original misidentification (see Materials and Methods, Acknowledgments).

The robust resolution of higher-level arthropod phylogeny has been a challenging problem, as evidenced by numerous publications with alternative proposals of relationships [1]-[10]. However, a recent molecular report [11] describes fully resolved relationships within and among four all-inclusive, extant arthropod clades -- Pancrustacea, Myriapoda, Euchelicerata, and Pycnogonida -- with generally high levels of node support, with some exceptions, particularly inside Euchelicerata (redrawn in Figure 1 of this report; see also Table 1 and Materials & Methods for character set definitions used in this and previous reports). The apparent success of this study is likely due to its relatively broad taxon sample (75 arthropod spp. from all major lineages +5-10 diverse outgroup spp.), large data matrix (up to ca. 40 kilobase pairs / taxon from 62 protein-coding nuclear genes), and focus on appropriate methodologies (e.g., likelihood analyses under a codon model and under models that are informed by nonsynonymous change). Reassuringly, high node support is a general feature across a broad range of analytical methods and character codings. Of perhaps greatest taxonomic interest because of their relative novelty are six newly named groups within Pancrustacea (i.e., Altocrustacea, Vericrustacea, Multicrustacea, Communstraca, Miracrustacea, Xenocarda; see Figure 1) plus a group within Myriapoda (i.e., Symphyla + Pauropoda) that receives strong bootstrap support (i.e., ≥80%). While analyses of nucleotides, codons, and amino acids all recover these seven groups in their maximum likelihood topologies, analysis of amino acids is unique in that support for six of the seven is significantly lower than with other approaches, in which bootstrap support is always strong. In that report, it was suggested that the failure of amino acid models to distinguish two clusters of serine codons, standardly called Ser1 (TCN) and Ser2 (AGY), is a cause of lower node support, rather than any problem specific to the nucleotide-based analyses, and this has now been further supported (Zwick, Regier & Zwickl, in preparation). Thus, all analytical approaches now appear to be in close agreement. However, this amino acid issue, among others, highlights the important further question as to what in particular provides the supportive and conflicting signals that determine node support for the higher-level arthropod clades.

**Table 1:** Definitions of *degen1* coding and of character sets.

*degen1*	A coding method for nucleotides in which codons in a data matrix are completely degenerated, e.g., CAT -- > CAY and TTA --> YTN. The consequence of applying this method to nucleotide character sets is that all and only nonsynonymous change between any two sequences in a multisequence alignment can now be parsimony informative.
***nt123***	A data set consisting of all nucleotide characters (after exclusion of a mask) in a multisequence alignment.
***nt1, nt2, nt3***	A data subset consisting of all and only first-, second-, or third-codon-position characters, respectively, in a multisequence alignment.
***LRall1***	A data subset consisting of all and only those *nt1*-characters that encode one or more leucine or arginine residues in a multisequence alignment. Only leucine and arginine codons can directly undergo synonymous change at *nt1*.
***LRall1nt3***	*LRall1 + nt3*.
***noLRall1***	A data subset consisting of all and only those *nt1*-characters that do NOT encode any leucine or arginine residues in a multisequence alignment. In combination, *noLRall1* and *LRall1* constitute *nt1*.
***noLRall1nt2***	*noLRall1 + nt2*. In combination, *noLRall1nt2* and *LRall1nt3* constitute the entire data set, or *nt123*.
***nt123_degen1***	*A nt123* data set subjected to *degen1* coding.
***nt3_degen1***	*A nt3* data set subjected to *degen1* coding.
***LRall1nt3_degen1***	*A LRall1nt3* data subset subjected to *degen1* coding.
***nt3_4foldsynon***	A data subset consisting of all and only those *nt3* characters that are potentially fourfold synonymous, *e.g.*, those encoding glycine and alanine. In *nt3_4foldsynon*, non-degenerate (*i.e.*, tryptophan and methionine) and potentially twofold- and sixfold-degenerate codons are completely degenerated so as to be uninformative.

Taxon sampling is clearly important [12], [13]. No pancrustacean groups other than Pancrustacea itself are strongly supported when taxon sampling is reduced from 80 to 13 species, even when the taxa are represented by an identical gene sample and are similarly analyzed [5], [11].

Features of gene analysis are also important in assessing phylogenetic informativeness. For example, while it is commonly acknowledged that not all genes are equally informative, what is to be made of the finding that six of the seven arthropod nodes mentioned above are never recovered with strong bootstrap support by any of the 62 genes (see Supplementary Table 3 in [11]). A reasonable suggestion would be that their strong node support in the combined-gene analyses results from the cumulative weak signal of multiple genes. This hypothesis raises at least two important questions: 1) How many data are needed to resolve nodes with strong support [14]? and 2) How are we to know whether the cumulative bootstrap signal is actually phylogenetic signal, given the increased sensitivity of large data sets to systematic error, despite their decrease in stochastic error [15]–[18]?

A second feature of gene analysis is that inclusion of more rapidly evolving genes can result in relatively lower node support values for higher-level groupings [19]–[24]. For example, it was found that when the 10 most rapidly evolving genes were deleted from a 13-taxon data matrix, bootstrap support for Hexapoda (represented by 2 spp.) increased from <50% to 79% and for Malacostraca + Copepoda (represented by 2 spp., included in what is now called *Multicrustacea*) from 68% to 97% [5]. But, does this correlation permit a practical generalization for designing data sets for arthropod phylogeny?

A third feature of gene analysis is that synonymous change can have a deleterious effect on inferring higher-level phylogeny [25]–[28], although the appropriateness of their exclusion is controversial [29]–[33]. At least in part, this results from increased compositional heterogeneity of its underlying characters relative to those driving nonsynonymous change [34], [35]. Even modest amounts of synonymous change (e.g., the amount contributed by some first-codon-position characters in a standard first- + second-codon analysis) can result in strong node support for incorrect groups (see Figure 1 in [5]. Such compositional effects are widely acknowledged and documented (e.g., [36–51; but see 52], but even more widely ignored in practice, probably because most readily available software packages do not address the problem. Recently, there has been an increased interest in directly accounting for compositional changes in phylogenetic analyses [53]–[56], but for now this remains a work in progress. Implementation of a codon model can indirectly diminish the contribution of compositional heterogeneity because synonymous change occurs relatively rapidly [57,11 but see 58,59]. A conceptually similar, but much less computationally demanding, approach is to partition data and apply separate models or parameters [26], [28], [32], [60]. When synonymous and nonsynonymous changes are enriched in separate bins, the synonymous change becomes effectively downweighted, an approach that has been shown in Lepidoptera to give results similar to implementation of a codon model, with both approaches showing improvement over likelihood analysis under the unpartitioned GTR + gamma + Inv model [61], [62]. The effect of partitioning on the arthropod data set of Regier et al. (2010) [11] is documented for the first time in the current report.

Two other approaches for reducing the effect of synonymous change have recently been developed specifically for higher-level phylogeny. In one approach, all characters in a data matrix are removed that have the possibility of undergoing synonymous change [5], yielding a so-called *noLRall1nt2* character set (see Table 1 for definition). The other builds on the original idea of *R-Y* coding at the third codon position [63], [44], [64], [65]. In this approach, which we have previously called *degen1* [11], all sites at first and third codon positions that have the potential to undergo synonymous change are individually fully degenerated, yielding a *nt123_degen1 character set* (see Table 1 for character set definitions). In the studies carried out to date, *degen1* generally supports higher-level nodes as well as or better than all other approaches tested, particularly those that include synonymous change [11], [61], [62] (Zwick, Regier & Zwickl, in preparation).

The current report is a further exploration of the characters that have been analyzed in Regier et al. (2010) [11] to infer arthropod phylogeny, with the aim of deciding whether their phylogenetic conclusions remain warranted, particularly as regards the newly named groups. The major issues (re)addressed are 1) the phylogenetic signal of the individual genes and the effect of their rates on node support, 2) the amount of data required to achieve strong node support, 3) the effect of character partitioning, and 4) the contribution of synonymous change to node support.

## Results

### Phylogenetic signal in individual gene segments

A more thorough bootstrap analysis of individual gene segments has been performed than previously [11], but the results remain very similar (Tables S1, S2). For visualization purposes, the number of individual gene segments that support particular nodes with bootstrap values ≥75% is plotted beneath branches on the combined-gene phylogeny (Figure 1). Numerous lower-level and some higher-level groups receive support from multiple individual genes, e.g., up to 43 for Branchiopoda: Anostraca. Notable among the higher-level groups are Arthropoda (4 genes) and Pancrustacea (3 genes). However, many of the nodes along the backbone, including five of the six recently named groups [11] and Symphyla + Pauropoda (see open gray circles in Figure 1), have no individual genes supporting them at that level of the bootstrap. Other higher-level taxonomic groups that have been inconsistently recovered in published studies (i.e., Hexapoda, Oligostraca, Progoneata, Mandibulata) similarly have no individual gene support at that level.

The maximum likelihood tree shown in Figure 1 is based on a combined-gene analysis of the *nt123_degen1* data matrix (see Figure 1 in [11]). In that tree [11], 71 groups out of 78 are recovered by at least three of the four phylogenetic approaches (always including *degen1*,) and all but one of these groups (Oligostraca: Ichthyostraca + Mystacocarida) receive >70% bootstrap by at least one of four implemented approaches (groups listed in Table S1). Sixty-three of these have strong combined-gene bootstrap support (i.e., bootstrap ≥80%) by at least one approach, and 46 of the 63 receive single-gene bootstrap support ≥75% from two or more gene segments. The remaining 17 groups, plus all eight that do not have strong combined-gene bootstrap support, receive ≥75% single-gene bootstrap support from one or no gene segments. The latter includes six of the seven nodes of particular interest.

**Figure 2:**
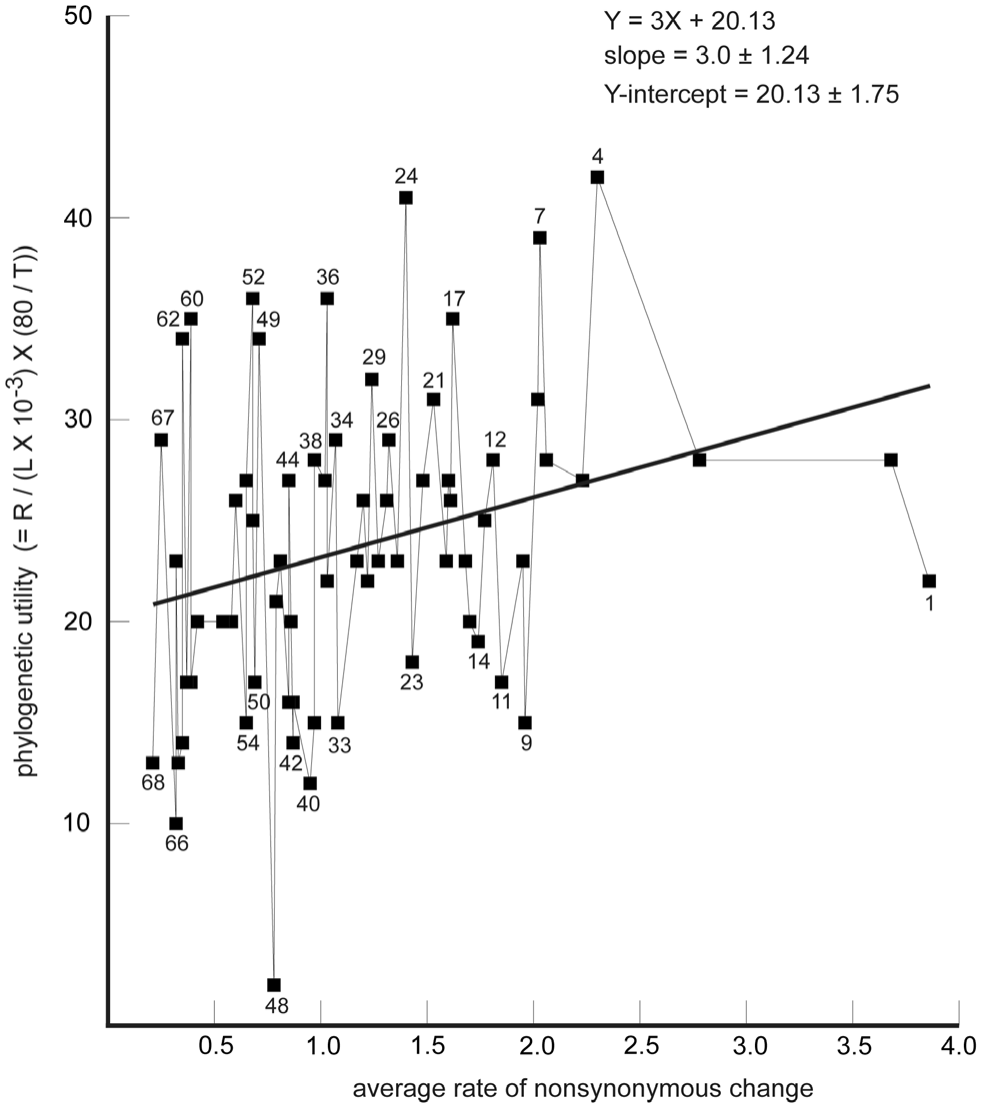
Phylogenetic utility of 68 gene segments plotted relative to its average rate of nonsynonymous change. As described in greater detail in Materials & Methods, phylogenetic utility (units are displayed on Y axis) is the number of taxonomic groups present in Figure 1 that are recovered with BP ≥75% / nucleotides sequenced for that gene segment ×10^−3^, corrected for the fraction of missing data. Units for the average rate of nonsynonymous change for each of the 68 gene segments (displayed on X axis) are the number of substitutions at nt2 per site across a 13-taxon tree, corrected for missing taxa (from Table 2 in [5]). The straight line y  =  bx + c that best fits the data, using linear regression, is shown, together with values for the slope (±1 standard deviation) and the Y-intercept (±1 standard deviation).. For convenient cross-reference and identification, individual gene segments are numbered (1 = fastest, 68 = slowest) as in Table 2 of Regier et al., 2008 [5].

We have defined an approximate metric for the phylogenetic utility of a sequence that takes into account sequence length and number of groups recovered, and that corrects for the variable success rate of amplification and sequencing (see Materials & Methods). This metric has been calculated for each of the 68 gene segments and plotted against its average rate of nonsynonymous change (Figure 2). Over an approximately 18-fold range in average rate, there is a significant (at the level of two standard deviations), but not pronounced, tendency for faster-evolving gene segments to have higher utility. However, there is much scatter, with numerous segments of similar average rates displaying an approximately fourfold difference in utility.

### Quantity of data required for strong node support

A random resampling of characters (also called "sites") without replacement from the complete data matrix has been undertaken to estimate the amount of data needed to achieve the observed levels of combined-gene node support (Figure 1; Tables 2, S3), based on the idea that fewer data of the same sort should provide less phylogenetic signal. As a control, a shuffling and reanalysis of the complete data matrix without data reduction results in bootstrap values that vary by ≤2%. However, when the size of the data matrix is reduced to 85%, three nodes (Ammotheidae, Vericrustacea, Entognatha) show a >10% decrease (maximum of 22% for Vericrustacea) in bootstrap support relative to the original data matrix. With a 50% reduction in size for each of five complementary replicated data sets, bootstrap values of 32 nodes decrease by >10% in one or more of the 10 analyses. (Values <50% are treated as  = 49% for purposes of computation. Given this, no observations about possible major reductions in bootstrap values are made about nodes having values <60%.) Nodes with bootstrap values in the 60 – 99% range for the original data matrix are preferentially affected by 50% reduction in the data set size. In particular, all 29 nodes in this range show at least one instance of a >10% reduction, while only five of the 32 nodes with 100% bootstrap support show such a reduction (Tables 2, S3). Further emphasizing the abruptness of the boundary, 9 of 11 nodes with bootstrap values from 97 – 99% for the original data set show a ≥30% variation in bootstrap support across the 10 pseudo-replicates, while of the 41 nodes with 100% bootstrap in the original, only one (Oligostraca) shows such a high level of variability. Similar observations hold with further reductions in data set size, i.e., to 33% and 15% of the original matrix (Table S3), although of course even more nodes are now affected.

**Table 2:** Highly variable bootstrap values for selected taxa upon analysis of shuffled, half-sized data matrices.^a^

taxonomic group	100% unshuffled	50% shuffled (1)	50% shuffled (2)	50% shuffled (3)	50% shuffled (4)	50% shuffled (5)
Ammotheidae + Endeididae	93	89 – 83	97 – (<50)	95 – 70	(<50) – 96	63 – 95
Ammotheidae	99	100 – 52	72 – 98	86 – 97	94 – 86	89 – 96
*Tanystylum + Achelia*	98	58 – 100	90 – 96	95 – 83	(<50) – 100	86 – 96
Chelicerata	74	54 – 78	62 – 72	69 – 73	67 – 62	(<50) – 90
Pulmonata	65	51 – 89	(<50) – 89	64 – 86	64 – (54)	(<50) – 96
Tetrapulmonata	99	100 – (<50)	100 – 59	99 – 63	91 – 95	91 – 96
Pleurostigmophora	93	91 – 59	82 – 82	79 – 82	77 – 85	92 – 67
Scolopendromorpha + Lithobiomorpha	99	(<50) – 100	87 – 99	78 – 100	96 – 95	100 – 82
Diplopoda	99	50 – 100	78 – 100	74 – 99	77 – 99	95 – 95
Callipodida + Polyzoniida	55	(<50) – (<50)	(<50) – (<50)	(<50) – (<50)	<50 – 53	86 – (<50)
Symphyla + Pauropoda	92	<50 – 73	50 – 84	56 – 83	70 – 64	<50 – 90
Oligostraca	100	95 – 87	99 – 55	95 – 85	84 – 80	84 – 90
Altocrustacea	93	68 – 75	87 – <50	95 – (<50)	<50 – 93	60 – 77
Communostraca	84	<50 – 87	(<50) – 95	88 – (<50)	54 – 82	<50 – 87
Eucarida + Peracarida	87	90 – <50	62 – 94	58 – 69	78 – 68	63 – 86
Sessilia	97	82 – 94	83 – 93	99 – 66	81 – 93	85 – 86
Miracrustacea	94	70 – 58	79 – <50	94 – (<50)	73 – 80	<50 – 92
Xenocarida	93	75 – 78	100 – (<50)	94 – <50	77 – 80	52 – 91
Entognatha	86	74 – 73	74 – 83	63 – 91	88 – 65	(51) – 91
Entomobryomorpha	98	88 – 93	99 – 67	96 – 82	69 – 99	93 – 91
Pterygota	99	77 – 95	93 – 79	96 – 85	50 – 89	82 – 67
Neoptera	97	73 – 91	84 – 75	75 – 89	92 – 73	98 – <50
Blattodea + Orthoptera	94	86 – 84	59 – 98	86 – 84	99 – (<50)	99 – (57)

a This table shows results for only those taxa in the "50% shuffled" analyses that have one or more highly variable (≥30%) bootstrap values. In the five columns at the right (columns 3 – 7) are shown the bootstrap results of five complementary pairs of "50% shuffled" analyses. Complementary results (that is, from different characters within the same bootstrapped data set) are separated by a dash (-). Taxonomic groups not present in the ML topology for that analysis have their bootstrap percentages within parentheses. Results for the complete matrix are shown in the second column for comparison and match those in Figure 1. Complete results for "100% shuffled", "85% shuffled", "50% shuffled", "35% shuffled", and "15% shuffled" are shown in Table S3.

An additional, intriguing result is that there are seven instances affecting five nodes in which bootstrap values show a >10% *increase* with data set reduction relative to the original data set, and this is even more striking when one considers that 59 of the 71 nodes already have values ≥90% and, hence, are excluded from the statistic. The complementary, paired bootstrap value for each of the seven instances is always ≥25% *less* than for the original data set, indicating that phylogenetic signal is not equally distributed across characters and that the most informative characters may be relatively few in number.

### Informativeness of subsets of genes with different average nonsynonymous rates

**Figure 3:**
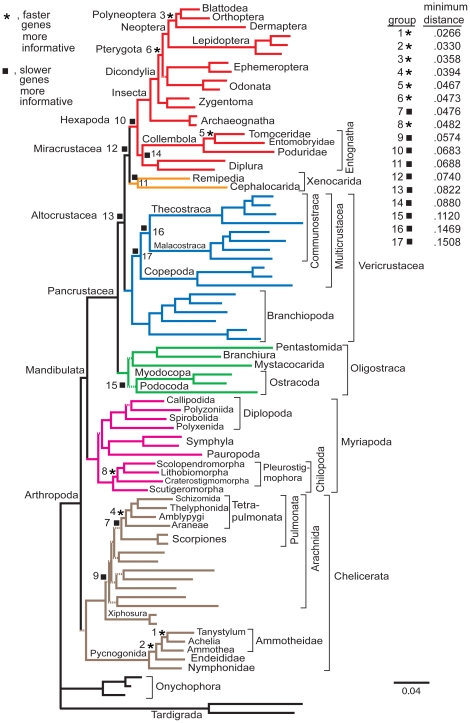
Phylogram of higher-level arthropod relationships based on likelihood analysis of aligned, concatenated *nt123_degen1* sequences. The cladogenic relationships of 80 "panarthropod" taxa based on analysis of 68 gene segments are the same as those in Figure 1. Nodes with BP that are at least 25% higher with the faster genes than the slower genes are marked with an asterisk. Groups with BP that are at least 25% higher with the slower genes than with the faster genes are marked with a filled square. The shortest distance in number of substitutions / character from the base of a group to a terminal taxon in that group ("minimum distance" in inserted table) was calculated by summing the likelihood branch lengths. Groups are numbered from those with the shortest distance to a terminal (no. 1 =  *Tanystylum + Achelia*) to those with the longest distance (no. 17 =  Multicrustacea).

The complete *nt123_degen1* data matrix has been partitioned into two equal-sized submatrices consisting of *faster* and *slower* evolving gene segments (nonsynonymous changes only), and their maximum likelihood topologies and bootstrap values estimated (Tables 3, S4). Seven nodes are recovered with bootstrap values that are ≥30% higher for the *faster* genes than the *slower* genes, while there are 10 nodes for which the *slower* genes have ≥30% higher bootstrap values than the *faster* ones. Nodes for which *slower* genes have higher values tend to have accumulated less overall change (to be more "ancestral") than those for which *faster* genes have higher values (for visual estimate, see Figure 3; for quantitative estimate, see inserted table in Figure 3). Although not as decisive, a *noLRall1nt2* analysis yields similar results (Table S4).

**Table 3:** Bootstrap values for selected taxa that are sensitive to nonsynonymous rate properties of the data matrix.^a^

taxonomic group	*complete*, 1–68 gn, 39261 bp	*faster*, 1–37 gn, 19842 bp	*slower*, 38–68 gn, 19419 bp	*fastest*, 1–24 gn, 13173 bp	*middle*, 25–43 gn, 12834 bp	*slowest*, 44–68 gn, 13254 bp
1: Ammotheidae + Endeidae	93	91	57	99	86	(<50)
2: Nymphonidae + Endeididae	(<50)	(<50)	(<50)	(<50)	(<50)	82
Ammotheidae	99	79	95	87	(<50)	60
*Tanystylum + Achelia*	98	97	68	99	81	(<50)
1: Chelicerata	74	73	55	53	96	(<50)
2: Arthropoda minus Pycnogonida	(<50)	(<50)	(<50)	(<50)	(<50)	91
Arachnida	68	(<50)	89	(<50)	(<50)	83
Pulmonata	65	(<50)	93	56	57	94
Tetrapulmonata	99	99	67	92	76	67
Pleurostigmophora	93	94	<50	95	(<50)	(<50)
Scolopendromorpha + Lithobiomorpha	99	99	88	89	95	52
1: Progoneata	67	64	(<50)	74	(<50)	(<50)
2: Chilopoda + Diplopoda	(<50)	(<50)	58	(<50)	<50	79
Diplopoda	99	98	83	95	(<50)	97
1: Callipodida + Polyzoniida	55	(<50)	72	(<50)	99	(<50)
2: Spirobolida + Polyzoniida	(<50)	(<50)	(<50)	(<50)	(<50)	93
3: Callipodida + Spirobolida	(<50)	<50	(<50)	55	(<50)	(<50)
Symphyla + Pauropoda	92	59	78	65	(<50)	88
Ostracoda	60	(<50)	85	(<50)	(<50)	95
Altocrustacea	93	<50	94	(<50)	<50	88
Phyllopoda	100	100	100	100	61	100
Multicrustacea	100	<50	100	65	<50	98
Communostraca	84	(<50)	94	(<50)	(<50)	95
Sessilia	97	93	82	97	(<50)	87
Miracrustacea	94	(<50)	98	(<50)	<50	91
Xenocarida	93	(<50)	98	(<50)	76	94
Hexapoda	100	64	100	<50	100	100
Entognatha	86	<50	88	<50	(<50)	91
Entomobryomorpha	98	99	(<50)	93	90	(<50)
Pterygota	99	96	71	80	80	<50
Neoptera	97	77	96	(<50)	91	78
Polyneoptera	99	100	(<50)	100	96	(<50)
Blattodea + Orthoptera	94	76	94	86	(<50)	86

a This table shows results only for those taxa that show sensitivity in their bootstrap values to the rate properties of the underlying data submatrix. All submatrices are fully degenerated ("*degen1* coding"). The results for all taxa are shown in Table S3. Results for the complete matrix are shown for comparison in the second column and match those in Figure 1. Columns 3 and 4 show the results of splitting the complete data matrix into two approximately equal-sized subsets (referred to as *faster* and *slower* in the text). Column 5–7 show the results of splitting the complete data matrix into three approximately equal-sized subsets (referred to as *fastest, medium, slowest* in the text). Alternative groupings for three taxa in Figure 1, namely, Ammotheidae + Endeididae, Chelicerata, Progoneata, and Callipodida + Polyzoniida, are also included because they receive strong support in the tripartite division of the complete data matrix (see text). Bootstrap values for nodes not recovered in the maximum-likelihood topology of a particular analysis are within parentheses. bp, base pair; gn, gene segment.

The findings are similar, although slightly more complicated, when the original *nt123_degen1* matrix is partitioned into three equal-sized submatrices -- *fastest*, *medium*, and *slowest* (Tables 3, S4). The *fastest* and *slowest* results are largely unchanged from those for the *faster* and *slower* submatrices. Additionally however, there are three new nodes for which the *medium* matrix yields bootstrap values that are ≥30% higher than in the *fastest* or *slowest* (both in two of three cases) matrices. Only one of these three nodes receives strong bootstrap support with the complete (*nt123_degen1*) data matrix, although all do in the *medium* analysis. Conversely, there are four cases for which the *medium* matrix yields bootstrap values that are ≥30% *lower* than in the *fastest* or *slowest* matrices. All four of these nodes fail to receive strong support in the *medium* analysis, but three receive strong support with the complete data set, and the fourth nearly so. Results with *noLRall1nt2* data sets yield similar results (Table S4).

Does partitioning by rate lead to strong support for any groups that conflict with those recovered with the complete data set? Indeed, there are four instances of this (Tables 3, S4): 1) 82% bootstrap support for Nymphonidae + Endeididae with the *slower* genes versus 93% bootstrap support for Ammotheidae + Endeididae (all Pycnogonida) with the complete matrix; 2) 91% bootstrap support for Arthropoda minus Pycnogonida with the *slower* genes versus 74% bootstrap support for Chelicerata with the complete matrix; 3) 93% bootstrap support for Spirobolida + Polyzoniida with the *slower* genes versus 67% bootstrap support for Callipodida + Polyzoniida (all Diplopoda) with the complete matrix; and 4) 79% bootstrap support for Chilopoda + Diplopoda with the *slower* genes versus 67% bootstrap support for Progoneata (all Myriapoda) with the complete matrix.

### Utility of data partitioning for phylogenetic reconstruction

Partitioning the *nt123_degen1* data matrix (without exclusion other than unalignable portions) into two or three linked submatrices that differ in their average rate of nonsynonymous change yields no nodes whose bootstrap values vary by >10% from the unpartitioned *nt123_degen1* analysis (Table S4). By contrast, when *nt123* is partitioned into two linked submatrices that largely separate synonymous and nonsynonymous change (i.e., *noLRall1nt2, LRall1nt3*), there is a substantial improvement over the unpartitioned *nt123* results. In particular, seven nodes in the *nt123* partitioned analysis yield bootstrap values that are >10% higher than *nt123* unpartitioned, while there are none lower by >1%. Even so, the *nt123_degen1*, unpartitioned analysis yields 28 nodes that have >10% higher bootstrap values than in the *nt123*, partitioned analysis, while only one is >10% lower (a generally problematic node at that, namely, Arthropoda minus Pycnogonida).

### Testing the informativeness of synonymous signal

**Figure 4:**
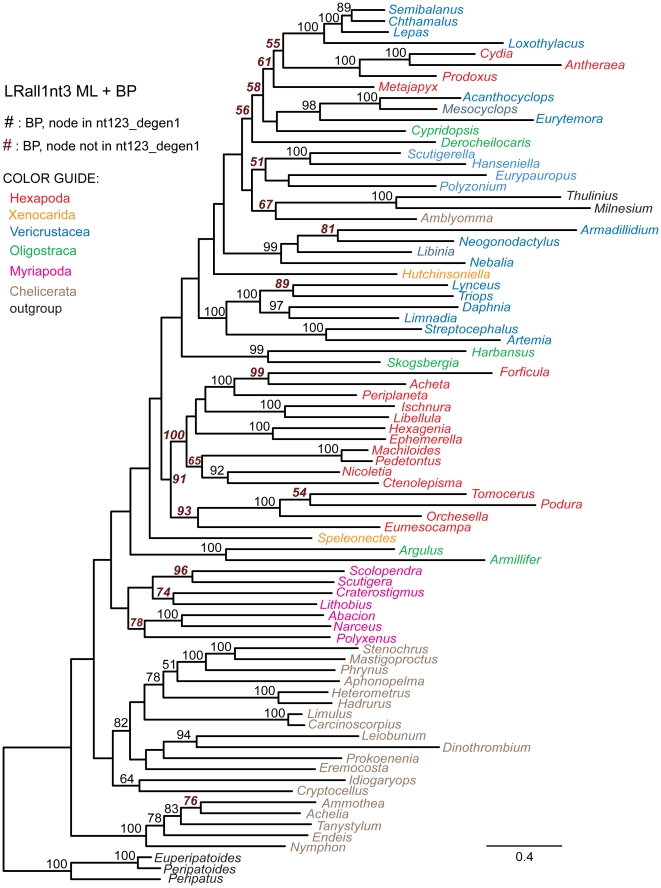
Phylogram derived from likelihood analysis of the *LRall1nt3* character coding of the 68 gene segments. Bootstrap values ≥50% are displayed above internal branches. All and only those bootstrap percentages that correspond to nodes not present in the *nt123_degen1* topology (Figure 1) are in italics, boldface, and brown in color. Notice that the branch lengths for the degenerated data matrix *LRall1nt3_degen1* (Figure 5) are much shorter than in this figure. Units are substitutions / character.

**Figure 5 pone-0023408-g005:**
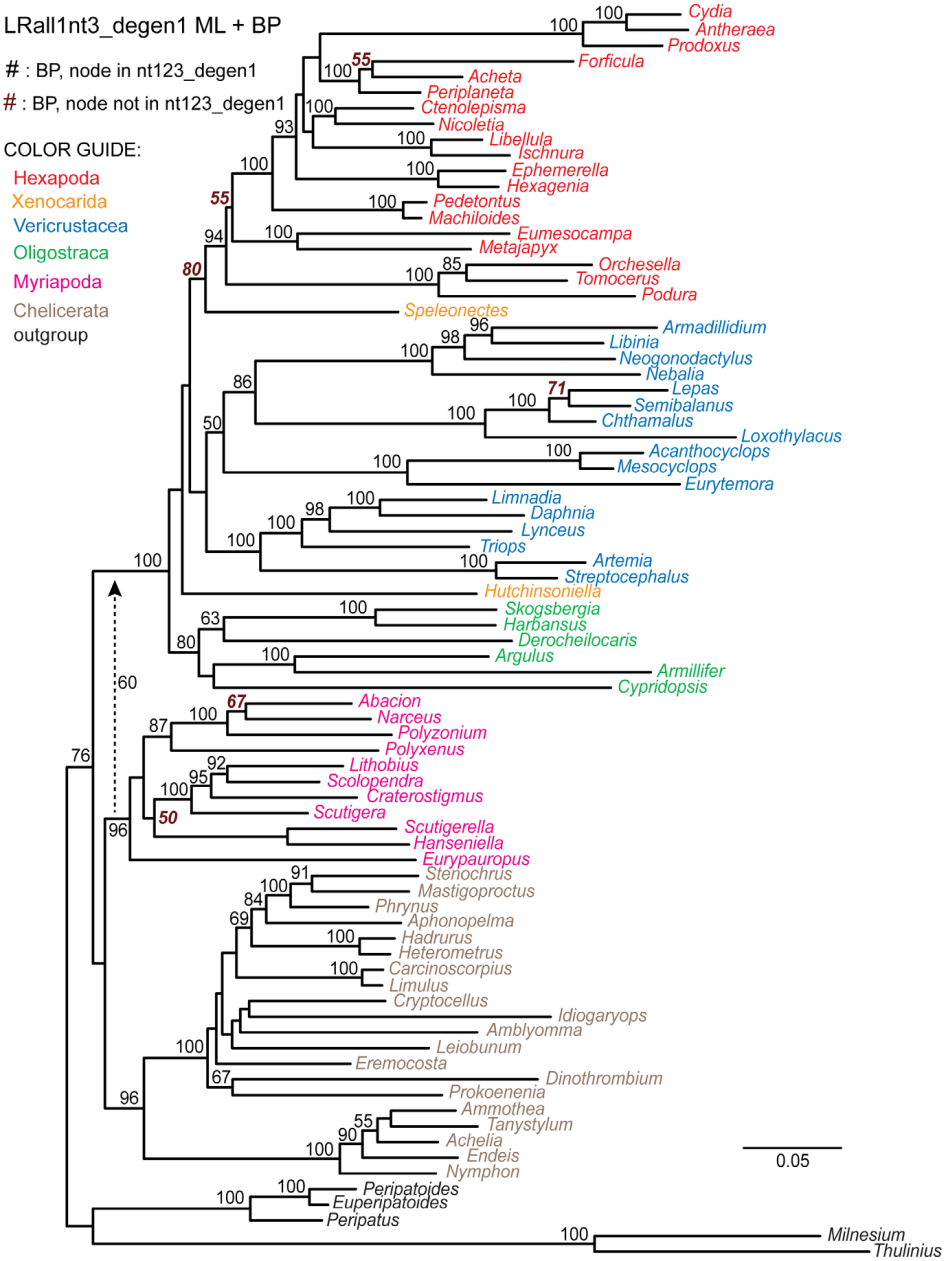
Phylogram derived from likelihood analysis of the *LRall1nt3_degen1* character coding of the 68 gene segments. Bootstrap values ≥50% are displayed above internal branches. All and only those bootstrap percentages that correspond to nodes not present in the *nt123_degen1* topology (Figure 1) are in italics, boldface, and brown in color. Notice that the branch lengths for the non-degenerated data matrix *LRall1nt3* (Figure 4) are much longer than in this figure. Units are substitutions / character.

The data matrix of Regier et al. (2010) [11] provides a convenient venue for testing the informativeness of synonymous change in supporting ancient divergences. To do this, several approaches have been taken to separate synonymous and nonsynonymous signals, using as a reference the analysis of the *nt123_degen1* data matrix, which yields 63 strongly supported nodes (Figure 1). Comparisons of particular note are summarized in Table 4, while the complete trees and their node support values are displayed (Figures 1, 4–8). The *noLRall1nt2* data matrix, which largely undergoes only nonsynonymous change, yields 59 strongly supported nodes, and all are in agreement with the *nt123_degen1* result (Table 4). The matrix that is complementary to *noLRall1nt2*, called *LRall1nt3* and which is enriched in synonymous change, is approximately equal in size to *noLRall1nt2* but recovers only 31 strongly supported nodes that are in agreement with the *nt123_degen1* result, plus seven others that strongly conflict (Table 4, Figure 4). Although *LRall1nt3* is greatly enriched for synonymous change, some nonsynonymous signal remains. This is directly demonstrated by analysis of the *LRall1nt3_degen1* data matrix, in which the number of strongly recovered nodes in agreement with *nt123_degen1* increases to 47, while the number of strongly conflicting nodes decreases to one (Table 4, Figure 5).

**Table 4:** Comparison of synonymous and nonsynonymous change in terms of node recovery and node support.

character set	characters (kbp)^a^	conflicting nodes^b^	strongly conflicting nodes^c^	strongly supporting nodes^d^
nt123_degen1^e^	39.3	N.A.	N.A.	(63)
noLRall1nt2	21.8	0	0	59
LRall1nt3	17.5	18	7	31
LRall1nt3_degen1^e^	17.5	6	1	47
nt3	13.1	15	6	19
nt3_degen1^e^	13.1	3	1	31
nt3_4foldsynon^e^	13.1	5	1	10

a The total number of characters in each data set in kilobase pairs (kpb).

b The number of dichotomous nodes with bootstrap values ≥50% in the likelihood analysis of the designated character set that fails to match any of the 63 nodes with bootstrap values ≥80% in the analysis of the *nt123_degen* character set (see Figure 1).

c The number of nodes with bootstrap value ≥80% in the likelihood analysis of the designated character set that fails to match any of the 63 nodes with bootstrap values ≥80% in the analysis of the *nt123_degen* character set (see Figure 1).

d The number of nodes with bootstrap values ≥80% in the analysis of the designated character set that matches one of the 63 nodes with a bootstrap value ≥80% in the analysis of the *nt123_degen* character set (see Figure 1).

e These four character sets contain numerous polymorphic character states.

**Figure 6 pone-0023408-g006:**
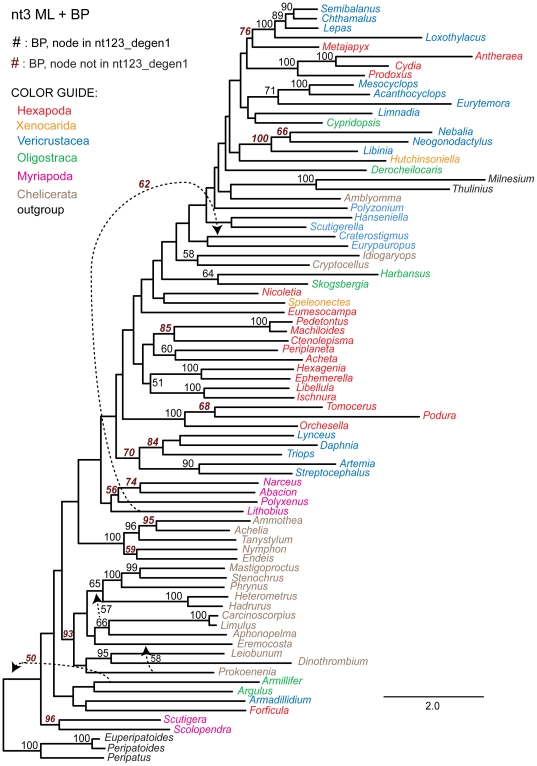
Phylogram derived from likelihood analysis of the *nt3* character coding of the 68 gene segments. Bootstrap values ≥50% are displayed above internal branches. All and only those bootstrap percentages that correspond to nodes not present in the *nt123_degen1* topology (Figure 1) are in italics, boldface, and brown in color. Notice that the branch lengths for the degenerated data matrix *nt3_degen1* (Figure 6) are much shorter than in this figure. Units are substitutions / character.

**Figure 7 pone-0023408-g007:**
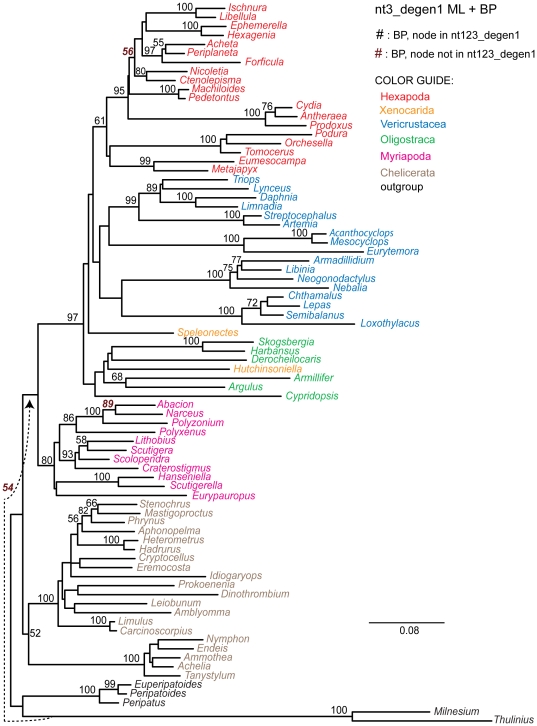
Phylogram derived from likelihood analysis of the *nt3_degen1* character coding of the 68 gene segments. Bootstrap values ≥50% are displayed above internal branches. All and only those bootstrap percentages that correspond to nodes not present in the *nt123_degen1* topology (Figure 1) are in italics, boldface, and brown in color. Notice that the branch lengths for the non-degenerated data matrix *nt3* (Figure 6) are much longer than in this figure. Units are substitutions / character.

To further assess the value of synonymous change, the *nt3* data matrix was analyzed (Table 4, Figure 6). Now, only 19 nodes in agreement with *nt123_degen1* are strongly supported, plus six more that strongly conflict. However, this data set too still contains nonsynonymous signal, albeit even less than that in *LRall1nt3*. To demonstrate this, analysis of the *nt3_degen1* data matrix, which is almost entirely polymorphic (i.e., almost no A, C, G or T) nevertheless recovers 31 strongly supported nodes in agreement with *nt123_degen1*, plus only one that strongly conflicts (Table 4, Figure 7).

**Figure 8:**
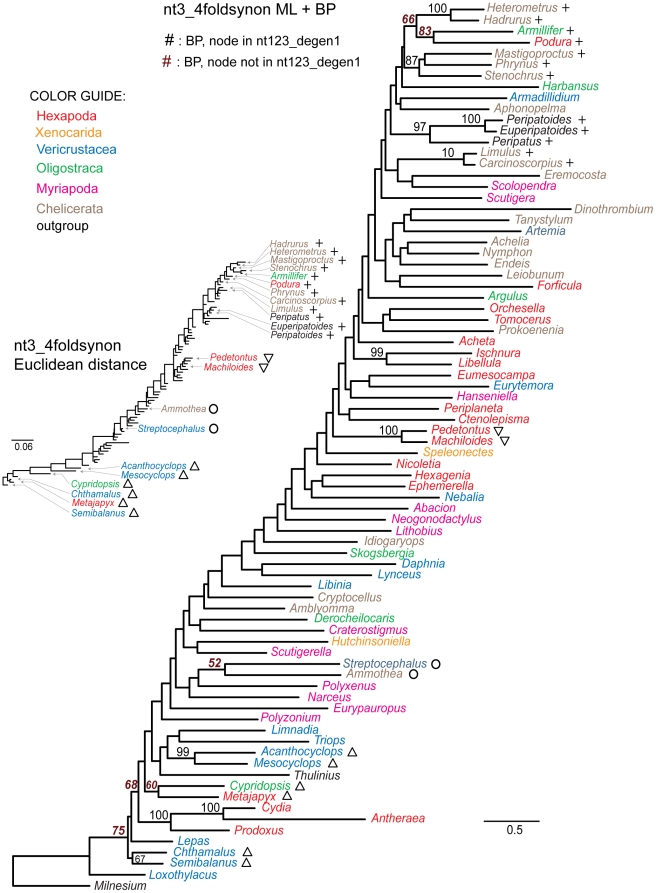
Phylogram and Euclidean distances derived from analysis of the *nt3_4foldsynon* character coding. In the main figure, bootstrap values ≥50% are displayed above internal branches of the phylogram based on likelihood analysis of the 68 gene segments. All and only those bootstrap percentages that correspond to nodes not present in the *nt123_degen1* topology (Figure 1) are in italics, boldface, and brown in color. Units for the phylogram are substitutions / character. In the smaller, inserted figure are shown Euclidean distances based solely on nucleotide composition. Symbols (+, ∇, O, Δ) identify the same clusters of taxa in both the ML topology and the Euclidean distance diagram for convenient cross-reference. Units for the Euclidean distance diagram are per cent.

In order to restrict the analysis almost entirely to synonymous change, all character states at *nt3* positions that do not encode a fourfold synonymous codon were fully degenerated (i.e., coded as *N* making them uninformative), and the resulting *nt3_4foldsynon* data matrix was analyzed (Table 4, Figure 8). Under these conditions, only 10 nodes in agreement with *nt123_degen1* are strongly supported, plus one that strongly conflicts. None of these nodes defines a group with more than three terminal taxa, consistent with the hypothesis that synonymous change significantly supports only more recently derived nodes.

A Euclidean distance analysis of the nucleotide composition of *nt3_4foldsynon* shows evidence of strong compositional heterogeneity, when the absolute values of these distances are compared with those in other studies (cf. insert to Figure 8 and [66]). Furthermore, shared bias, as evidenced by the Euclidean tree (Figure 8, insert), correlates with, and is a reasonable explanation of, almost all of the nodes with bootstrap values ≥50% that conflict with the *nt123_degen1* likelihood analysis, including

- 83% bootstrap: *Armillifer* (Oligostraca: Pentastomida) + *Podura* (Hexapoda: Collembola);
- 66%: *Armillifer* (Oligostraca: Pentastomida) *+ Podura* (Hexapoda: Collembola) *+ Heterometrus* (Arachnida: Scorpiones) *+ Hadrurus* (Arachnida: Scorpiones);
- 52% bootstrap: *Streptocephalus* (Branchiopoda: Anostraca) + *Ammothea* (Pycnogonida); and
- 60%: *Cypridopsis* (Ostracoda: Podocopa) + *Metajapyx* (Hexapoda: Japygidae).

Shared bias in composition may also at least partially explain the level of bootstrap support for many of the presumably correct groupings, including

- 100%: *Heterometrus + Hadrurus* (both Arachnida: Scorpiones);
- 87%: *Stenochrus + Mastigoproctus + Phrynus* (all Arachnida: Pedipalpi);
- 100%: *Peripatoides + Euperipatoides* (both Onychophora: Peripatopsidae);
- 97%: *Peripatoides + Euperipatoides* + *Peripatus* (all Onychophora);
- 100%: *Limulus + Carcinoscorpius* (both Xiphosura);
- 100%: *Pedetontus + Machiloides* (both Hexapoda: Archaeognatha);
- 99%: *Acanthocyclops + Mesocyclops* (both Copepoda: Cyclopoida; and
- 67%: *Chthalamus + Semibalanus* (both Thecostraca: Sessilia).

### Testing the informativeness of indels

An inspection was undertaken of a slightly realigned arthropod data matrix (see Dataset S1 in SUPPORTING INFORMATION] for introns whose evolutionary history appeared to be highly constrained. The results are summarized in Table 5 (see Discussion).

**Table 5:** Occurrence of "high quality" indels in the arthropod data set.^a^

Taxonomic groups recovered	Number of indels
Hexapoda: Collembola^b^	2
Hexapoda: Odonata^b^	2
Hexapoda: Lepidoptera^b^	1
Pancrustacea: Copepoda^b^	1
Pancrustacea: Branchiopoda: Diplostraca^b^	2
Pancrustacea: Copepoda: Cyclopoida^b^	1
Pancrustacea: Malacostraca^b^	1
Arthropoda / Onychophora + Tardigrada^b^	1
Onychophora^b^	1
Pycnogonida: Nymphonidae + Arachnida: Pseudoscorpiones^c^	1
Hexapoda: Collembola: Tomoceridae + Hexapoda: Collembola: Poduridae^c^	1
Pancrustacea: Ostracoda: Podocopa + Tardigrada: Parachela^c^	1
Hexapoda: Ephemeroptera + Hexapoda: Diplura^c^	1
Pancrustacea: Oligostraca + Arachnida: Opiliones^c^	1
Pancrustacea: Copepoda + Ostracoda: Myodocopa^c^	1
Arachnida: Tetrapulmonata + Arachnida: Pseudoscorpiones^c^	1

a "High quality" indels are those that have relatively unambiguous alignments -- no staggered ends and identical lengths but not necessarily identical sequences (see Materials & Methods).

b Present in Figure 1 and considered a valid taxonomic group.

c Not present in Figure 1 and unlikely to be a valid taxonomic group.

## Discussion

### The challenges of higher-level arthropod systematics

Morphology and gene-based studies of higher-level arthropod phylogeny have been ongoing for more than 75 and 20 years, respectively [67]–[69], stimulated by increasing evolutionary and paleontological knowledge of arthropods and their huge radiation at the species level, and also by numerous major findings of developmental biologists (summarized in [70], [71]. However, agreement on many higher-level relationships remains problematic, even with spectacular improvements in methodologies (e.g., polymerase chain reaction and pyrosequencing) and phylogenetic theory (e.g., better likelihood models and faster algorithms). Recently, however, Regier et al., 2010 [11] provided strong evidence for many higher-level arthropod relationships outside Chelicerata, based on analysis of 62 protein-coding nuclear genes (shown in modified form in Figure 1). The current report is a further analysis of their data matrix and results in order to probe whether or not that report represents a major advance in our knowledge of arthropod phylogeny, an overinterpreted and failed report, or something in-between.

An intriguing aspect of Figure 1 is that almost all nodes, excepting a few within Chelicerata, receive strong bootstrap support. Such strong support is oftentimes interpreted as providing a good indication that there is a strong signal supporting those relationships [72], and larger data sets should on average have stronger signal. Of course, decisiveness does not necessarily translate to accuracy [18], but then what might be the source of such strong signal other than phylogeny? First however, we emphasize that the bootstrap percentage is a conservative metric relative to the posterior probability metric generated in Bayesian analyses [73], [74]. This is often obvious in studies that show support values from maximum likelihood and Bayesian analyses for a common topology, e.g., the analytical results for the data set in Regier et al., 2010 [11]. Given the still unresolved controversy over potentially inflated posterior probabilities [75], we feel that support for phylogenies based on Bayesian posterior probabilities alone warrants substantial skepticism and, therefore, was not used in this report. However, we note that a Bayesian analysis of the *nt123_degen1* data set yielded posterior probabilities of 1.0 for all nodes within Mandibulata except one (see Supplementary Figure 3 in [11]).

Compositional heterogeneity is a possible explanation for high support values and is known to be a widespread cause of phylogenetic inaccuracy (e.g., [36–51, but see 52]. In the present study, however, this is highly unlikely to be the explanation for the very widespread strong support because three of the four favored analytical approaches eliminate the largest source of the problem, namely, synonymous change, and the fourth, namely, implementation of the codon model, functionally "downweights" synonymous change due to its relatively rapid evolution [11].

Another potential non-phylogenetic source could be problematic modeling of evolutionary change. However, model testing was performed [76], and the four favored analytical approaches use three different types / classes of models (nucleotide, codon, amino acid models), yet still recover nearly identical topologies [11]. Furthermore, partitioning the nonsynonymous data, which are all of the same sort, namely, rather conservative, protein-coding nuclear gene sequences, yielded no major improvement (Table S4).

While other artifacts remain possible, e.g., [77], [78], if the relationships shown in Figure 1 are generally accurate, then why have many of the higher-level relationships not previously been strongly supported? The current study demonstrates that insufficient quantity of data is likely to be one of the main factors. Of the 68 gene segments tested, none individually support with bootstrap values ≥75% deep nodes like Mandibulata, Oligostraca, Hexapoda, Symphyla + Pauropoda, or five of the six newly named pancrustacean groups, even though all are supported in the combined-gene analysis with bootstrap values >80%. A reasonable interpretation is that their strong support in the combined-gene analyses results from the cumulative effect of small amounts of phylogenetic signal from multiple genes. Earlier studies were restricted to fewer genes and taxa than in Regier et al., 2010 [11], so it may not be surprising that these groups were not strongly supported. Hexapoda deserves special comment, since there has been controversy as to its monophyly (e.g., see [79]). Our results are quite definitive on this matter. Hexapoda is strongly supported as monophyletic, although it is a "difficult" node because no single gene yields a bootstrap value ≥75%. Clear support for hexapod monophyly is similarly difficult to find in morphological analyses, despite its widespread acceptance. We also note that hexapod monophyly can be strongly supported based on analysis of *nt123*, but, at least for the current gene set, this high level of support is largely the result of the unusually biased nucleotide composition at *nt3* and *LRall1* that positively reinforces phylogenetic signal [5).

An alternative interpretation for the paucity of strong support at the single-gene level is that some or all of the genes contribute substantial conflicting signal that cancels the phylogenetic signal, keeping individual node support low and raising doubts about the ultimate source of the combined-gene support. But this seems unlikely to be a predominant explanation because individually the gene segments mostly strongly support groups present in Figure 1 (Table S1). And, for the few cases in which conflicting groups are supported, this support typically stems from only a single gene fragment (Table S2). Further, little conflict is apparent when randomly subsampled matrices that are 15% – 85% of the full data set in size are analyzed. The most significant one is Arthropoda minus Pycnogonida (77% bootstrap) that conflicts with Chelicerata (74% bootstrap).

Submatrices corresponding to different rate categories of genes similarly do not provide striking evidence for major internal conflict. Only four groups conflict with ones in the complete matrix (Pycnogonida: Nymphonidae + Endeididae versus Pycnogonida: Ammotheidae + Endeididae; Arthropoda minus Pycnogonida versus Chelicerata; Diplopoda: Spirobolida + Polyzoniida versus Diplopoda: Callipodida + Polyzoniida; and Chilopoda + Diplopoda versus Progoneata). And only the first one yields strong support for both (conflicting) alternatives. More importantly for this report, none of these groups conflict with any of the six newly named groups, Symphyla + Pauropoda, Mandibulata, Oligostraca, or Hexapoda.

Although not a formal optimality criterion, consistency of results across different analytical approaches is also quite striking in Regier et al., 2010 [11]. Two approaches, called *degen1* and *noLRall1nt2*, analyze in a likelihood framework those nucleotides that undergo mostly nonsynonymous change. A related approach -- likelihood analysis of amino acid change -- is conceptually similar, but of course the underlying model is very different. The fourth approach -- likelihood analysis of codon change -- is different in that codons, not single nucleotides, are analyzed and no data, including those that undergo synonymous change, are excluded from the analysis. Importantly, all four methods largely recover the same groups, including Symphyla + Pauropoda, Mandibulata, Oligostraca, Hexapoda, and the six newly named pancrustacean groups.

So, is the vast majority of conclusions in Regier et al., 2010 [11] credible? Beyond the philosophical point that *all* scientific conclusions are defeasible, it is clear that nodes recovered with the complete data matrix with <100% bootstrap support are more sensitive to data subsampling than those with 100% support. Furthermore, the definition of 80% bootstrap support as "strong" is a heuristic device only; it does not refer to some innate or even broadly accepted category. Therefore, it seems likely that strongly supported nodes in the range of 80 - 99% bootstrap can still be sensitive to changes in the amount of data. Had the amount of data been less, the probability of recovering that group with strong support would have been reduced (Tables 2, S3). By contrast, many fewer of the nodes with 100% bootstrap are sensitive to data subsampling at the levels tested in this report. Even when the data set is reduced to 15% of the complete matrix, there are still 23 of 32 nodes that receive 100% bootstrap support, although none correspond to the deep nodes Symphyla + Pauropoda, Mandibulata, Oligostraca, Hexapoda, and the six newly named pancrustacean groups. What this suggests is that, had fewer data been generated and analyzed in Regier et al, 2010 [11], it is likely that at least some of these high-interest groups would not have been strongly supported. Therefore, given that their strong recovery is near the limit of an admittedly arbitrary designation of "strong", it would still be valuable to generate even more data as confirmation. This is despite the lack of even modest support for alternatives to these high-interest groups, unlike the more problematic situation with Chelicerata and the placement of Pycnogonida [80], [81].

### Nonsynonymous rates and information content

While analyses of individual gene segments fail to recover most deep-level nodes with bootstrap values ≥75%, including five of the six newly named groups [11] plus Symphyla + Pauropoda, Mandibulata, Oligostraca, and Hexapoda, nevertheless, many other nodes are supported at this level (Figure 1). The total number of individual gene segments that recover particular nodes varies dramatically (from 0 to 43, see Table S1). In an effort to capture an assessment of individual gene utility, a statistic was devised that expresses the total number of nodes supported by a single gene segment after adjusting for differing sequence lengths and amount of missing data, and this was expressed relative to the gene segments' average rates of nonsynonymous change (Figure 2; other metrics are possible, e.g., [82], [83]). While a weak trend is apparent for faster genes to have higher utility (ca. 40% increase in utility with a fourfold increase in rate), it is obvious that variability in the results is much greater and likely reflects intrinsic differences not captured by their average nonsynonymous rate. This line of inquiry was not pursued further largely because the taxonomic groups of most interest were not sufficiently strongly supported in these individual gene studies.

To improve the statistical properties of the analyses, subsets of gene segments with similar rate properties were analyzed and compared (Figure 3; Tables 3, S4; see also [5]). In the analysis of the *faster* and *slower* gene subsets, 17 taxonomic groups show large (≥30%) differences in their relative bootstrap support values of total data (nt123) analyses. Interestingly, 9 of these groups are more strongly supported by the *slower* genes, and all 9 have longer node-to-terminal branch lengths than the other eight, supporting the idea that more slowly evolving genes are better for recovering deeper nodes [19]–[24]. However, some clearly ancient nodes, e.g., Pancrustacea and Arthropoda, are strongly supported by both subsets, demonstrating that *faster* genes can remain informative at deeper levels.

When gene segments are partitioned into three subsets (*fastest, medium, slowest*), additional resolution becomes apparent in that three nodes (Chelicerata, Diplopoda: Callipodida + Polyzoniida, Neoptera) receive strong support from the *medium* subset of genes, but mystifyingly four nodes (Diplopoda, Branchiopoda: Phyllopoda, Thecostraca: Sessilia, Insecta: Blattodea + Orthoptera) receive strong support from the *fastest* + *slowest* genes, excluding the *medium* genes.

What is perhaps more noteworthy is that the 3-subset studies reveal underlying conflict in the signal that supports four taxonomic groups.

1. Ammotheidae + Endeidae is strongly supported by the *fastest + medium* genes, while Nymphonidae + Endeididae is strongly supported by the *slowest* genes.
2. Chelicerata is strongly supported by the *medium* genes, while Arthropoda minus Pycnogonida is strongly supported by the *slowest* genes.
3. Progoneata is supported (74% bootstrap) by the *fastest* genes, while Chilopoda + Diplopoda is supported (79% bootstrap) by the *slowest* genes.
4. Callipodida + Polyzoniida is strongly supported by the *medium* genes, while Spirobolida + Polyzoniida is strongly supported by the *slowest* genes.

Based on these observations, it would seem prudent to abstain from any strong conclusion about these conflicting groups, although only one of these four (Ammotheidae + Endeidae) is strongly supported in the complete-gene analysis (Figure 1). No other nodes reveal such strong conflicts.

### The utility of data degeneration, data exclusion, and partitioning by rate on phylogenetic accuracy

Elsewhere, we have justified the utility of excluding synonymous change in our *degen1* and *noLRall1nt2* analyses when analyzing deep-level arthropod relationships [61], [62] (Zwick, Regier & Zwickl, in preparation). A comparison of results from the analysis of *nt123* and *nt123_degen1* in this report reinforces this point (Table S4). Bootstrap support for 31 nodes (out of 71 analyzed) is lower by >10% points with *nt123* than with *nt123_degen1*. By contrast, only three nodes are >10% higher with *nt123*. One (Chilopoda + Diplopoda) is likely to be incorrect, based on consistent, but modestly supported, recovery of Progoneata (Figure 1; Figure 1 in [11]). Another (Arthropoda minus Pycnogonida) is questionable but possible [80], given the striking instance of its receiving strong *degen1* support (bootstrap 91% for the *slowest* genes, Table S4), plus the generally modest *degen1* support for Chelicerata, although the *medium* genes recover Chelicerata with bootstrap 96% (Table S4). And the third (Pulmonata) is possibly correct based on its consistent and modest-to-strong *degen1* support (Tables 3, S4; Figure 1; Figure 1 in [11]), but remains in conflict with the current hypotheses based on morphology [84].

As an alternative to data degeneration (e.g., *nt123_degen1*) and exclusion (e.g., *noLRall1nt2*), we have tried partitioning nt123 into mostly nonsynonymous and most synonymous change (*noLRall1nt2 + LRall1nt3*), and this results in a modest improvement relative to *nt123*, unpartitioned (Table 4). Now, only 27 nodes show >10% lower bootstrap support relative to *nt123_degen1*. However, the take-home message even with this further analysis remains unchanged: synonymous change hinders deep-level phylogenetic analysis (see also discussion below).

Restricting the analysis largely to nonsynonymous change through degeneration, we have asked whether partitioning the entire data set by the average rates of evolution for the different gene fragments, followed by their separate modeling, could provide a benefit. The answer is that, whether split into two or three rate categories, there are no nodes whose bootstrap values differ by >10% from *nt123_degen1*, unpartitioned. Even so, it is also clear that the separate rate categories of genes, as well as individual gene segments, do have distinct signals and can provide dramatically differing levels of support for distinct nodes. Together, these two observations suggest that, in the current case with the complete *nt123_degen1* data set, applying a single model to the entire data set is about as good as using multiple, independent models.

As just mentioned, an important finding of the current study is that individual gene segments and separate rate categories of genes are informative at different phylogenetic levels. In particular, more slowly evolving genes (only nonsynonymous changes considered) tend to support nodes that are closer to the backbone on the phylogram shown in Figure 3, matching the conventional wisdom that slow genes are better for supporting deeper nodes [19], [20], [5], [21]–[24]. Conversely, faster genes provide more support for nodes that are more recently derived.

### The (non)utility of synonymous change for inferring deep-level arthropod phylogeny

As we have documented in this report, synonymous change, much more so than nonsynonymous change, can be misinformative of the correct phylogeny (Table 4). While this is oftentimes attributed to its more rapid evolution, that in itself is unlikely to be an adequate explanation, since, for example, likelihood methods do not require equal rates of change across the character matrix in order to make accurate inferences. Rather, there must be a bias introduced by synonymous change that might then be compounded by faster evolution. Nucleotide compositional heterogeneity is one such bias, and it has been well documented that this is a more dominant feature of characters undergoing synonymous than nonsynonymous change [25]–[28], [5]. The likelihood models that we have used assume a single nucleotide composition, biased or not, across the entire data set. While there is increasing interest in modeling compositional heterogeneity, it is still a work in progress [53]–[56]. The current methodological inadequacy can be seen as one justification for our alternative approach of using nucleotide degeneration or exclusion. However, there is still an outstanding question, which is, how informative of deep-level arthropod phylogeny is synonymous change?

To test the informativeness of synonymous change, we have generated data sets that are progressively more enriched in their potential for synonymous change (Table 4; Figures 4–8; cf. Figure 1). What we observe is that the number of nodes which are strongly supported decreases as the fraction of synonymous change increases, and that strongly supported *incorrect* nodes are almost entirely due to synonymous change. For example, while it has been stated that *nt3* characters should not be removed because they contribute useful phylogenetic information [29]–[33], in the current case *nt3* strongly supports only 19 of 63 nodes strongly supported by *nt123_degen1*, plus 15 conflicting nodes (6 strongly conflicting), while *nt3_degen1*, which is almost entirely nonsynonymous and polymorphic, still strongly supports 31 of 63 nodes with only 3 conflicting nodes (1 strongly conflicting). When only *nt3* characters of fourfold synonymous codons (*nt3_4foldsynon*) are analyzed, thereby largely eliminating the potential for nonsynonymous change, the number of strongly supported nodes drops to 10 out of 63 plus five conflicting nodes (1 strongly conflicting). Even this relatively small level of support is suspect, however, when coupled with information about nucleotide heterogeneity (Figure 8). In particular, the large majority of groups for which there is bootstrap support ≥50% have shared, strongly biased compositions, whether those taxonomic groups are conflicting or not. While this doesn't prove that there isn't a sequence-based signal derived from synonymous change, it demonstrates that compositional heterogeneity is likely to factor into the combined result. Furthermore, all groups supported by synonymous change are restricted to just a few taxa. Clearly, synonymous change is not informative of deeper-level relationships in this study. In this case, a premise that synonymous change ought to be informative and should be retained would be based on the mistaken impression that change at *nt3* is entirely synonymous, when in fact, the change at *nt3* that is informative of deep-level arthropod phylogeny is largely or entirely nonsynonymous. In contrast, the often-implemented *nt12* analyses retain synonymous change at *nt1* and discard useful nonsynonymous information at *nt3*, both of which presumably can result in lowered node support relative to *nt123_degen1* (e.g., see Supp. Figure 2 in [11]; Figure 1 in [5]).

### Analysis of indels

In this study "high-quality" (defined in Materials & Methods) indels that grouped two or more taxa were investigated as a source of phylogenetic information, and 19 were identified (Table 5). Eleven indels support eight groups that are already well supported in sequence-based analyses, e.g., all receive ≥75% bootstrap support from 3 – 42 individual genes. Seven additional indels support seven additional groups that are clearly incorrect. Thus, indels analysis did not prove useful (e.g., see also [85]), although more complex modeling of indel evolution would likely prove useful, as it has for nucleotide and amino acid evolution.

### Conclusion

The current results, plus another to appear shortly (Zwick, Regier, and Zwickl, in preparation), provide additional support and explication of the phylogenetic results presented in Regier et al. (2010) [11]. In particular, there is additional justification for the major emphasis on four analytical approaches, three of which emphasize nonsynonymous change under differing assumptions and a fourth which directly models codon change. This is because synonymous change provides little, if any, useful phylogenetic signal for the deeper nodes, while contributing substantial misinformative signal, perhaps mostly attributable to compositional heterogeneity. The current report clarifies why strong node support for higher-level relationships has been so challenging, namely, that single genes contribute insufficient signal for robust support, necessitating the combined analysis of multiple genes. In fact, the current report suggests that the up-to-approximately 40 kilobase pairs / taxon in the data matrix of Regier et al. (2010) [11], while providing "strong" support for 10 "high-interest" nodes that previously had not been strongly and consistently supported (i.e., Altocrustacea, Communostraca, Hexapoda, Mandibulata, Miracrustacea, Multicrustacea, Oligostraca, Vericrustacea, Xenocarida, Symphyla + Pauropoda), nevertheless proved sensitive to modest reductions in the amount of data. Thus, a reasonable question would be to inquire into the consequence of modest-to-large *increases* in the amount of data. While we detected not even modest support for alternative resolutions of the "high interest" nodes under various data set manipulations, the outcome of increasing the total amount data remains an important experiment to do. Thus, it will be necessary to reinvestigate these relationships with even more data, as will inevitably occur in this new age of relatively cost-efficient phylogenomics.

## Materials and Methods

### Taxon and gene sampling

Taxon and gene sampling are identical to that reported in Regier et al., 2010 [11]. That report includes a complete listing of taxa (75 species Arthropoda, 3 species Onychophora, 2 species Tardigrada) and their higher classification (Supplementary Table 1 in [11]), as well as GenBank numbers for sequences of the 68 gene fragments (Supplementary Table 4 in [11, Table S5 in this report]). In that earlier report [11], as well as [5], one taxon within Pycnogonida (*Colossendeis* sp., lab code name "Col") has now been shown to have been misidentified (see Acknowledgments). It has been reidentified as *Nymphon* sp. (Pycnogonida: Nymphonidae). The lab code name remains "Col". The original misidentification changes nothing as regards conclusions in those earlier publications about the position of Pycnogonida within Arthropoda. In the current report, we use "*Nymphon* sp." and "Nymphonidae" throughout.

### Data sets

All data submatrices were derived from one of two master matrices that include all taxa and all gene fragments, either as is (*nt123*) or after fully degenerating nucleotides (*nt123_degen1*) using IUPAC ambiguity codes at those sites that can potentially undergo synonymous change, thereby making synonymous change largely invisible and reducing the effect of compositional heterogeneity but leaving the inference of nonsynonymous change largely intact (summarized in Table 1). For example, in *nt123_degen1* CAC and CAT (His) are both coded CAY, while TTA, TTG, CTT, CTC, CTA, and CTG (Leu) are all coded YTN. The *nt123_degen1* data matrix and the *nt123* data matrix can both be downloaded as Supplementary Data of Regier et al., 2010 [11]. Software to degenerate sequences is available at http://www.phylotools.com. A character-exclusion mask (2313 characters out of 41,574 total characters) of ambiguously aligned nucleotide characters was also invoked, and is identical to that in Regier et al., 2010 [11]. Using the *degen1* approach, one set of submatrices, called *single-gene matrices*, was derived from each of the 68 gene fragments. Another set consisted of gene fragments grouped by their average rate of nonsynonymous change (according to Table 2 in [5]), such that there were two approximately equal-sized *nt123_degen1* submatrices (*faster* and *slower*; 19842 and 19419 characters, respectively, and corresponding to gene fragment numbers 1–37 and 38–68 in Table 2 of [11]), and another such that there were three approximately equal-sized *nt123_degen1* submatrices (*fastest, medium, slowest*; 13173, 12834, and 13254 characters, respectively, and corresponding to gene fragment numbers 1–24, 25–43, and 44–68 in Table 2 of [11]).

These same collections of gene fragments, namely, 1–37 / 38–68 and 1–24 / 25–43 / 44–68, as well as all genes combined (1–68) were also analyzed in a distinct manner from *degen1* coding but still with the aim of restricting the analysis to nonsynonymous change. Previously, we have called this alternative the *noLRall1nt2* approach, and it is based on character exclusion for *nt123* (non-degenerate) rather than nucleotide degeneration [5] (http://www.phylotools.com). With this approach, two complementary subsets, called *noLRall1nt2* and *LRall1nt3*, are defined as follows: The *noLRall1nt2* character subset consists of all characters at the second codon position (*nt2*) plus only those *nt1* characters that encode no leucine or arginine codons (*noLRall1*). The *LRall1nt3* character subset consists of all characters at the third codon position (*nt3*) plus any and all nt1 characters not present in *noLRall1*. Since leucine and arginine codons are the only ones that undergo synonymous change at *nt1*, their absence from *noLRall1nt2* leads to a dramatic reduction in the amount of synonymous change inferred over a tree. Software to create *noLRall1* and *LRall1* character set definitions is available at http://www.phylotools.com. In this set of analyses, the noLR analysis of gene fragments 1–68 and of the five subsets with differing rates occurred with *noLRall1nt2* only, after excluding *LRall1nt3*.

To explore the informativeness of synonymous change, progressively more restrictive data submatrices were constructed and analyzed with and without degeneration. *LRall1nt3* has already been described, and it was compared with *LRall1nt3_degen1*. Analysis of the latter captures all of the nonsynonymous change in *LRall1nt3* with very little or no synonymous change. Another pair of submatrices was *nt3* and *nt3_degen1*. The final submatrix, called *nt3_4foldsynon*, included only those sites at *nt3* that encode fourfold-degenerate codons, e.g., alanine and glycine; all other sites were converted to *N*. Analysis of the *nt3_4foldsynon* submatrix should capture entirely synonymous change. Degeneration of *nt3_4foldsynon* would yield all *N's* and hence was not performed.

For another set of *degen1* submatrices, character order for the entire *nt123_degen1* data matrix was first randomized using a Perl script (available at http://www.phylotools.com), which randomly resampled all the characters in the matrix without replacement. This reordering (or shuffling) was repeated in series 10,000 times to ensure randomness. Submatrices of differing sizes were constructed (100% as a control, 85%, 50%, 33%, 15%). Only one submatrix each of 100%, 85%, and 15% of the complete size was constructed. For the 50% matrices, five independently randomized 100% matrices were split into five pairs of complementary matrices (ten 50% matrices total). Likewise, two independently randomized 100% matrices were each split into three complementary 33% matrices (six 33% matrices total).

Further, two types of character partitioning were undertaken for total data (*nt123*). In one, the complete data matrix (*nt123*, non-degenerate) was split into two complementary subsets, namely, *noLRall1nt2* and *LRall1nt3*, in order to separate a major source of character variation, namely, nonsynonymous and synonymous change. In the phylogenetic analysis, GTR + G + I models were applied to each subset with unlinked parameters. In another partitioning scheme, the *nt123_degen1* matrix was analyzed by applying GTR + G + I models with unlinked parameters to three subsets described above, namely, *fastest, medium, slowest*.

Insertion / deletion (indel) events were analyzed for phylogenetic informativeness, but only "high-quality" indels, which consisted of those that have relatively unambiguous alignments, defined as those with no staggered ends and identical lengths but not necessarily identical sequences. Before doing this, however, a slightly improved manual alignment relative to that in Regier et al. (2010) [11] was undertaken, this time using a MAFFT alignment ([86]; default values with the BLOSUM62 scoring matrix) of amino acids to guide our decision-making about realignments within the nucleotide data set. Relative to the original alignment [11], there were adjustments in the positioning of sequences for 12 species, 9 of which resulted in realignments of <10 characters, one of 22 characters, one of 63 characters, and one of 93 characters. In addition, we have discovered 592 base pairs of incorrect sequence in the mayfly *Hexagenia limbata* (codename: *May*) that was duplicated in silico from another species' RNA polmerase II (largest subunit) sequence, and this has now been replaced with Ns. The realigned, non-degenerate data matrix used for indel analysis (*nt123*) is included in Supporting Information as *Dataset S1*. Separately, the degenerate, realigned data matrix (*nt123_degen1*; the *degen1* script is available at http://www.phylotools.com) was reanalyzed under likelihood using GARLI 1.0. There were no changes in the maximum likelihood topology relative to Regier et al., 2010 [11], and bootstrap support for all nodes changed by ≤4%.

### Phylogenetic analysis

All phylogenetic analyses are based on the maximum likelihood criterion applied to nucleotides, as implemented in GARLI (v. 0.961, v. 1.0 with and without a character-partitioning feature added; [87]), using the GTR + G + I model. All other parameters are default values. We used the program default settings, including random stepwise addition starting trees, except that we halved the number of successive generation passes yielding no improvement in likelihood score that prompts termination (genthreshfortopoterm  = 10000), as suggested in the GARLI manual. The number of replicate searches used to obtain a maximum likelihood tree estimate for each data set ranged from 550 – 674, following Regier et al. (2009) [66]. The number of pseudo-replicates of the non-parametric bootstrap analyses varied, depending on results being compared and the computational requirements but always matched or exceeded the targeted number of pseudo-replicates. For the data partitioning results, the number of bootstrap replicates is 520–599. For the randomized-sequence comparisons, the number of bootstrap replicates is 470–668. However, in some cases, the computational time was simply too long to perform as large a large number of replicates as for the aforementioned analyses. Thus, rather than keep all analyses at an identical low minimum, we chose instead to accept larger differences between separate analyses for the benefit of additional accuracy for the majority of them. Hence, for the single-gene analyses, the number of bootstrap replicates ranges from 209 to 666. For testing the informativeness of synonymous signal, the number of bootstrap replicates is 651–673, except for the computationally intensive *nt3_4foldsynon* analysis, which was 236. The tree shown in Figure 1 is taken directly from Regier et al. [11], and it is based on 1065 bootstrap replicates. All of these numbers are such that the statistical variation around the mean is still only a few percentage points. Other than the single-gene bootstrap analyses, the number of heuristic search replicates per bootstrap replicate varied from 1–5. For the single-gene bootstrap analyses only, we increased the number of search replicates / bootstrap replicate to 50, except for the longest genes, ef2 (40 search reps) and polii (30 search reps), from 1 previously presented in Supplementary Table 3 of Regier et al. (2010) [11], in order to provide a more accurate estimate for each bootstrap pseudoreplicate. However, the resulting changes in bootstrap percentages were relatively minor (Table S1,S2). Optimal-tree searches and bootstrap analyses were parallelized using Grid computing [88] through The Lattice Project [89]. For purposes of comparison only, we oftentimes collectively refer to nodes with bootstrap values ≥75% in our single-gene studies, and to bootstrap values ≥80% in our multigene studies. In the combined-gene analyses only, we define for heuristic purposes only bootstrap values ≥80% as "strong."

### Estimating phylogenetic utility of individual gene segments

To provide a quantitative estimate for how useful a particular gene segment is for phylogeny reconstruction, an approximate metric called "phylogenetic utility" was developed. Based on single-gene likelihood bootstrap analyses, the total number of taxonomic groups present in Figure 1 that are recovered by the single gene fragment with bootstrap ≥75% is divided by the length (in nt ×10^−3^) of the fragment, and then corrected for missing taxa by multiplying this fraction by (80 taxa / (80 taxa − number of missing taxa)). The calculated value of phylogenetic utility for each segment was plotted relative to its rate of nonsynonymous change (see Table 2 in [5]). Linear regression was performed using *Linest*, a spreadsheet function available in OpenOffice (http://www.openoffice.org). Two features of this metric are worth pointing out. First, it estimates the *total* number of nodes that could be recovered, but in most systematic studies not all nodes are of equal interest. Second, this metric estimates utility as information density (i.e., total information / fragment length), but in most systematic studies what matters in a practical sense is the total amount of information in a sequenceable gene fragment.

### Compositional heterogeneity and synonymous change

To describe compositional heterogeneity in a data matrix, we calculated Euclidean distances on the proportions of the four nucleotide frequencies treated as independent characters using a Perl script (available at http://www.phylotools.com). A separate script available from the same web site was used to restrict the total data matrix to fourfold degenerate codons, which were subsequently split by codon position to produce a matrix (called *nt3_4foldsynon*) of those *nt3* characters that are encoded by fourfold degenerate codons.

## Supporting Information

Table S1 Single-gene bootstrap values (≥75% only, *nt123_degen1*) for taxonomic groups (nodes) present in Figure 1. This table lists bootstrap values for taxa identified in Figure 1 based on analysis of single genes.
(XLS)

Table S2 Single-gene bootstrap values (≥75% only, *nt123_degen1*) for taxonomic groups NOT present in Figure 1. This table lists bootstrap values for taxa not recovered in the analysis shown in Figure 1 based on analysis of single genes.
(XLS)

Table S3 Bootstrap values based on analysis of shuffled data matrices of varying sizes (100% to 15% of complete data matrix). This table lists bootstrap values after randomizing character order in the 100% data matrix and splitting it into portions of varying sizes for analysis (100% to 15% of complete data matrix) without replacement. A subset of the Table-S3 results are also shown in Table 2. This Table-2 subset includes results only for those taxonomic groups that show particularly highly variable bootstrap values between replicates of the 50% matrices.
(DOC)

Table S4 Bootstrap values based on analysis of data sets and subsets differing in their average rates of nonsynonymous change. The complete data set is split into two or three subsets based on average rates of nonsynonymous change of individual genes, and bootstrap analyses are performed to estimate the informativeness of the different rate category ranges. A subset of the Table-S4 results is also shown in Table 3. This Table-3 subset includes results only for those taxonomic groups that show particularly highly variable bootstrap values from differing rate category ranges of gene segments.
(DOC)

Table S5 GenBank accession numbers (also cited in [11]).
(DOC)

Dataset S1 A Nexus-formatted data set that includes nucleotide sequence data (*nt123*) for 80 taxa and 62 genes, slightly realigned relative to that in Regier et al., 2010 [11] (see Materials and Methods, Supplemetary Materials) and used principally for indel analysis. Sets of characters are defined and listed immediately after the data matrix, including those needed to create *noLRall1* and *LRall1*. This data set can be degenerated using the *degen1* script available at http://www.phylotools.com. The species codenames used in this realigned data set are also identified by their complete genus-species names in Table SM1 of Regier et al., 2010 [11].
(NEX)
