# Supplementary material for: Sources of Signal in 62 Protein-Coding Nuclear Genes for Higher-Level Phylogenetics of Arthropods
Source: PLoS One. 2011 Aug 4;6(8):e23408. doi: 10.1371/journal.pone.0023408 (PMC3150433; doi:10.1371/journal.pone.0023408)
Supplement: Table S3 — Bootstrap values based on analysis of shuffled data matrices of varying sizes (100% to 15% of complete data matrix). This table lists bootstrap values after randomizing character order in the 100% data matrix and splitting it into portions of varying sizes for analysis (100% to 15% of complete data matrix) without replacement. A subset of the Table-S3 results are also shown in Table 2. This Table-2 subset includes results only for those taxonomic groups that show particularly highly variable bootstrap values between replicates of the 50% matrices. (DOC) [file pone.0023408.s003.doc]

**Table S3. Bootstrap values based on analysis of reshuffled data matrices of varying sizes (100% to 15% of complete data matrix).^a^**

| taxonomic group | 1-68 gn degen1 (39261 bp) | random100% degen1 (39261 bp) | random85% degen1 (33372 bp) | random50% (#1) degen1 (19630 bp) | random50% (#2) degen1 (19630 bp) | random50% (#3) degen1 (19630 bp) | random50% (#4) degen1 (19630 bp) | random50% (#5) degen1 (19630 bp) | random33% (#1) degen1 (13087 bp) | random33% (#2) degen1 (13087 bp) | random15% degen1 (5889 bp) |
| --- | --- | --- | --- | --- | --- | --- | --- | --- | --- | --- | --- |
| Onychophora | 100 | 100 | 100 | 100 - 100 | 100 - 100 | 100 - 100 | 100 - 100 | 100 - 100 | 100 - 100 - 100 | 100 - 100 - 100 | 100 |
| Peripatopsidae | 100 | 100 | 100 | 100 - 100 | 100 - 100 | 100 - 100 | 100 - 100 | 100 - 100 | 100 - 100 - 100 | 100 - 100 - 100 | 100 |
| Tardigrada | 100 | 100 | 100 | 100 - 100 | 100 - 100 | 100 - 100 | 100 - 100 | 100 - 100 | 100 - 100 - 100 | 100 - 100 - 100 | 100 |
| Arthropoda | 100 | 100 | 100 | 100 - 92 | 99 - 98 | 100 - 97 | 100 - 97 | 100 - 99 | 99 - 80 - 98 | 100 - 69 - 98 | ** 64 |
| Pycnogonida | 100 | 100 | 100 | 100 - 100 | 100 - 100 | 100 - 100 | 100 - 100 | 100 - 100 | 100 - 100 - 100 | 100 - 100 - 100 | 100 |
| 1: Ammotheidae + Endeididae | 93 | 93 | 99 | 89 - 83 | 97 - [<50] | 95 - 70 | [<50] - 96 | 63 - 95 | 90 - <50 - 80 | [<50] - 84 - 88 | ** 74 |
| 2: Nymphonidae + Endeididae | [<50] | [<50] | [<50] | [<50] - [<50] | [<50] - 53 | [<50] - [<50] | 50 - [<50] | [<50] - [<50] | [<50]-[<50]-[<50] | 57 - [<50] - [<50] | [<50] |
| Ammotheidae | 99 | 99 | 87 | 100 - 52 | 72 - 98 | 86 - 97 | 94 - 86 | 89 - 96 | 75 - 68 - 95 | 98 - 69 - [<50] | 99 |
| Tanystylum + Achelia | 98 | 99 | 99 | 58 - 100 | 90 - 96 | 95 - 83 | [<50] - 100 | 86 - 96 | 98 - 86 - 50 | 76 - 90 - 82 | ** [<50] |
| 1: Chelicerata | 74 | 73 | 74 | 54 - 78 | 62 - 72 | 69 - 73 | 67 - 62 | [<50] - 90 | [<50] - [<50] - 80 | [<50] - 76 - 72 | 52 |
| 2: Arthropoda minus Pycnogonida | [<50] | [<50] | [<50] | [<50] - [<50] | [<50] - [<50] | [<50] - [<50] | [<50] - [<50] | 77 - [<50] | 70 - [<50] - [<50] | 55 - [<50] - [<50] | [<50] |
| Euchelicerata | 100 | 100 | 100 | 100 - 100 | 100 - 100 | 100 - 100 | 100 - 100 | 100 - 100 | 100 - 100 - 100 | 100 - 100 - 100 | 100 |
| Xiphosura | 100 | 100 | 100 | 100 - 100 | 100 - 100 | 100 - 100 | 100 - 100 | 100 - 100 | 100 - 100 - 100 | 100 - 100 - 100 | 100 |
| Arachnida | 68 | 66 | 67 | <50 - <50 | 60 - [<50] | 70 - 51 | 77 - [<50] | [<50] - 62 | [<50] - <50 - 82 | 60 - [<50] - 53 | <50 |
| Pulmonata | 65 | 68 | 70 | 51 - 89 | [<50] - 89 | 64 - 86 | 64 - [54] | [<50] - 96 | 70 - 73 - 61 | 75 - [<50] - 89 | 59 |
| Scorpiones | 100 | 100 | 100 | 100 - 100 | 100 - 100 | 100 - 100 | 100 - 100 | 100 - 100 | 100 - 100 - 100 | 100 - 100 - 100 | 100 |
| Tetrapulmonata | 99 | 100 | 94 | 100 - [<50] | 100 - 59 | 99 - 63 | 91 - 95 | 91 - 96 | 89 - 64 - 97 | 51 - 98 - 88 | 88 |
| Pedipalpi | 100 | 100 | 100 | 97 - 98 | 98 - 98 | 95 - 100 | 99 - 98 | 97 - 100 | 99 - 98 - 89 | 88 - 92 - 100 | ** 50 |
| Uropygi | 100 | 100 | 100 | 99 - 96 | 97 - 99 | 98 - 94 | 87 - 100 | 100 - 71 | 64 - 98 - 98 | 98 - 88 - 100 | ** 58 |
| Mandibulata | 99 | 99 | 100 | 97 - 90 | 96 - 94 | 84 - 98 | 99 - 76 | 99 - 83 | 96 - 66 - 63 | 99 - 66 - 96 | ** 60 |
| Myriapoda | 100 | 100 | 100 | 100 - 96 | 100 - 94 | 100 - 99 | 98 - 100 | 100 - 98 | 94 - 94 - 100 | 93 - 99 - 98 | 97 |
| Chilopoda | 100 | 100 | 100 | 100 - 100 | 100 - 100 | 100 - 100 | 100 - 100 | 100 - 100 | 100 - 100 - 100 | 100 - 100 - 100 | 100 |
| Pleurostigmophora | 93 | 92 | 92 | 91 - 59 | 82 - 82 | 79 - 82 | 77 - 85 | 92 - 67 | [<50] - 78 - 86 | <50 - 89 - 77 | ** <50 |
| Scolopendromorpha + Lithobiomorpha | 99 | 99 | 95 | [<50] - 100 | 87 - 99 | 78 - 100 | 96 - 95 | 100 - 82 | 98 - 75 - 88 | 98 - 93 - [<50] | 98 |
| 1: Progoneata | 67 | 69 | 57 | 51 - <50 | 61 - [<50] | 70 - [<50] | 66 - <50 | <50 - 56 | [<50] - 55 - <50 | [<50] - <50 - [<50] | [<50] |
| 2. Chilopoda + Diplopoda | [<50] | [<50] | [<50] | [<50] - [<50] | [<50] - 65 | [<50] - 67 | [<50] - [<50] | [<50] - [<50] | [<50]-[<50]-[<50] | <50 - [<50] - <50 | [<50] |
| Diplopoda | 99 | 100 | 99 | 50 - 100 | 78 - 100 | 74 - 99 | 77 - 99 | 95 - 95 | 72 - 99 - 64 | 78 - 91 - 91 | ** [<50] |
| Chilognatha | 100 | 100 | 100 | 100 - 100 | 100 - 100 | 100 - 100 | 100 - 100 | 100 - 100 | 100 - 100 - 100 | 100 - 100 - 100 | 100 |
| 1: Callipodida + Polyzoniida | 55 | 55 | 50 | [<50] - [<50] | [<50] - [<50] | [<50] - [<50] | <50 - 53 | 86 - [<50] | [<50] - <50 - 62 | [<50] - [<50]- 75 | [<50] |
| 2: Spirobolida + Polyzoniida | [<50] | [<50] | [<50] | 54 - 50 | [<50] - 57 | 57 - [<50] | [<50] - [<50] | [<50] - 57 | <50 - [<50]-[<50] | 62 - [<50] - [<50] | [<50] |
| 3.: Callipodida + Spirobolida | [<50] | [<50] | [<50] | [<50] - [<50] | 52 - [<50] | [<50] - <50 | [<50] - [<50] | [<50] - [<50] | [<50]-[<50]-[<50] | [<50] - 72 - [<50] | 85 |
| Symphyla + Pauropoda | 92 | 94 | 85 | <50 - 73 | 50 - 84 | 56 - 83 | 70 - 64 | <50 - 90 | [<50] - 84 - <50 | 63 - [<50] - 81 | ** [<50] |
| Symphyla | 100 | 100 | 100 | 100 - 100 | 100 - 100 | 100 - 100 | 100 - 100 | 100 - 100 | 100 - 100 - 100 | 100 - 100 - 100 | 100 |
| Pancrustacea | 100 | 100 | 100 | 100 - 100 | 100 - 100 | 100 - 100 | 100 - 100 | 100 - 100 | 100 - 100 - 100 | 100 - 100 - 100 | 100 |
| Oligostraca | 100 | 100 | 95 | 95 - 87 | 99 - 55 | 95 - 85 | 84 - 80 | 84 - 90 | 88 - 82 - 74 | 92 - 58 - 69 | ** <50 |
| Ostracoda | 60 | 59 | 62 | <50 - 58 | [<50] - <50 | <50 - <50 | <50 - 74 | 78 - [<50] | [<50]-[<50]-[<50] | [<50] - [<50] - 75 | [<50] |
| Myodocopa | 100 | 100 | 100 | 100 - 100 | 100 - 100 | 100 - 100 | 100 - 100 | 100 - 100 | 100 - 100 - 100 | 100 - 100 - 100 | 100 |
| Ichthyostraca + Mystacocarida | <50 | <50 | [<50] | [<50] - <50 | [<50] - <50 | [<50] - <50 | [<50] - <50 | [<50] - [<50] | [<50]-[<50]-[<50] | [<50]-[<50]-[<50] | [<50] |
| Ichthyostraca | 100 | 100 | 100 | 100 - 100 | 100 - 100 | 100 - 100 | 100 - 100 | 100 - 100 | 100 - 100 - 100 | 100 - 100 - 100 | 99 |
| Altocrustacea | 93 | 93 | 89 | 68 - 75 | 87 - <50 | 95 - [<50] | <50 - 93 | 60 - 77 | 82 - <50 - [<50] | 54 - <50 - 77 | ** <50 |
| Vericrustacea | 86 | 87 | 64 | <50 - 71 | 74 - <50 | 77 - [<50] | <50 - 77 | 58 - 56 | [<50]- <50 -[<50] | [<50] - <50 - 70 | ** <50 |
| Branchiopoda | 100 | 100 | 100 | 100 - 100 | 100 - 100 | 100 - 100 | 100 - 100 | 100 - 100 | 100 - 100 - 100 | 100 - 100 - 100 | 89 |
| Anostraca | 100 | 100 | 100 | 100 - 100 | 100 - 100 | 100 - 100 | 100 - 100 | 100 - 100 | 100 - 100 - 100 | 100 - 100 - 100 | 100 |
| Phyllopoda | 100 | 100 | 100 | 100 - 100 | 100 - 100 | 100 - 100 | 100 - 100 | 100 - 100 | 100 - 100 - 100 | 100 - 100 - 100 | 98 |
| Diplostraca | 100 | 100 | 100 | 100 - 100 | 98 - 100 | 100 - 100 | 100 - 100 | 100 - 100 | 99 - 99 - 96 | 97 - 100 - 99 | 93 |
| Cladocera + Spinicaudata | 100 | 100 | 100 | 100 - 100 | 100 - 100 | 100 - 100 | 100 - 100 | 100 - 100 | 100 - 100 - 100 | 100 - 100 - 100 | 99 |
| Multicrustacea | 100 | 100 | 100 | 100 - 71 | 83 - 99 | 99 - 96 | 97 - 99 | 96 - 95 | 85 - 90 - [<50] | 89 - 71 - 95 | ** <50 |
| Copepoda | 100 | 100 | 100 | 100 - 100 | 100 - 100 | 100 - 100 | 100 - 100 | 100 - 100 | 100 - 100 - 100 | 100 - 100 - 100 | 100 |
| Cyclopoida | 100 | 100 | 100 | 100 - 100 | 100 - 100 | 100 - 100 | 100 - 100 | 100 - 100 | 100 - 100 - 100 | 100 - 100 - 100 | 100 |
| Communostraca | 84 | 83 | 84 | <50 - 87 | [<50] - 95 | 88 - [<50] | 54 - 82 | <50 - 87 | 59 - 84 - 69 | 72 - 54 - 55 | ** 54 |
| Malacostraca | 100 | 100 | 100 | 100 - 100 | 100 - 100 | 100 - 100 | 100 - 100 | 100 - 100 | 100 - 100 - 100 | 100 - 100 - 100 | 100 |
| Eumalacostraca | 100 | 100 | 100 | 100 - 99 | 100 - 100 | 100 - 100 | 100 - 100 | 100 - 100 | 99 - 99 - 97 | 98 - 100 - 100 | ** 67 |
| Eucarida + Peracarida | 87 | 86 | 89 | 90 - <50 | 62 - 94 | 58 - 69 | 78 - 68 | 63 - 86 | 94 - [<50] - <50 | <50 - 77 - 63 | ** [<50] |
| Thecostraca | 100 | 100 | 100 | 100 - 100 | 100 - 100 | 100 - 100 | 100 - 100 | 100 - 100 | 100 - 100 - 100 | 100 - 100 - 100 | 100 |
| Thoracica | 100 | 100 | 100 | 100 - 100 | 100 - 100 | 100 - 100 | 100 - 100 | 100 - 100 | 100 - 100 - 100 | 100 - 100 - 100 | 87 |
| Sessilia | 97 | 97 | 95 | 82 - 94 | 83 - 93 | 99 - 66 | 81 - 93 | 85 - 86 | 87 - 72 - 86 | 92 - 69 - 99 | ** <50 |
| Miracrustacea | 94 | 95 | 93 | 70 - 58 | 79 - <50 | 94 - [<50] | 73 - 80 | <50 - 92 | 78 - 51 - [<50] | 58 - 59 - 79 | ** [<50] |
| Xenocarida | 93 | 94 | 94 | 75 - 78 | 100 - [<50] | 94 - <50 | 77 - 80 | 52 - 91 | 91 - [<50] - [<50] | 73 - <50 - 79 | ** [<50] |
| Hexapoda | 100 | 100 | 99 | 88 - 100 | 100 - 89 | 99 - 99 | 94 - 100 | 99 - 97 | 96 - 94 - 95 | 96 - 90 - 97 | 95 |
| Entognatha | 86 | 84 | 69 | 74 - 73 | 74 - 83 | 63 - 91 | 88 - 65 | [51] - 91 | 78 - 87 - [<50] | [51] - 87 - 59 | ** [53] |
| Diplura | 100 | 100 | 100 | 100 - 100 | 100 - 100 | 100 - 100 | 100 - 100 | 100 - 100 | 100 - 100 - 100 | 100 - 100 - 100 | 95 |
| Collembola | 100 | 100 | 100 | 100 - 100 | 100 - 100 | 100 - 100 | 100 - 100 | 100 - 100 | 100 - 100 - 100 | 100 - 100 - 100 | 100 |
| Entomobryomorpha | 98 | 97 | 94 | 88 - 93 | 99 - 67 | 96 - 82 | 69 - 99 | 93 - 91 | 82 - 62 - 98 | 98 - 66 - 83 | 91 |
| Insecta | 100 | 100 | 100 | 100 - 100 | 100 - 100 | 100 - 100 | 100 - 100 | 100 - 100 | 100 - 100 - 98 | 100 - 100 - 100 | 99 |
| Archaeognatha | 100 | 100 | 100 | 100 - 100 | 100 - 100 | 100 -100 | 100 - 100 | 100 - 100 | 100 - 100 - 100 | 100 - 100 - 100 | 100 |
| Dicondylia | 100 | 100 | 100 | 99 - 98 | 98 - 100 | 100 - 97 | 98 - 100 | 97 - 98 | 100 - 92 - 84 | 97 - 81 - 98 | 88 |
| Zygentoma | 100 | 100 | 100 | 100 - 100 | 100 - 100 | 100 - 100 | 100 - 100 | 100 - 100 | 100 - 100 - 100 | 96 - 100 - 100 | 96 |
| Pterygota | 99 | 99 | 98 | 77 - 95 | 93 - 79 | 96 - 85 | 50 - 89 | 82 - 67 | 64 - 84 - 51 | <50 - 64 - 91 | ** <50 |
| Paleoptera | 69 | 68 | 59 | [<50] - 55 | [<50] - 79 | [<50] - 70 | [<50] - 70 | [<50] - 67 | <50 - <50 - 54 | 64 - [<50] - [<50] | <50 |
| Ephemeroptera | 100 | 100 | 100 | 100 - 100 | 100 - 100 | 100 - 100 | 100 - 100 | 100 - 100 | 100 - 100 - 100 | 100 - 100 - 100 | 100 |
| Odonata | 100 | 100 | 100 | 100 - 100 | 100 - 100 | 100 - 100 | 100 - 100 | 100 - 100 | 100 - 100 - 100 | 100 - 100 - 100 | 100 |
| Neoptera | 97 | 97 | 97 | 73 - 91 | 84 - 75 | 75 - 89 | 92 - 73 | 98 - <50 | <50 - 96 - 53 | <50 - 91 - [<50] | ** <50 |
| Polyneoptera | 99 | 100 | 100 | 91 - 99 | 100 - 95 | 77 - 100 | 91 - 99 | 100 - 92 | 100 - 95 - 70 | 100 - 97 - 69 | ** [<50] |
| Blattodea + Orthoptera | 94 | 93 | 89 | 86 - 84 | 59 - 98 | 86 - 84 | 99 - [<50] | 99 - [57] | 75 - 97 - [<50] | [<50] - 82 - 95 | 92 |
| Lepidoptera | 100 | 100 | 100 | 100 - 100 | 100 - 100 | 100 - 100 | 100 - 100 | 100 - 100 | 100 - 100 - 100 | 100 - 100 - 100 | 100 |
| Ditrysia | 100 | 100 | 100 | 100 - 100 | 100 - 100 | 100 - 100 | 100 - 100 | 100 - 100 | 100 - 100 - 100 | 100 - 100 - 100 | 100 |

^a^ In column 2 are shown the results for the complete matrix, which match those in Figure 1. All columns to the right of column 2 contain bootstrap values from analyses of differing amounts (100% - 15%) of randomized data drawn from the complete data set. The 50% data were separately sampled in complementary pairs from fives separately randomized entire matrices (#1 - #5), and complementary halves were analyzed and are shown in individual columns. The 33% data were generated from two separately randomized entire matrices, and the complementary thirds were analyzed. In the 15% analysis, results for taxa whose bootstrap values are reduced by ≥10% relative to the complete data matrix (column 2) are preceded by double asterisks (**). Taxonomic groups not present in the maximum-likelihood topology for that analysis are bracketed. Alternatives for four taxonomic groups shown in Figure 1 are also displayed.
