# Supplementary material for: Sources of Signal in 62 Protein-Coding Nuclear Genes for Higher-Level Phylogenetics of Arthropods
Source: PLoS One. 2011 Aug 4;6(8):e23408. doi: 10.1371/journal.pone.0023408 (PMC3150433; doi:10.1371/journal.pone.0023408)
Supplement: Table S4 — Bootstrap values based on analysis of data sets and subsets differing in their average rates of nonsynonymous change. The complete data set is split into two or three subsets based on average rates of nonsynonymous change of individual genes, and bootstrap analyses are performed to estimate the informativeness of the different rate category ranges. A subset of the Table-S4 results is also shown in Table 3. This Table-3 subset includes results only for those taxonomic groups that show particularly highly variable bootstrap values from differing rate category ranges of gene segments. (DOC) [file pone.0023408.s004.doc]

**Table S4. Bootstrap values based on analysis of data sets and subsets differing in their average rates of nonsynonymous change.^a^**

| taxonomic group | 1-68 gn, degen1, 39261 bp | 1-37 gn, degen1, 19842 bp | 38-68 gn, degen1, 19419 bp | 1-24 gn, degen1, 13173 bp | 25-43 gn, degen1, 12834 bp | 44-68 gn, degen1, 13254 bp | 1-37 / 38-68 gn, degen1, 39261 bp | 1-24 / 25-43 / 44-68 gn, degen1, 39261 bp | 1-68 gn, nt123, 39261 bp | 1-68 gn, noLRall1+ nt2 / LRall1nt3, 39261 bp | 1-68 gn, noLRall1+ nt2, 21823 bp | 1-37 gn, noLRall1+ nt2, 10618 bp | 38-68 gn, noLRall1+ nt2, 11205 nt | 1-24 gn, noLRall1+ nt2, 6968 bp | 25-43 gn, noLRall1+ nt2, 7161 bp | 44-68 gn, noLRall1+ nt2, 7694 bp |
| --- | --- | --- | --- | --- | --- | --- | --- | --- | --- | --- | --- | --- | --- | --- | --- | --- |
| Onychophora | 100 | 100 | 100 | 100 | 100 | 100 | 100 | 100 | 100 | 100 | 100 | 100 | 100 | 100 | 100 | 100 |
| Peripatopsidae | 100 | 100 | 100 | 100 | 100 | 100 | 100 | 100 | 100 | 100 | 100 | 100 | 100 | 100 | 100 | 100 |
| Tardigrada | 100 | 100 | 100 | 100 | 100 | 100 | 100 | 100 | 100 | 100 | 100 | 100 | 100 | 100 | 100 | 100 |
| Arthropoda | 100 | 81 | 100 | 65 | 93 | 100 | 100 | 100 | ** 63 | ** 87 | 100 | 82 | 100 | 74 | 77 | 100 |
| Pycnogonida | 100 | 100 | 100 | 100 | 100 | 100 | 100 | 100 | 100 | 100 | 100 | 100 | 100 | 100 | 100 | 100 |
| 1: Ammotheidae + Endeididae | 93 | 91 | 57 | 99 | 86 | [<50] | 93 | 92 | ** 62 | 84 | ** 76 | 74 | 63 | 97 | 78 | [<50] |
| 2: Nymphonidae + Endeididae | [<50] | [<50] | [<50] | [<50] | [<50] | 82 | [<50] | [<50] | [<50] | [<50] | [<50] | [<50] | [<50] | [<50] | [<50] | 90 |
| Ammotheidae | 99 | 79 | 95 | 87 | [<50] | 60 | 100 | 92 | 98 | 99 | 99 | 71 | 97 | 76 | [<50] | 75 |
| Tanystylum + Achelia | 98 | 97 | 68 | 99 | 81 | [<50] | 98 | 99 | ** 87 | 95 | 99 | 99 | 88 | 100 | 93 | [<50] |
| 1: Chelicerata | 74 | 73 | 55 | 53 | 96 | [<50] | 76 | 74 | ** [<50] | ** [<50] | ** [<50] | 58 | [<50] | 53 | [<50] | [<50] |
| 2: Arthropoda minus Pycnogonida | [<50] | [<50] | [<50] | [<50] | [<50] | 91 | [<50] | [<50] | 60 ** | 76 ** | <50 | [<50] | 60 | [<50] | [<50] | 88 |
| Euchelicerata | 100 | 100 | 100 | 100 | 100 | 100 | 100 | 100 | 100 | 100 | 100 | 100 | 100 | 100 | 100 | 100 |
| Xiphosura | 100 | 100 | 100 | 100 | 100 | 100 | 100 | 100 | 100 | 100 | 100 | 100 | 100 | 100 | 100 | 100 |
| Arachnida | 68 | [<50] | 89 | [<50] | [<50] | 83 | 72 | 71 | ** [<50] | ** [<50] | 80 ** | [<50] | 71 | [<50] | <50 | 62 |
| Pulmonata | 65 | [<50] | 93 | 56 | 57 | 94 | 75 | 74 | 100 | 100 ** | 75 | [<50] | 92 | 55 | 68 | 90 |
| Scorpiones | 100 | 100 | 100 | 100 | 100 | 100 | 100 | 100 | 100 | 100 | 100 | 100 | 100 | 100 | 100 | 100 |
| Tetrapulmonata | 99 | 99 | 67 | 92 | 76 | 67 | 99 | 100 | ** 71 | 91 | 97 | 96 | 66 | 86 | <50 | 60 |
| Pedipalpi | 100 | 100 | 97 | 99 | 90 | 88 | 100 | 100 | 100 | 100 | ** 85 | 78 | 76 | 71 | 68 | 67 |
| Uropygi | 100 | 99 | 97 | 99 | 91 | 75 | 100 | 100 | 100 | 100 | 98 | 94 | 91 | 90 | 77 | 88 |
| Mandibulata | 99 | 87 | 91 | 70 | 69 | 88 | 99 | 99 | ** 63 | ** 87 | 99 | 76 | 98 | 67 | 68 | 93 |
| Myriapoda | 100 | 96 | 100 | 98 | 99 | 94 | 100 | 100 | 93 | 99 | 100 | 90 | 99 | 94 | 98 | 86 |
| Chilopoda | 100 | 100 | 100 | 100 | 100 | 100 | 100 | 100 | 100 | 100 | 100 | 100 | 100 | 100 | 100 | 100 |
| Pleurostigmophora | 93 | 94 | <50 | 95 | [<50] | [<50] | 93 | 93 | ** [<50] | ** [<50] | ** 59 | 65 | [<50] | 85 | [<50] | [<50] |
| Scolopendromorpha + Lithobiomorpha | 99 | 99 | 88 | 89 | 95 | 52 | 99 | 99 | ** [<50] | ** 64 | 99 | 94 | 89 | 63 | 99 | 58 |
| 1: Progoneata | 67 | 64 | [<50] | 74 | [<50] | [<50] | 59 | 58 | ** [<50] | ** [<50] | 72 | 56 | 77 | 72 | [<50] | [<50] |
| 2. Chilopoda + Diplopoda | [<50] | [<50] | 58 | [<50] | <50 | 79 | [<50] | [<50] | 66 ** | ** 74 | [<50] | [<50] | [<50] | [<50] | [<50] | 67 |
| Diplopoda | 99 | 98 | 83 | 95 | [<50] | 97 | 99 | 99 | 99 | 100 | 95 | 86 | 77 | 80 | [<50] | 94 |
| Chilognatha | 100 | 100 | 100 | 100 | 100 | 100 | 100 | 100 | 100 | 100 | 100 | 100 | 100 | 100 | 100 | 100 |
| 1: Callipodida + Polyzoniida | 55 | [<50] | 72 | [<50] | 99 | [<50] | 53 | 54 | [<50] | [<50] | 71 ** | 54 | 72 | 52 | 98 | [<50] |
| 2: Spirobolida + Polyzoniida | [<50] | [<50] | [<50] | [<50] | [<50] | 93 | [<50] | [<50] | 59 | 70 ** | [<50] | [<50] | [<50] | [<50] | [<50] | 79 |
| 3.: Callipodida + Spirobolida | [<50] | <50 | [<50] | 55 | [<50] | [<50] | [<50] | [<50] | [<50] | [<50] | [<50] | [<50] | [<50] | [<50] | [<50] | [<50] |
| Symphyla + Pauropoda | 92 | 59 | 78 | 65 | [<50] | 88 | 93 | 92 | 99 | 100 | 92 | 58 | 75 | 63 | [<50] | 78 |
| Symphyla | 100 | 100 | 100 | 100 | 100 | 100 | 100 | 100 | 100 | 100 | 100 | 100 | 100 | 100 | 100 | 100 |
| Pancrustacea | 100 | 100 | 100 | 94 | 100 | 100 | 100 | 100 | 100 | 100 | 99 | 100 | 100 | 97 | 100 | 100 |
| Oligostraca | 100 | 89 | 93 | 77 | 91 | 65 | 99 | 99 | ** [<50] | ** [<50] | 95 | 77 | 62 | 58 | 61 | <50 |
| Ostracoda | 60 | [<50] | 85 | [<50] | [<50] | 95 | 53 | 50 | ** [<50] | ** [<50] | 78 ** | [<50] | 85 | <50 | [<50] | 92 |
| Myodocopa | 100 | 100 | 100 | 100 | 100 | 100 | 100 | 100 | 100 | 100 | 100 | 100 | 100 | 99 | 100 | 100 |
| Ichthyostraca + Mystacocarida | <50 | [<50] | 56 | [<50] | [<50] | 76 | <50 | <50 | [<50] | [<50] | <50 | [<50] | <50 | [<50] | [<50] | 69 |
| Ichthyostraca | 100 | 100 | 100 | 100 | 100 | 98 | 100 | 100 | 100 | 100 | 100 | 100 | 100 | 97 | 100 | 98 |
| Altocrustacea | 93 | <50 | 94 | [<50] | <50 | 88 | 94 | 93 | ** [<50] | ** [<50] | 89 | [<50] | 88 | [<50] | <50 | 77 |
| Vericrustacea | 86 | <50 | 67 | [<50] | [<50] | 67 | 89 | 88 | ** [<50] | ** [<50] | ** 88 | <50 | 77 | <50 | <50 | 64 |
| Branchiopoda | 100 | 100 | 100 | 99 | 100 | 100 | 100 | 100 | 100 | 100 | 100 | 100 | 100 | 100 | 100 | 99 |
| Anostraca | 100 | 100 | 100 | 100 | 100 | 100 | 100 | 100 | 100 | 100 | 100 | 100 | 100 | 100 | 100 | 100 |
| Phyllopoda | 100 | 100 | 100 | 100 | 61 | 100 | 100 | 100 | 100 | 100 | 100 | 100 | 99 | 100 | 66 | 100 |
| Diplostraca | 100 | 100 | 100 | 100 | 99 | 93 | 100 | 100 | 96 | 99 | 100 | 100 | 100 | 100 | 92 | 96 |
| Cladocera + Spinicaudata | 100 | 100 | 100 | 100 | 99 | 99 | 100 | 100 | 100 | 100 | 100 | 100 | 100 | 100 | 99 | 98 |
| Multicrustacea | 100 | <50 | 100 | 65 | <50 | 98 | 100 | 100 | ** [<50] | ** 51 | 100 | <50 | 99 | <50 | [<50] | 99 |
| Copepoda | 100 | 100 | 100 | 100 | 100 | 100 | 100 | 100 | 100 | 100 | 100 | 100 | 100 | 100 | 100 | 100 |
| Cyclopoida | 100 | 100 | 100 | 100 | 100 | 100 | 100 | 100 | 100 | 100 | 100 | 100 | 100 | 100 | 100 | 100 |
| Communostraca | 84 | [<50] | 94 | [<50] | [<50] | 95 | 87 | 88 | ** [<50] | ** <50 | ** 57 | [<50] | 88 | [<50] | <50 | 90 |
| Malacostraca | 100 | 100 | 100 | 100 | 100 | 100 | 100 | 100 | 100 | 100 | 100 | 100 | 100 | 100 | 100 | 100 |
| Eumalacostraca | 100 | 100 | 100 | 100 | 86 | 99 | 100 | 100 | 100 | 100 | 100 | 100 | 96 | 98 | 64 | 99 |
| Eucarida + Peracarida | 87 | 61 | 68 | 79 | 53 | 77 | 81 | 83 | ** [<50] | ** [<50] | ** 51 | 55 | 52 | 55 | [<50] | <50 |
| Thecostraca | 100 | 100 | 100 | 100 | 100 | 100 | 100 | 100 | 100 | 100 | 100 | 100 | 100 | 100 | 100 | 100 |
| Thoracica | 100 | 100 | 100 | 100 | 100 | 100 | 100 | 100 | 100 | 100 | 100 | 100 | 100 | 100 | 100 | 100 |
| Sessilia | 97 | 93 | 82 | 97 | [<50] | 87 | 96 | 95 | 100 | 99 | 99 | 98 | 78 | 100 | [<50] | 85 |
| Miracrustacea | 94 | [<50] | 98 | [<50] | <50 | 91 | 97 | 96 | ** [<50] | ** [<50] | 98 | <50 | 98 | [<50] | <50 | 90 |
| Xenocarida | 93 | [<50] | 98 | [<50] | 76 | 94 | 96 | 94 | ** [<50] | ** [<50] | 100 | 67 | 99 | <50 | 81 | 95 |
| Hexapoda | 100 | 64 | 100 | <50 | 100 | 100 | 100 | 100 | ** [<50] | ** [<50] | 99 | [<50] | 100 | [<50] | 95 | 96 |
| Entognatha | 86 | <50 | 88 | <50 | [<50] | 91 | 84 | 79 | ** [<50] | ** [<50] | 89 | [<50] | 88 | [<50] | [<50] | 93 |
| Diplura | 100 | 100 | 100 | 98 | 100 | 95 | 100 | 100 | ** [<50] | ** [<50] | 100 | 100 | 96 | 95 | 100 | 74 |
| Collembola | 100 | 100 | 100 | 100 | 100 | 100 | 100 | 100 | 100 | 100 | 100 | 100 | 100 | 100 | 100 | 100 |
| Entomobryomorpha | 98 | 99 | [<50] | 93 | 90 | [<50] | 97 | 97 | ** 74 | 88 | 95 | 98 | [<50] | 91 | 94 | [<50] |
| Insecta | 100 | 100 | 100 | 100 | 100 | 100 | 100 | 100 | ** [<50] | ** [<50] | 100 | 100 | 100 | 100 | 100 | 100 |
| Archaeognatha | 100 | 100 | 100 | 100 | 100 | 100 | 100 | 100 | ** [<50] | ** [<50] | 100 | 100 | 100 | 100 | 100 | 100 |
| Dicondylia | 100 | 97 | 99 | 65 | 99 | 98 | 100 | 100 | ** [<50] | ** [<50] | 99 | 97 | 86 | 61 | 97 | 75 |
| Zygentoma | 100 | 100 | 100 | 100 | 97 | 100 | 100 | 100 | ** [<50] | ** [<50] | 100 | 100 | 100 | 98 | 99 | 99 |
| Pterygota | 99 | 96 | 71 | 80 | 80 | <50 | 99 | 98 | ** [<50] | ** [<50] | 99 | 98 | 59 | 81 | 86 | [<50] |
| Paleoptera | 69 | [<50] | <50 | [<50] | 54 | [<50] | 71 | 73 | ** [52] | ** <50 | 59 | <50 | <50 | [<50] | 66 | [<50] |
| Ephemeroptera | 100 | 100 | 100 | 100 | 100 | 100 | 100 | 100 | 100 | 100 | 100 | 100 | 100 | 100 | 100 | 100 |
| Odonata | 100 | 100 | 100 | 100 | 100 | 100 | 100 | 100 | 100 | 100 | 100 | 100 | 100 | 100 | 100 | 100 |
| Neoptera | 97 | 77 | 96 | [<50] | 91 | 78 | 98 | 98 | ** [<50] | ** [<50] | 96 | 78 | 95 | [<50] | 94 | 68 |
| Polyneoptera | 99 | 100 | [<50] | 100 | 96 | [<50] | 99 | 100 | 100 | 100 | 94 | 99 | [<50] | 98 | [<50] | [<50] |
| Blattodea + Orthoptera | 94 | 76 | 94 | 86 | [<50] | 86 | 96 | 95 | ** [<50] | ** [<50] | 99 | 81 | 92 | 91 | [<50] | 82 |
| Lepidoptera | 100 | 100 | 100 | 100 | 100 | 100 | 100 | 100 | 100 | 100 | 100 | 100 | 100 | 100 | 100 | 100 |
| Ditrysia | 100 | 100 | 100 | 100 | 98 | 100 | 100 | 100 | 100 | 100 | 100 | 100 | 100 | 100 | 99 | 99 |

^a^ The different data matrices analyzed are listed in the top row using abbreviations. "1-68 gn" refers to all 68 gene segments, whereas "1-37 gn" refers to characters in the faster half, etc.. The sizes of the data set are shown in basepairs (bp). Data sets that are derived from nt123_degen1 are listed as "degen1". noLRall1nt2 and LRall1nt3 are derived from nt123 (non-degenerate) and largely separate nonsynonymous and synonymous change, respectively, as described in Materials & Methods. Data subsets separated by forward slash (/), such as "1-37 / 38-68" refer to partitioned analyses. In three columns, the "**" refer to BP values that vary by >10% from those in "1-68 gn, degen1, 39261 bp". When the BP values are lower, the "**" are to the left of the number. When higher, to the right.
