# Supplementary material for: Sources of Signal in 62 Protein-Coding Nuclear Genes for Higher-Level Phylogenetics of Arthropods
Source: PLoS One. 2011 Aug 4;6(8):e23408. doi: 10.1371/journal.pone.0023408 (PMC3150433; doi:10.1371/journal.pone.0023408)
Supplement: Table S5 — GenBank accession numbers (also cited in [11]). (DOC) [file pone.0023408.s005.doc]

**Table S5. GenBank accession numbers.**^a^

2fin3_4:

A369COPE GQ886751

AarPENTA GQ886752

AchARACH GQ886753

AdoNEOPT GQ886754

AeliPYCNO GQ886755

AhiPYCNO GQ886756

AmaDIPLO GQ886757

Amb2ARACH GQ886758

Arg2BIURA GQ886759

Asa3BRANCH GQ886760

Avu3MALA GQ886761

BbaTHECOS GQ886762

CfrTHECOS GQ886763

CliZYGEN GQ886764

Col2PYCNO GQ886765

Cro2XIPHOS GQ886766

Crp2ARACH GQ886767

Ctas2CHILO GQ886768

Din2ARACH GQ886769

DmaBRANCH GQ886770

DtyMYSTACO GQ886771

EafCOPE GQ886772

EfrDIPLUR GQ886773

EgigARACH GQ886774

EinEPHEM GQ886775

ElePYCNO GQ886776

ErwONYCH GQ886777

EuryPAURO GQ886778

Han2SYMPH GQ886779

HapaOST GQ886780

HariARACH GQ886781

HmaCEPHAL GQ886782

HspARACH GQ886783

IpumARACH GQ886784

IveODONAT GQ886785

JapDIPLUR GQ886786

LeanTHECOS GQ886787

LemMALA GQ886788

Lle2BRANCH GQ886789

LlyODONAT GQ886790

LnigARACH GQ886791

LoxTHECOS GQ886792

LynBRANCH GQ886793

MbaARCHEO GQ886794

MtdTARD GQ886795

NeoMALA GQ886796

NmeZYGEN GQ886797

OimCOLL GQ886798

PamNEOPT GQ886799

PepONYCH GQ886800

Pge2DIPLO GQ886801

Pma2ARACH GQ886802

Pno2ONYCH GQ886803

Pol2DIPLO GQ886804

PsaARCHEO GQ886805

PwhARACH GQ886806

ScolCHILO GQ886807

Scu3SYMPH GQ886808

SkleOST GQ886809

SpoCHILO GQ886810

StpARACH GQ886811

Tom2COLL GQ886812

UfsBRANCH GQ886813

2fin7_8:

AarPENTA GQ886814

AchARACH GQ886815

AdoNEOPT GQ886816

AhiPYCNO GQ886817

AmaDIPLO GQ886818

Amb2ARACH GQ886819

Arg2BIURA GQ886820

Asa3BRANCH GQ886821

Avu3MALA GQ886822

BbaTHECOS GQ886823

CliZYGEN GQ886824

Crp2ARACH GQ886825

Ctas2CHILO GQ886826

Din2ARACH GQ886827

EafCOPE GQ886828

EfrDIPLUR GQ886829

EinEPHEM GQ886830

ErwONYCH GQ886831

EuryPAURO GQ886832

Han2SYMPH GQ886833

HariARACH GQ886834

HspARACH GQ886835

IpumARACH GQ886836

IveODONAT GQ886837

LemMALA GQ886838

LlyODONAT GQ886839

LnigARACH GQ886840

LoxTHECOS GQ886841

LynBRANCH GQ886842

MayEPHEM GQ886843

MbaARCHEO GQ886844

NeoMALA GQ886845

NmeZYGEN GQ886846

PamNEOPT GQ886847

PepONYCH GQ886848

Pma2ARACH GQ886849

Pno2ONYCH GQ886850

Pol2DIPLO GQ886851

PsaARCHEO GQ886852

ScolCHILO GQ886853

Scu3SYMPH GQ886854

SkleOST GQ886855

SpoCHILO GQ886856

UfsBRANCH GQ886857

6fin:

AarPENTA GQ886858

AchARACH GQ886859

AhiPYCNO GQ886860

AmaDIPLO GQ886861

Amb2ARACH GQ886862

Arg2BIURA GQ886863

Avu3MALA GQ886864

BbaTHECOS GQ886865

CfrTHECOS GQ886866

Col2PYCNO GQ886867

Cro2XIPHOS GQ886868

Crp2ARACH GQ886869

Ctas2CHILO GQ886870

DmaBRANCH GQ886871

DtyMYSTACO GQ886872

EfrDIPLUR GQ886873

EinEPHEM GQ886874

ErwONYCH GQ886875

EuryPAURO GQ886876

Han2SYMPH GQ886877

HariARACH GQ886878

HspARACH GQ886879

IpumARACH GQ886880

LemMALA GQ886881

LnigARACH GQ886882

LoxTHECOS GQ886883

MbaARCHEO GQ886884

MtdTARD GQ886885

NeoMALA GQ886886

OimCOLL GQ886887

PamNEOPT GQ886888

PepONYCH GQ886889

Pma2ARACH GQ886890

Pol2DIPLO GQ886891

PsaARCHEO GQ886892

ScolCHILO GQ886893

Scu3SYMPH GQ886894

Tom2COLL GQ886895

UfsBRANCH GQ886896

25fin:

A369COPE GQ886897

AdoNEOPT GQ886898

AeliPYCNO GQ886899

AhiPYCNO GQ886900

AmaDIPLO GQ886901

Amb2ARACH GQ886902

Arg2BIURA GQ886903

Asa3BRANCH GQ886904

BbaTHECOS GQ886905

CliZYGEN GQ886906

Col2PYCNO GQ886907

Cro2XIPHOS GQ886908

Crp2ARACH GQ886909

Ctas2CHILO GQ886910

Din2ARACH GQ886911

DmaBRANCH GQ886912

DtyMYSTACO GQ886913

EafCOPE GQ886914

EfrDIPLUR GQ886915

EgigARACH GQ886916

EinEPHEM GQ886917

ElePYCNO GQ886918

ErwONYCH GQ886919

EuryPAURO GQ886920

Han2SYMPH GQ886921

HariARACH GQ886922

HspARACH GQ886923

IpumARACH GQ886924

IveODONAT GQ886925

JapDIPLUR GQ886926

LemMALA GQ886927

Lle2BRANCH GQ886928

LlyODONAT GQ886929

LnigARACH GQ886930

LoxTHECOS GQ886931

LynBRANCH GQ886932

MayEPHEM GQ886933

MbaARCHEO GQ886934

MtdTARD GQ886935

NmeZYGEN GQ886936

OimCOLL GQ886937

PamNEOPT GQ886938

PepONYCH GQ886939

Pge2DIPLO GQ886940

Pma2ARACH GQ886941

Pno2ONYCH GQ886942

Pol2DIPLO GQ886943

PsaARCHEO GQ886944

PwhARACH GQ886945

ScolCHILO GQ886946

Scu3SYMPH GQ886947

SpoCHILO GQ886948

StpARACH GQ886949

Tom2COLL GQ886950

UfsBRANCH GQ886951

26fin:

A369COPE GQ886952

AarPENTA GQ886953

AchARACH GQ886954

AdoNEOPT GQ886955

AeliPYCNO GQ886956

AhiPYCNO GQ886957

AmaDIPLO GQ886958

Amb2ARACH GQ886959

Arg2BIURA GQ886960

Asa3BRANCH GQ886961

Avu3MALA GQ886962

BbaTHECOS GQ886963

CliZYGEN GQ886964

Col2PYCNO GQ886965

Cro2XIPHOS GQ886966

Crp2ARACH GQ886967

Ctas2CHILO GQ886968

Din2ARACH GQ886969

DmaBRANCH GQ886970

DtyMYSTACO GQ886971

EafCOPE GQ886972

EgigARACH GQ886973

EinEPHEM GQ886974

ElePYCNO GQ886975

ErwONYCH GQ886976

EuryPAURO GQ886977

Han2SYMPH GQ886978

HapaOST GQ886979

HariARACH GQ886980

HspARACH GQ886981

IpumARACH GQ886982

IveODONAT GQ886983

LeanTHECOS GQ886984

LemMALA GQ886985

Lle2BRANCH GQ886986

LlyODONAT GQ886987

LnigARACH GQ886988

LoxTHECOS GQ886989

LynBRANCH GQ886990

MayEPHEM GQ886991

MbaARCHEO GQ886992

NmeZYGEN GQ886993

PamNEOPT GQ886994

PepONYCH GQ886995

Pge2DIPLO GQ886996

Pma2ARACH GQ886997

Pno2ONYCH GQ886998

Pol2DIPLO GQ886999

PwhARACH GQ887000

ScolCHILO GQ887001

Scu3SYMPH GQ887002

SkleOST GQ887003

SpoCHILO GQ887004

StpARACH GQ887005

Tom2COLL GQ887006

UfsBRANCH GQ887007

36fin:

AdoNEOPT GQ887008

AeliPYCNO GQ887009

AhiPYCNO GQ887010

AmaDIPLO GQ887011

Amb2ARACH GQ887012

Asa3BRANCH GQ887013

Avu3MALA GQ887014

BbaTHECOS GQ887015

CliZYGEN GQ887016

Col2PYCNO GQ887017

Cro2XIPHOS GQ887018

Ctas2CHILO GQ887019

Din2ARACH GQ887020

DmaBRANCH GQ887021

EafCOPE GQ887022

EinEPHEM GQ887023

ErwONYCH GQ887024

EuryPAURO GQ887025

Han2SYMPH GQ887026

HariARACH GQ887027

IpumARACH GQ887028

IveODONAT GQ887029

JapDIPLUR GQ887030

LeanTHECOS GQ887031

LemMALA GQ887032

Lle2BRANCH GQ887033

LlyODONAT GQ887034

LnigARACH GQ887035

LoxTHECOS GQ887036

LynBRANCH GQ887037

MayEPHEM GQ887038

MbaARCHEO GQ887039

NeoMALA GQ887040

NmeZYGEN GQ887041

PamNEOPT GQ887042

PepONYCH GQ887043

Pge2DIPLO GQ887044

Pma2ARACH GQ887045

Pno2ONYCH GQ887046

Pol2DIPLO GQ887047

PsaARCHEO GQ887048

PwhARACH GQ887049

ScolCHILO GQ887050

Scu3SYMPH GQ887051

SkleOST GQ887052

SpoCHILO GQ887053

StpARACH GQ887054

Tom2COLL GQ887055

UfsBRANCH GQ887056

40fin:

A369COPE GQ887057

AarPENTA GQ887058

AchARACH GQ887059

AdoNEOPT GQ887060

AhiPYCNO GQ887061

AmaDIPLO GQ887062

Amb2ARACH GQ887063

Arg2BIURA GQ887064

Asa3BRANCH GQ887065

Avu3MALA GQ887066

BbaTHECOS GQ887067

CliZYGEN GQ887068

Col2PYCNO GQ887069

Cro2XIPHOS GQ887070

Crp2ARACH GQ887071

Ctas2CHILO GQ887072

Din2ARACH GQ887073

DmaBRANCH GQ887074

EafCOPE GQ887075

EfrDIPLUR GQ887076

EgigARACH GQ887077

EinEPHEM GQ887078

ErwONYCH GQ887079

EuryPAURO GQ887080

Han2SYMPH GQ887081

HariARACH GQ887082

HmaCEPHAL GQ887083

HspARACH GQ887084

IpumARACH GQ887085

IveODONAT GQ887086

JapDIPLUR GQ887087

LeanTHECOS GQ887088

LemMALA GQ887089

Lle2BRANCH GQ887090

LlyODONAT GQ887091

LnigARACH GQ887092

LoxTHECOS GQ887093

LynBRANCH GQ887094

MayEPHEM GQ887095

MbaARCHEO GQ887096

MtdTARD GQ887097

NeoMALA GQ887098

NmeZYGEN GQ887099

OimCOLL GQ887100

PamNEOPT GQ887101

PepONYCH GQ887102

Pge2DIPLO GQ887103

Pma2ARACH GQ887104

Pno2ONYCH GQ887105

Pol2DIPLO GQ887106

PsaARCHEO GQ887107

PwhARACH GQ887108

ScolCHILO GQ887109

Scu3SYMPH GQ887110

SkleOST GQ887111

SpoCHILO GQ887112

StpARACH GQ887113

UfsBRANCH GQ887114

42fin:

A369COPE GQ887115

AarPENTA GQ887116

AchARACH GQ887117

AdoNEOPT GQ887118

AeliPYCNO GQ887119

AhiPYCNO GQ887120

AmaDIPLO GQ887121

Amb2ARACH GQ887122

Arg2BIURA GQ887123

Asa3BRANCH GQ887124

Avu3MALA GQ887125

BbaTHECOS GQ887126

CfrTHECOS GQ887127

CliZYGEN GQ887128

Col2PYCNO GQ887129

Cro2XIPHOS GQ887130

Crp2ARACH GQ887131

Ctas2CHILO GQ887132

Din2ARACH GQ887133

DmaBRANCH GQ887134

DtyMYSTACO GQ887135

EafCOPE GQ887136

EgigARACH GQ887137

EinEPHEM GQ887138

ElePYCNO GQ887139

ErwONYCH GQ887140

Han2SYMPH GQ887141

HariARACH GQ887142

HspARACH GQ887143

IveODONAT GQ887144

JapDIPLUR GQ887145

LeanTHECOS GQ887146

LemMALA GQ887147

Lle2BRANCH GQ887148

LlyODONAT GQ887149

LoxTHECOS GQ887150

LynBRANCH GQ887151

MayEPHEM GQ887152

MbaARCHEO GQ887153

NeoMALA GQ887154

NmeZYGEN GQ887155

OimCOLL GQ887156

PamNEOPT GQ887157

PepONYCH GQ887158

Pge2DIPLO GQ887159

Pma2ARACH GQ887160

Pol2DIPLO GQ887161

PsaARCHEO GQ887162

PwhARACH GQ887163

ScolCHILO GQ887164

Scu3SYMPH GQ887165

SkleOST GQ887166

SpoCHILO GQ887167

StpARACH GQ887168

Tom2COLL GQ887169

UfsBRANCH GQ887170

44fin:

AchARACH GQ887171

AdoNEOPT GQ887172

AeliPYCNO GQ887173

AhiPYCNO GQ887174

AmaDIPLO GQ887175

Amb2ARACH GQ887176

Asa3BRANCH GQ887177

Avu3MALA GQ887178

BbaTHECOS GQ887179

CfrTHECOS GQ887180

CliZYGEN GQ887181

Col2PYCNO GQ887182

Cro2XIPHOS GQ887183

Crp2ARACH GQ887184

Ctas2CHILO GQ887185

Din2ARACH GQ887186

DmaBRANCH GQ887187

EafCOPE GQ887188

EinEPHEM GQ887189

Han2SYMPH GQ887190

HapaOST GQ887191

HariARACH GQ887192

HspARACH GQ887193

IpumARACH GQ887194

IveODONAT GQ887195

JapDIPLUR GQ887196

LemMALA GQ887197

Lle2BRANCH GQ887198

LlyODONAT GQ887199

LnigARACH GQ887200

LoxTHECOS GQ887201

LynBRANCH GQ887202

MayEPHEM GQ887203

MbaARCHEO GQ887204

MtdTARD GQ887205

NeoMALA GQ887206

NmeZYGEN GQ887207

PamNEOPT GQ887208

PepONYCH GQ887209

Pge2DIPLO GQ887210

Pma2ARACH GQ887211

Pol2DIPLO GQ887212

PwhARACH GQ887213

ScolCHILO GQ887214

Scu3SYMPH GQ887215

SkleOST GQ887216

SpoCHILO GQ887217

StpARACH GQ887218

Tom2COLL GQ887219

UfsBRANCH GQ887220

58fin3_6:

AarPENTA GQ887221

AchARACH GQ887222

AdoNEOPT GQ887223

AeliPYCNO GQ887224

AhiPYCNO GQ887225

AmaDIPLO GQ887226

Amb2ARACH GQ887227

Avu3MALA GQ887228

BbaTHECOS GQ887229

CliZYGEN GQ887230

Col2PYCNO GQ887231

Crp2ARACH GQ887232

Ctas2CHILO GQ887233

Din2ARACH GQ887234

DmaBRANCH GQ887235

DtyMYSTACO GQ887236

EafCOPE GQ887237

EfrDIPLUR GQ887238

EgigARACH GQ887239

EinEPHEM GQ887240

ElePYCNO GQ887241

ErwONYCH GQ887242

EuryPAURO GQ887243

Han2SYMPH GQ887244

HapaOST GQ887245

HariARACH GQ887246

HspARACH GQ887247

IveODONAT GQ887248

LeanTHECOS GQ887249

LemMALA GQ887250

Lle2BRANCH GQ887251

LlyODONAT GQ887252

LnigARACH GQ887253

LoxTHECOS GQ887254

LynBRANCH GQ887255

MayEPHEM GQ887256

MbaARCHEO GQ887257

MtdTARD GQ887258

NeoMALA GQ887259

NmeZYGEN GQ887260

PamNEOPT GQ887261

Pge2DIPLO GQ887262

Pma2ARACH GQ887263

Pol2DIPLO GQ887264

PsaARCHEO GQ887265

PwhARACH GQ887266

ScolCHILO GQ887267

Scu3SYMPH GQ887268

SkleOST GQ887269

SpoCHILO GQ887270

StpARACH GQ887271

Tom2COLL GQ887272

UfsBRANCH GQ887273

58fin7_9:

A369COPE GQ887274

AchARACH GQ887275

AdoNEOPT GQ887276

AeliPYCNO GQ887277

AhiPYCNO GQ887278

AmaDIPLO GQ887279

Amb2ARACH GQ887280

Arg2BIURA GQ887281

Avu3MALA GQ887282

BbaTHECOS GQ887283

CfrTHECOS GQ887284

CliZYGEN GQ887285

Col2PYCNO GQ887286

Crp2ARACH GQ887287

Ctas2CHILO GQ887288

Din2ARACH GQ887289

DmaBRANCH GQ887290

DtyMYSTACO GQ887291

EafCOPE GQ887292

EinEPHEM GQ887293

ElePYCNO GQ887294

ErwONYCH GQ887295

EuryPAURO GQ887296

Han2SYMPH GQ887297

HmaCEPHAL GQ887298

IveODONAT GQ887299

JapDIPLUR GQ887300

LeanTHECOS GQ887301

LemMALA GQ887302

Lle2BRANCH GQ887303

LlyODONAT GQ887304

LnigARACH GQ887305

LoxTHECOS GQ887306

MayEPHEM GQ887307

MbaARCHEO GQ887308

NeoMALA GQ887309

NmeZYGEN GQ887310

OimCOLL GQ887311

PamNEOPT GQ887312

PepONYCH GQ887313

Pge2DIPLO GQ887314

Pno2ONYCH GQ887315

Pol2DIPLO GQ887316

PsaARCHEO GQ887317

PwhARACH GQ887318

ScolCHILO GQ887319

Scu3SYMPH GQ887320

SkleOST GQ887321

SpoCHILO GQ887322

StpARACH GQ887323

Tom2COLL GQ887324

UfsBRANCH GQ887325

62fin:

A369COPE GQ887326

AarPENTA GQ887327

AchARACH GQ887328

AeliPYCNO GQ887329

AhiPYCNO GQ887330

AmaDIPLO GQ887331

Amb2ARACH GQ887332

Arg2BIURA GQ887333

Asa3BRANCH GQ887334

Avu3MALA GQ887335

CliZYGEN GQ887336

Col2PYCNO GQ887337

Cro2XIPHOS GQ887338

Crp2ARACH GQ887339

Ctas2CHILO GQ887340

Din2ARACH GQ887341

DmaBRANCH GQ887342

DtyMYSTACO GQ887343

EafCOPE GQ887344

EgigARACH GQ887345

EinEPHEM GQ887346

ElePYCNO GQ887347

ErwONYCH GQ887348

EuryPAURO GQ887349

Han2SYMPH GQ887350

IpumARACH GQ887351

IveODONAT GQ887352

LemMALA GQ887353

Lle2BRANCH GQ887354

LlyODONAT GQ887355

LnigARACH GQ887356

LynBRANCH GQ887357

MayEPHEM GQ887358

MbaARCHEO GQ887359

MtdTARD GQ887360

NeoMALA GQ887361

NmeZYGEN GQ887362

OimCOLL GQ887363

PamNEOPT GQ887364

PepONYCH GQ887365

Pge2DIPLO GQ887366

Pol2DIPLO GQ887367

PsaARCHEO GQ887368

PwhARACH GQ887369

ScolCHILO GQ887370

SkleOST GQ887371

SpoCHILO GQ887372

Tom2COLL GQ887373

UfsBRANCH GQ887374

63fin:

A369COPE GQ887375

AarPENTA GQ887376

AchARACH GQ887377

AdoNEOPT GQ887378

AeliPYCNO GQ887379

AhiPYCNO GQ887380

AmaDIPLO GQ887381

Arg2BIURA GQ887382

Asa3BRANCH GQ887383

Avu3MALA GQ887384

BbaTHECOS GQ887385

CfrTHECOS GQ887386

CliZYGEN GQ887387

Col2PYCNO GQ887388

Cro2XIPHOS GQ887389

Crp2ARACH GQ887390

Ctas2CHILO GQ887391

Din2ARACH GQ887392

DmaBRANCH GQ887393

DtyMYSTACO GQ887394

EafCOPE GQ887395

EgigARACH GQ887396

EinEPHEM GQ887397

ElePYCNO GQ887398

ErwONYCH GQ887399

EuryPAURO GQ887400

Han2SYMPH GQ887401

HapaOST GQ887402

HmaCEPHAL GQ887403

IpumARACH GQ887404

IveODONAT GQ887405

JapDIPLUR GQ887406

LeanTHECOS GQ887407

LemMALA GQ887408

Lle2BRANCH GQ887409

LlyODONAT GQ887410

LnigARACH GQ887411

LoxTHECOS GQ887412

LynBRANCH GQ887413

MayEPHEM GQ887414

MbaARCHEO GQ887415

MtdTARD GQ887416

NeoMALA GQ887417

NmeZYGEN GQ887418

OimCOLL GQ887419

PamNEOPT GQ887420

PepONYCH GQ887421

Pge2DIPLO GQ887422

Pma2ARACH GQ887423

Pno2ONYCH GQ887424

Pol2DIPLO GQ887425

PsaARCHEO GQ887426

PwhARACH GQ887427

ScolCHILO GQ887428

Scu3SYMPH GQ887429

SpoCHILO GQ887430

StpARACH GQ887431

Tom2COLL GQ887432

UfsBRANCH GQ887433

69fin:

A369COPE GQ887434

AarPENTA GQ887435

AchARACH GQ887436

AdoNEOPT GQ887437

AeliPYCNO GQ887438

AhiPYCNO GQ887439

AmaDIPLO GQ887440

Amb2ARACH GQ887441

Arg2BIURA GQ887442

Asa3BRANCH GQ887443

Avu3MALA GQ887444

BbaTHECOS GQ887445

CfrTHECOS GQ887446

CliZYGEN GQ887447

Col2PYCNO GQ887448

Cro2XIPHOS GQ887449

Crp2ARACH GQ887450

Ctas2CHILO GQ887451

Din2ARACH GQ887452

DtyMYSTACO GQ887453

EafCOPE GQ887454

EgigARACH GQ887455

EinEPHEM GQ887456

ElePYCNO GQ887457

ErwONYCH GQ887458

EuryPAURO GQ887459

Han2SYMPH GQ887460

HariARACH GQ887461

HspARACH GQ887462

IveODONAT GQ887463

JapDIPLUR GQ887464

LeanTHECOS GQ887465

LemMALA GQ887466

Lle2BRANCH GQ887467

LlyODONAT GQ887468

LnigARACH GQ887469

LoxTHECOS GQ887470

MayEPHEM GQ887471

MtdTARD GQ887472

NeoMALA GQ887473

NmeZYGEN GQ887474

OimCOLL GQ887475

PamNEOPT GQ887476

PepONYCH GQ887477

Pge2DIPLO GQ887478

Pma2ARACH GQ887479

Pno2ONYCH GQ887480

Pol2DIPLO GQ887481

PsaARCHEO GQ887482

ScolCHILO GQ887483

Scu3SYMPH GQ887484

SkleOST GQ887485

SpoCHILO GQ887486

StpARACH GQ887487

Tom2COLL GQ887488

UfsBRANCH GQ887489

73fin:

A369COPE GQ887490

AarPENTA GQ887491

AchARACH GQ887492

AdoNEOPT GQ887493

AeliPYCNO GQ887494

AhiPYCNO GQ887495

AmaDIPLO GQ887496

Amb2ARACH GQ887497

Arg2BIURA GQ887498

Asa3BRANCH GQ887499

Avu3MALA GQ887500

BbaTHECOS GQ887501

CfrTHECOS GQ887502

CliZYGEN GQ887503

Col2PYCNO GQ887504

Cro2XIPHOS GQ887505

Crp2ARACH GQ887506

Ctas2CHILO GQ887507

Din2ARACH GQ887508

DmaBRANCH GQ887509

EafCOPE GQ887510

EgigARACH GQ887511

EinEPHEM GQ887512

ElePYCNO GQ887513

ErwONYCH GQ887514

EuryPAURO GQ887515

Han2SYMPH GQ887516

HariARACH GQ887517

HspARACH GQ887518

IpumARACH GQ887519

IveODONAT GQ887520

LemMALA GQ887521

LlyODONAT GQ887522

LnigARACH GQ887523

LynBRANCH GQ887524

MayEPHEM GQ887525

MbaARCHEO GQ887526

NeoMALA GQ887527

NmeZYGEN GQ887528

PamNEOPT GQ887529

PepONYCH GQ887530

Pge2DIPLO GQ887531

Pma2ARACH GQ887532

Pol2DIPLO GQ887533

PsaARCHEO GQ887534

PwhARACH GQ887535

ScolCHILO GQ887536

Scu3SYMPH GQ887537

SkleOST GQ887538

SpoCHILO GQ887539

StpARACH GQ887540

Tom2COLL GQ887541

UfsBRANCH GQ887542

96fin:

A369COPE GQ887543

AarPENTA GQ887544

AchARACH GQ887545

AdoNEOPT GQ887546

AhiPYCNO GQ887547

AmaDIPLO GQ887548

Amb2ARACH GQ887549

Arg2BIURA GQ887550

Asa3BRANCH GQ887551

Avu3MALA GQ887552

BbaTHECOS GQ887553

CfrTHECOS GQ887554

CliZYGEN GQ887555

Col2PYCNO GQ887556

Crp2ARACH GQ887557

Ctas2CHILO GQ887558

Din2ARACH GQ887559

DmaBRANCH GQ887560

EafCOPE GQ887561

EfrDIPLUR GQ887562

EgigARACH GQ887563

EinEPHEM GQ887564

ErwONYCH GQ887565

EuryPAURO GQ887566

Han2SYMPH GQ887567

HapaOST GQ887568

HariARACH GQ887569

HmaCEPHAL GQ887570

HspARACH GQ887571

IveODONAT GQ887572

JapDIPLUR GQ887573

LeanTHECOS GQ887574

LemMALA GQ887575

LlyODONAT GQ887576

LnigARACH GQ887577

LoxTHECOS GQ887578

LynBRANCH GQ887579

MayEPHEM GQ887580

MbaARCHEO GQ887581

MtdTARD GQ887582

NeoMALA GQ887583

NmeZYGEN GQ887584

OimCOLL GQ887585

PamNEOPT GQ887586

PepONYCH GQ887587

Pge2DIPLO GQ887588

Pma2ARACH GQ887589

Pno2ONYCH GQ887590

PsaARCHEO GQ887591

PwhARACH GQ887592

ScolCHILO GQ887593

Scu3SYMPH GQ887594

StpARACH GQ887595

Tom2COLL GQ887596

UfsBRANCH GQ887597

109fin:

AarPENTA GQ887598

AchARACH GQ887599

AdoNEOPT GQ887600

AeliPYCNO GQ887601

AmaDIPLO GQ887602

Amb2ARACH GQ887603

Arg2BIURA GQ887604

Asa3BRANCH GQ887605

Avu3MALA GQ887606

CliZYGEN GQ887607

Col2PYCNO GQ887608

Cro2XIPHOS GQ887609

Crp2ARACH GQ887610

Ctas2CHILO GQ887611

Din2ARACH GQ887612

DtyMYSTACO GQ887613

EafCOPE GQ887614

EgigARACH GQ887615

EinEPHEM GQ887616

ElePYCNO GQ887617

ErwONYCH GQ887618

EuryPAURO GQ887619

Han2SYMPH GQ887620

HapaOST GQ887621

HariARACH GQ887622

HspARACH GQ887623

IveODONAT GQ887624

JapDIPLUR GQ887625

LeanTHECOS GQ887626

LemMALA GQ887627

Lle2BRANCH GQ887628

LnigARACH GQ887629

LoxTHECOS GQ887630

MayEPHEM GQ887631

MbaARCHEO GQ887632

MtdTARD GQ887633

NeoMALA GQ887634

OimCOLL GQ887635

PamNEOPT GQ887636

PepONYCH GQ887637

Pge2DIPLO GQ887638

Pma2ARACH GQ887639

Pno2ONYCH GQ887640

Pol2DIPLO GQ887641

PsaARCHEO GQ887642

PwhARACH GQ887643

ScolCHILO GQ887644

Scu3SYMPH GQ887645

SpoCHILO GQ887646

StpARACH GQ887647

Tom2COLL GQ887648

UfsBRANCH GQ887649

113fin:

A369COPE GQ887650

AarPENTA GQ887651

AchARACH GQ887652

AdoNEOPT GQ887653

AeliPYCNO GQ887654

AmaDIPLO GQ887655

Arg2BIURA GQ887656

Avu3MALA GQ887657

BbaTHECOS GQ887658

CfrTHECOS GQ887659

CliZYGEN GQ887660

Col2PYCNO GQ887661

Cro2XIPHOS GQ887662

Crp2ARACH GQ887663

Ctas2CHILO GQ887664

Din2ARACH GQ887665

DmaBRANCH GQ887666

DtyMYSTACO GQ887667

EafCOPE GQ887668

EfrDIPLUR GQ887669

EinEPHEM GQ887670

ElePYCNO GQ887671

ErwONYCH GQ887672

EuryPAURO GQ887673

Han2SYMPH GQ887674

HariARACH GQ887675

HspARACH GQ887676

IpumARACH GQ887677

IveODONAT GQ887678

JapDIPLUR GQ887679

LeanTHECOS GQ887680

LemMALA GQ887681

Lle2BRANCH GQ887682

LlyODONAT GQ887683

LnigARACH GQ887684

LoxTHECOS GQ887685

LynBRANCH GQ887686

MbaARCHEO GQ887687

MtdTARD GQ887688

NeoMALA GQ887689

NmeZYGEN GQ887690

OimCOLL GQ887691

PamNEOPT GQ887692

PepONYCH GQ887693

Pge2DIPLO GQ887694

Pma2ARACH GQ887695

PsaARCHEO GQ887696

PwhARACH GQ887697

ScolCHILO GQ887698

Scu3SYMPH GQ887699

SkleOST GQ887700

SpoCHILO GQ887701

StpARACH GQ887702

UfsBRANCH GQ887703

127fin:

AchARACH GQ887704

AdoNEOPT GQ887705

AeliPYCNO GQ887706

AhiPYCNO GQ887707

AmaDIPLO GQ887708

Amb2ARACH GQ887709

Arg2BIURA GQ887710

Asa3BRANCH GQ887711

Avu3MALA GQ887712

BbaTHECOS GQ887713

CliZYGEN GQ887714

Col2PYCNO GQ887715

Cro2XIPHOS GQ887716

Crp2ARACH GQ887717

DmaBRANCH GQ887718

EfrDIPLUR GQ887719

EgigARACH GQ887720

EinEPHEM GQ887721

ElePYCNO GQ887722

ErwONYCH GQ887723

EuryPAURO GQ887724

Han2SYMPH GQ887725

HariARACH GQ887726

HspARACH GQ887727

IveODONAT GQ887728

JapDIPLUR GQ887729

Lle2BRANCH GQ887730

LlyODONAT GQ887731

LnigARACH GQ887732

LoxTHECOS GQ887733

LynBRANCH GQ887734

MayEPHEM GQ887735

MbaARCHEO GQ887736

MtdTARD GQ887737

NeoMALA GQ887738

NmeZYGEN GQ887739

OimCOLL GQ887740

PamNEOPT GQ887741

PepONYCH GQ887742

Pge2DIPLO GQ887743

Pma2ARACH GQ887744

Pol2DIPLO GQ887745

PsaARCHEO GQ887746

ScolCHILO GQ887747

Scu3SYMPH GQ887748

Tom2COLL GQ887749

UfsBRANCH GQ887750

149fin:

A369COPE GQ887751

AarPENTA GQ887752

AchARACH GQ887753

AdoNEOPT GQ887754

AeliPYCNO GQ887755

AhiPYCNO GQ887756

AmaDIPLO GQ887757

Amb2ARACH GQ887758

Arg2BIURA GQ887759

Asa3BRANCH GQ887760

Avu3MALA GQ887761

BbaTHECOS GQ887762

CfrTHECOS GQ887763

CliZYGEN GQ887764

Col2PYCNO GQ887765

Cro2XIPHOS GQ887766

Crp2ARACH GQ887767

Ctas2CHILO GQ887768

Din2ARACH GQ887769

DmaBRANCH GQ887770

DtyMYSTACO GQ887771

EafCOPE GQ887772

EfrDIPLUR GQ887773

EgigARACH GQ887774

ElePYCNO GQ887775

EuryPAURO GQ887776

Han2SYMPH GQ887777

HariARACH GQ887778

HspARACH GQ887779

IpumARACH GQ887780

IveODONAT GQ887781

LeanTHECOS GQ887782

LemMALA GQ887783

Lle2BRANCH GQ887784

LlyODONAT GQ887785

LnigARACH GQ887786

LoxTHECOS GQ887787

LynBRANCH GQ887788

MayEPHEM GQ887789

MbaARCHEO GQ887790

NeoMALA GQ887791

NmeZYGEN GQ887792

OimCOLL GQ887793

PamNEOPT GQ887794

Pma2ARACH GQ887795

Pol2DIPLO GQ887796

PsaARCHEO GQ887797

PwhARACH GQ887798

Scu3SYMPH GQ887799

SkleOST GQ887800

SpoCHILO GQ887801

StpARACH GQ887802

Tom2COLL GQ887803

UfsBRANCH GQ887804

166fin:

A369COPE GQ887805

AarPENTA GQ887806

AchARACH GQ887807

AdoNEOPT GQ887808

AeliPYCNO GQ887809

AhiPYCNO GQ887810

AmaDIPLO GQ887811

Amb2ARACH GQ887812

Arg2BIURA GQ887813

Asa3BRANCH GQ887814

Avu3MALA GQ887815

BbaTHECOS GQ887816

CfrTHECOS GQ887817

CliZYGEN GQ887818

Col2PYCNO GQ887819

Cro2XIPHOS GQ887820

Crp2ARACH GQ887821

Ctas2CHILO GQ887822

Din2ARACH GQ887823

DmaBRANCH GQ887824

DtyMYSTACO GQ887825

EafCOPE GQ887826

EfrDIPLUR GQ887827

EgigARACH GQ887828

EinEPHEM GQ887829

ElePYCNO GQ887830

ErwONYCH GQ887831

EuryPAURO GQ887832

Han2SYMPH GQ887833

HapaOST GQ887834

HariARACH GQ887835

HmaCEPHAL GQ887836

HspARACH GQ887837

IpumARACH GQ887838

IveODONAT GQ887839

JapDIPLUR GQ887840

LeanTHECOS GQ887841

LemMALA GQ887842

Lle2BRANCH GQ887843

LlyODONAT GQ887844

LnigARACH GQ887845

LoxTHECOS GQ887846

LynBRANCH GQ887847

MayEPHEM GQ887848

MbaARCHEO GQ887849

MtdTARD GQ887850

NeoMALA GQ887851

NmeZYGEN GQ887852

OimCOLL GQ887853

PamNEOPT GQ887854

PepONYCH GQ887855

Pge2DIPLO GQ887856

Pma2ARACH GQ887857

Pno2ONYCH GQ887858

Pol2DIPLO GQ887859

PwhARACH GQ887860

ScolCHILO GQ887861

Scu3SYMPH GQ887862

SkleOST GQ887863

SpoCHILO GQ887864

StpARACH GQ887865

Tom2COLL GQ887866

UfsBRANCH GQ887867

192fin:

AchARACH GQ887868

AeliPYCNO GQ887869

AhiPYCNO GQ887870

Amb2ARACH GQ887871

Arg2BIURA GQ887872

Asa3BRANCH GQ887873

BbaTHECOS GQ887874

CfrTHECOS GQ887875

CliZYGEN GQ887876

Cro2XIPHOS GQ887877

Crp2ARACH GQ887878

Ctas2CHILO GQ887879

Din2ARACH GQ887880

DmaBRANCH GQ887881

DtyMYSTACO GQ887882

EafCOPE GQ887883

EfrDIPLUR GQ887884

EgigARACH GQ887885

EinEPHEM GQ887886

ElePYCNO GQ887887

ErwONYCH GQ887888

EuryPAURO GQ887889

Han2SYMPH GQ887890

HapaOST GQ887891

HariARACH GQ887892

HspARACH GQ887893

IpumARACH GQ887894

JapDIPLUR GQ887895

LemMALA GQ887896

Lle2BRANCH GQ887897

LnigARACH GQ887898

LoxTHECOS GQ887899

LynBRANCH GQ887900

MayEPHEM GQ887901

MbaARCHEO GQ887902

NeoMALA GQ887903

NmeZYGEN GQ887904

PamNEOPT GQ887905

PepONYCH GQ887906

Pge2DIPLO GQ887907

Pma2ARACH GQ887908

Pol2DIPLO GQ887909

PsaARCHEO GQ887910

PwhARACH GQ887911

ScolCHILO GQ887912

Scu3SYMPH GQ887913

SkleOST GQ887914

SpoCHILO GQ887915

StpARACH GQ887916

Tom2COLL GQ887917

UfsBRANCH GQ887918

197fin:

A369COPE GQ887919

AarPENTA GQ887920

AchARACH GQ887921

AdoNEOPT GQ887922

AeliPYCNO GQ887923

AhiPYCNO GQ887924

Amb2ARACH GQ887925

Arg2BIURA GQ887926

Avu3MALA GQ887927

BbaTHECOS GQ887928

CliZYGEN GQ887929

Col2PYCNO GQ887930

Cro2XIPHOS GQ887931

Crp2ARACH GQ887932

Ctas2CHILO GQ887933

Din2ARACH GQ887934

DmaBRANCH GQ887935

EafCOPE GQ887936

EfrDIPLUR GQ887937

EgigARACH GQ887938

EinEPHEM GQ887939

ErwONYCH GQ887940

EuryPAURO GQ887941

Han2SYMPH GQ887942

HariARACH GQ887943

HmaCEPHAL GQ887944

HspARACH GQ887945

IveODONAT GQ887946

JapDIPLUR GQ887947

LeanTHECOS GQ887948

LemMALA GQ887949

Lle2BRANCH GQ887950

LlyODONAT GQ887951

LnigARACH GQ887952

LynBRANCH GQ887953

MayEPHEM GQ887954

MbaARCHEO GQ887955

NeoMALA GQ887956

NmeZYGEN GQ887957

OimCOLL GQ887958

PepONYCH GQ887959

Pge2DIPLO GQ887960

Pma2ARACH GQ887961

Pol2DIPLO GQ887962

PsaARCHEO GQ887963

ScolCHILO GQ887964

Scu3SYMPH GQ887965

SpoCHILO GQ887966

StpARACH GQ887967

220fin:

AchARACH GQ887968

AdoNEOPT GQ887969

AeliPYCNO GQ887970

AhiPYCNO GQ887971

AmaDIPLO GQ887972

Amb2ARACH GQ887973

Arg2BIURA GQ887974

Asa3BRANCH GQ887975

Avu3MALA GQ887976

BbaTHECOS GQ887977

CfrTHECOS GQ887978

Col2PYCNO GQ887979

Crp2ARACH GQ887980

Ctas2CHILO GQ887981

Din2ARACH GQ887982

DmaBRANCH GQ887983

DtyMYSTACO GQ887984

EfrDIPLUR GQ887985

EgigARACH GQ887986

EinEPHEM GQ887987

ElePYCNO GQ887988

ErwONYCH GQ887989

EuryPAURO GQ887990

Han2SYMPH GQ887991

HariARACH GQ887992

HspARACH GQ887993

IveODONAT GQ887994

LeanTHECOS GQ887995

LemMALA GQ887996

Lle2BRANCH GQ887997

LlyODONAT GQ887998

LnigARACH GQ887999

MayEPHEM GQ888000

MbaARCHEO GQ888001

NeoMALA GQ888002

NmeZYGEN GQ888003

OimCOLL GQ888004

PamNEOPT GQ888005

PepONYCH GQ888006

Pge2DIPLO GQ888007

Pma2ARACH GQ888008

Pol2DIPLO GQ888009

PsaARCHEO GQ888010

PwhARACH GQ888011

ScolCHILO GQ888012

Scu3SYMPH GQ888013

SpoCHILO GQ888014

StpARACH GQ888015

Tom2COLL GQ888016

UfsBRANCH GQ888017

226fin:

A369COPE GQ888018

AarPENTA GQ888019

AchARACH GQ888020

AeliPYCNO GQ888021

AhiPYCNO GQ888022

AmaDIPLO GQ888023

Amb2ARACH GQ888024

Arg2BIURA GQ888025

Asa3BRANCH GQ888026

Avu3MALA GQ888027

BbaTHECOS GQ888028

CliZYGEN GQ888029

Col2PYCNO GQ888030

Cro2XIPHOS GQ888031

Crp2ARACH GQ888032

Ctas2CHILO GQ888033

Din2ARACH GQ888034

DmaBRANCH GQ888035

DtyMYSTACO GQ888036

EafCOPE GQ888037

EfrDIPLUR GQ888038

EgigARACH GQ888039

EinEPHEM GQ888040

ElePYCNO GQ888041

ErwONYCH GQ888042

EuryPAURO GQ888043

Han2SYMPH GQ888044

HariARACH GQ888045

HspARACH GQ888046

IpumARACH GQ888047

IveODONAT GQ888048

JapDIPLUR GQ888049

LeanTHECOS GQ888050

LemMALA GQ888051

Lle2BRANCH GQ888052

LlyODONAT GQ888053

LnigARACH GQ888054

LoxTHECOS GQ888055

LynBRANCH GQ888056

MayEPHEM GQ888057

MbaARCHEO GQ888058

MtdTARD GQ888059

NeoMALA GQ888060

NmeZYGEN GQ888061

OimCOLL GQ888062

PamNEOPT GQ888063

PepONYCH GQ888064

Pge2DIPLO GQ888065

Pma2ARACH GQ888066

Pol2DIPLO GQ888067

PsaARCHEO GQ888068

PwhARACH GQ888069

ScolCHILO GQ888070

Scu3SYMPH GQ888071

SkleOST GQ888072

SpoCHILO GQ888073

StpARACH GQ888074

Tom2COLL GQ888075

UfsBRANCH GQ888076

247fin:

A369COPE GQ888077

AchARACH GQ888078

AdoNEOPT GQ888079

AeliPYCNO GQ888080

AhiPYCNO GQ888081

AmaDIPLO GQ888082

Asa3BRANCH GQ888083

Avu3MALA GQ888084

CfrTHECOS GQ888085

CliZYGEN GQ888086

Ctas2CHILO GQ888087

Din2ARACH GQ888088

DmaBRANCH GQ888089

EafCOPE GQ888090

EfrDIPLUR GQ888091

EinEPHEM GQ888092

ElePYCNO GQ888093

ErwONYCH GQ888094

EuryPAURO GQ888095

Han2SYMPH GQ888096

HariARACH GQ888097

HmaCEPHAL GQ888098

HspARACH GQ888099

IpumARACH GQ888100

IveODONAT GQ888101

JapDIPLUR GQ888102

LeanTHECOS GQ888103

LlyODONAT GQ888104

LnigARACH GQ888105

LoxTHECOS GQ888106

MayEPHEM GQ888107

NeoMALA GQ888108

NmeZYGEN GQ888109

OimCOLL GQ888110

PamNEOPT GQ888111

PepONYCH GQ888112

Pma2ARACH GQ888113

Pol2DIPLO GQ888114

PsaARCHEO GQ888115

PwhARACH GQ888116

ScolCHILO GQ888117

Scu3SYMPH GQ888118

SpoCHILO GQ888119

Tom2COLL GQ888120

UfsBRANCH GQ888121

262fin:

A369COPE GQ888122

AarPENTA GQ888123

AchARACH GQ888124

AdoNEOPT GQ888125

AeliPYCNO GQ888126

AhiPYCNO GQ888127

AmaDIPLO GQ888128

Arg2BIURA GQ888129

Asa3BRANCH GQ888130

Avu3MALA GQ888131

CfrTHECOS GQ888132

CliZYGEN GQ888133

Col2PYCNO GQ888134

Cro2XIPHOS GQ888135

Crp2ARACH GQ888136

Din2ARACH GQ888137

DmaBRANCH GQ888138

DtyMYSTACO GQ888139

EafCOPE GQ888140

EgigARACH GQ888141

EinEPHEM GQ888142

ElePYCNO GQ888143

ErwONYCH GQ888144

EuryPAURO GQ888145

Han2SYMPH GQ888146

HariARACH GQ888147

HmaCEPHAL GQ888148

HspARACH GQ888149

IpumARACH GQ888150

IveODONAT GQ888151

JapDIPLUR GQ888152

LeanTHECOS GQ888153

LemMALA GQ888154

Lle2BRANCH GQ888155

LlyODONAT GQ888156

LnigARACH GQ888157

LoxTHECOS GQ888158

LynBRANCH GQ888159

MbaARCHEO GQ888160

NeoMALA GQ888161

NmeZYGEN GQ888162

OimCOLL GQ888163

PamNEOPT GQ888164

PepONYCH GQ888165

Pge2DIPLO GQ888166

Pma2ARACH GQ888167

Pno2ONYCH GQ888168

Pol2DIPLO GQ888169

PsaARCHEO GQ888170

PwhARACH GQ888171

ScolCHILO GQ888172

Scu3SYMPH GQ888173

SkleOST GQ888174

SpoCHILO GQ888175

StpARACH GQ888176

Tom2COLL GQ888177

UfsBRANCH GQ888178

265fin:

A369COPE GQ888179

AeliPYCNO GQ888180

AhiPYCNO GQ888181

AmaDIPLO GQ888182

Arg2BIURA GQ888183

Asa3BRANCH GQ888184

Avu3MALA GQ888185

BbaTHECOS GQ888186

CliZYGEN GQ888187

Crp2ARACH GQ888188

Ctas2CHILO GQ888189

Din2ARACH GQ888190

DmaBRANCH GQ888191

EafCOPE GQ888192

EfrDIPLUR GQ888193

EgigARACH GQ888194

EinEPHEM GQ888195

ErwONYCH GQ888196

EuryPAURO GQ888197

Han2SYMPH GQ888198

HariARACH GQ888199

HmaCEPHAL GQ888200

HspARACH GQ888201

IveODONAT GQ888202

JapDIPLUR GQ888203

LeanTHECOS GQ888204

LemMALA GQ888205

LlyODONAT GQ888206

LnigARACH GQ888207

LoxTHECOS GQ888208

LynBRANCH GQ888209

MayEPHEM GQ888210

MbaARCHEO GQ888211

MtdTARD GQ888212

NeoMALA GQ888213

NmeZYGEN GQ888214

OimCOLL GQ888215

PamNEOPT GQ888216

PepONYCH GQ888217

Pge2DIPLO GQ888218

Pma2ARACH GQ888219

Pno2ONYCH GQ888220

Pol2DIPLO GQ888221

PsaARCHEO GQ888222

PwhARACH GQ888223

Scu3SYMPH GQ888224

SkleOST GQ888225

StpARACH GQ888226

Tom2COLL GQ888227

267fin:

A369COPE GQ888228

AchARACH GQ888229

AdoNEOPT GQ888230

AeliPYCNO GQ888231

AhiPYCNO GQ888232

AmaDIPLO GQ888233

Amb2ARACH GQ888234

Arg2BIURA GQ888235

Asa3BRANCH GQ888236

Avu3MALA GQ888237

BbaTHECOS GQ888238

CfrTHECOS GQ888239

CliZYGEN GQ888240

Col2PYCNO GQ888241

Cro2XIPHOS GQ888242

Crp2ARACH GQ888243

Ctas2CHILO GQ888244

Din2ARACH GQ888245

DmaBRANCH GQ888246

EafCOPE GQ888247

EfrDIPLUR GQ888248

EgigARACH GQ888249

EinEPHEM GQ888250

ElePYCNO GQ888251

ErwONYCH GQ888252

EuryPAURO GQ888253

Han2SYMPH GQ888254

HmaCEPHAL GQ888255

HspARACH GQ888256

IpumARACH GQ888257

IveODONAT GQ888258

JapDIPLUR GQ888259

LeanTHECOS GQ888260

LemMALA GQ888261

Lle2BRANCH GQ888262

LnigARACH GQ888263

LoxTHECOS GQ888264

LynBRANCH GQ888265

MayEPHEM GQ888266

MbaARCHEO GQ888267

MtdTARD GQ888268

NeoMALA GQ888269

NmeZYGEN GQ888270

OimCOLL GQ888271

PamNEOPT GQ888272

PepONYCH GQ888273

Pge2DIPLO GQ888274

Pma2ARACH GQ888275

Pno2ONYCH GQ888276

Pol2DIPLO GQ888277

PsaARCHEO GQ888278

PwhARACH GQ888279

ScolCHILO GQ888280

Scu3SYMPH GQ888281

SkleOST GQ888282

SpoCHILO GQ888283

StpARACH GQ888284

Tom2COLL GQ888285

UfsBRANCH GQ888286

268fin:

A369COPE GQ888287

AdoNEOPT GQ888288

AeliPYCNO GQ888289

AhiPYCNO GQ888290

AmaDIPLO GQ888291

Amb2ARACH GQ888292

Arg2BIURA GQ888293

Col2PYCNO GQ888294

Cro2XIPHOS GQ888295

Crp2ARACH GQ888296

Ctas2CHILO GQ888297

Din2ARACH GQ888298

DmaBRANCH GQ888299

EafCOPE GQ888300

EgigARACH GQ888301

EinEPHEM GQ888302

ErwONYCH GQ888303

Han2SYMPH GQ888304

IpumARACH GQ888305

IveODONAT GQ888306

Lle2BRANCH GQ888307

LlyODONAT GQ888308

LnigARACH GQ888309

LoxTHECOS GQ888310

LynBRANCH GQ888311

MayEPHEM GQ888312

MbaARCHEO GQ888313

NmeZYGEN GQ888314

OimCOLL GQ888315

PamNEOPT GQ888316

PepONYCH GQ888317

Pge2DIPLO GQ888318

Pol2DIPLO GQ888319

PsaARCHEO GQ888320

PwhARACH GQ888321

ScolCHILO GQ888322

Scu3SYMPH GQ888323

SkleOST GQ888324

StpARACH GQ888325

Tom2COLL GQ888326

UfsBRANCH GQ888327

270fin:

AchARACH GQ888328

AdoNEOPT GQ888329

Amb2ARACH GQ888330

Avu3MALA GQ888331

BbaTHECOS GQ888332

CfrTHECOS GQ888333

CliZYGEN GQ888334

Col2PYCNO GQ888335

Cro2XIPHOS GQ888336

Crp2ARACH GQ888337

Ctas2CHILO GQ888338

Din2ARACH GQ888339

DmaBRANCH GQ888340

DtyMYSTACO GQ888341

EafCOPE GQ888342

EgigARACH GQ888343

ElePYCNO GQ888344

ErwONYCH GQ888345

Han2SYMPH GQ888346

HariARACH GQ888347

HspARACH GQ888348

IveODONAT GQ888349

JapDIPLUR GQ888350

LeanTHECOS GQ888351

LemMALA GQ888352

LlyODONAT GQ888353

LnigARACH GQ888354

LoxTHECOS GQ888355

LynBRANCH GQ888356

MayEPHEM GQ888357

MbaARCHEO GQ888358

NeoMALA GQ888359

NmeZYGEN GQ888360

OimCOLL GQ888361

PamNEOPT GQ888362

Pma2ARACH GQ888363

PsaARCHEO GQ888364

ScolCHILO GQ888365

SkleOST GQ888366

SpoCHILO GQ888367

StpARACH GQ888368

Tom2COLL GQ888369

274fin:

A369COPE GQ888370

AarPENTA GQ888371

AchARACH GQ888372

AdoNEOPT GQ888373

AmaDIPLO GQ888374

Amb2ARACH GQ888375

Arg2BIURA GQ888376

Asa3BRANCH GQ888377

BbaTHECOS GQ888378

CfrTHECOS GQ888379

Crp2ARACH GQ888380

Ctas2CHILO GQ888381

Din2ARACH GQ888382

DmaBRANCH GQ888383

DtyMYSTACO GQ888384

EinEPHEM GQ888385

EuryPAURO GQ888386

Han2SYMPH GQ888387

HmaCEPHAL GQ888388

HspARACH GQ888389

IveODONAT GQ888390

LeanTHECOS GQ888391

LemMALA GQ888392

Lle2BRANCH GQ888393

LlyODONAT GQ888394

LoxTHECOS GQ888395

LynBRANCH GQ888396

MayEPHEM GQ888397

PamNEOPT GQ888398

PepONYCH GQ888399

Pge2DIPLO GQ888400

Pol2DIPLO GQ888401

PwhARACH GQ888402

ScolCHILO GQ888403

Scu3SYMPH GQ888404

SkleOST GQ888405

SpoCHILO GQ888406

UfsBRANCH GQ888407

3006fin:

A369COPE GQ888408

AarPENTA GQ888409

AchARACH GQ888410

AdoNEOPT GQ888411

AeliPYCNO GQ888412

AhiPYCNO GQ888413

AmaDIPLO GQ888414

Amb2ARACH GQ888415

Arg2BIURA GQ888416

Avu3MALA GQ888417

BbaTHECOS GQ888418

CfrTHECOS GQ888419

CliZYGEN GQ888420

Col2PYCNO GQ888421

Crp2ARACH GQ888422

Ctas2CHILO GQ888423

EafCOPE GQ888424

EfrDIPLUR GQ888425

EinEPHEM GQ888426

ElePYCNO GQ888427

ErwONYCH GQ888428

EuryPAURO GQ888429

Han2SYMPH GQ888430

HapaOST GQ888431

HariARACH GQ888432

HmaCEPHAL GQ888433

HspARACH GQ888434

IveODONAT GQ888435

JapDIPLUR GQ888436

LeanTHECOS GQ888437

LemMALA GQ888438

LlyODONAT GQ888439

LnigARACH GQ888440

LoxTHECOS GQ888441

MayEPHEM GQ888442

MbaARCHEO GQ888443

NeoMALA GQ888444

NmeZYGEN GQ888445

OimCOLL GQ888446

PamNEOPT GQ888447

PepONYCH GQ888448

Pge2DIPLO GQ888449

Pno2ONYCH GQ888450

Pol2DIPLO GQ888451

PsaARCHEO GQ888452

ScolCHILO GQ888453

Scu3SYMPH GQ888454

SkleOST GQ888455

SpoCHILO GQ888456

Tom2COLL GQ888457

UfsBRANCH GQ888458

3007fin:

A369COPE GQ888459

AchARACH GQ888460

AdoNEOPT GQ888461

AhiPYCNO GQ888462

AmaDIPLO GQ888463

Amb2ARACH GQ888464

Arg2BIURA GQ888465

Avu3MALA GQ888466

BbaTHECOS GQ888467

CfrTHECOS GQ888468

Crp2ARACH GQ888469

Ctas2CHILO GQ888470

Din2ARACH GQ888471

DmaBRANCH GQ888472

DtyMYSTACO GQ888473

EafCOPE GQ888474

EfrDIPLUR GQ888475

EgigARACH GQ888476

ErwONYCH GQ888477

EuryPAURO GQ888478

Han2SYMPH GQ888479

HapaOST GQ888480

HspARACH GQ888481

IpumARACH GQ888482

IveODONAT GQ888483

JapDIPLUR GQ888484

LemMALA GQ888485

Lle2BRANCH GQ888486

LlyODONAT GQ888487

LnigARACH GQ888488

LoxTHECOS GQ888489

LynBRANCH GQ888490

MayEPHEM GQ888491

MbaARCHEO GQ888492

NeoMALA GQ888493

NmeZYGEN GQ888494

OimCOLL GQ888495

PamNEOPT GQ888496

PepONYCH GQ888497

Pge2DIPLO GQ888498

Pma2ARACH GQ888499

PsaARCHEO GQ888500

PwhARACH GQ888501

ScolCHILO GQ888502

Scu3SYMPH GQ888503

SpoCHILO GQ888504

Tom2COLL GQ888505

UfsBRANCH GQ888506

3009fin:

A369COPE GQ885174

AarPENTA GQ885175

AchARACH GQ885176

AdoNEOPT GQ885177

AeliPYCNO GQ885178

AhiPYCNO GQ885179

Arg2BIURA GQ885180

Asa3BRANCH GQ885181

Avu3MALA GQ885182

CliZYGEN GQ885183

Col2PYCNO GQ885184

Crp2ARACH GQ885185

Ctas2CHILO GQ885186

Din2ARACH GQ885187

DmaBRANCH GQ885188

DtyMYSTACO GQ885189

EafCOPE GQ885190

EfrDIPLUR GQ885191

EgigARACH GQ885192

EinEPHEM GQ885193

ElePYCNO GQ885194

ErwONYCH GQ885195

Han2SYMPH GQ885196

HariARACH GQ885197

HmaCEPHAL GQ885198

HspARACH GQ885199

IpumARACH GQ885200

IveODONAT GQ885201

JapDIPLUR GQ885202

LemMALA GQ885203

LnigARACH GQ885204

MayEPHEM GQ885205

MbaARCHEO GQ885206

MtdTARD GQ885207

NeoMALA GQ885208

NmeZYGEN GQ885209

OimCOLL GQ885210

PepONYCH GQ885211

Pge2DIPLO GQ885212

Pma2ARACH GQ885213

PsaARCHEO GQ885214

PwhARACH GQ885215

ScolCHILO GQ885216

Scu3SYMPH GQ885217

SkleOST GQ885218

SpoCHILO GQ885219

StpARACH GQ885220

Tom2COLL GQ885221

UfsBRANCH GQ885222

3012fin:

A369COPE GQ885223

AdoNEOPT GQ885224

AeliPYCNO GQ885225

AhiPYCNO GQ885226

AmaDIPLO GQ885227

Amb2ARACH GQ885228

Arg2BIURA GQ885229

Asa3BRANCH GQ885230

Avu3MALA GQ885231

BbaTHECOS GQ885232

CfrTHECOS GQ885233

CliZYGEN GQ885234

Col2PYCNO GQ885235

Cro2XIPHOS GQ885236

Ctas2CHILO GQ885237

Din2ARACH GQ885238

DmaBRANCH GQ885239

EafCOPE GQ885240

EfrDIPLUR GQ885241

EinEPHEM GQ885242

ElePYCNO GQ885243

ErwONYCH GQ885244

EuryPAURO GQ885245

Han2SYMPH GQ885246

HapaOST GQ885247

HariARACH GQ885248

HmaCEPHAL GQ885249

HspARACH GQ885250

IpumARACH GQ885251

JapDIPLUR GQ885252

LemMALA GQ885253

Lle2BRANCH GQ885254

LoxTHECOS GQ885255

LynBRANCH GQ885256

MbaARCHEO GQ885257

MtdTARD GQ885258

NeoMALA GQ885259

NmeZYGEN GQ885260

OimCOLL GQ885261

PepONYCH GQ885262

Pma2ARACH GQ885263

Pol2DIPLO GQ885264

PsaARCHEO GQ885265

PwhARACH GQ885266

ScolCHILO GQ885267

Scu3SYMPH GQ885268

SkleOST GQ885269

SpoCHILO GQ885270

StpARACH GQ885271

Tom2COLL GQ885272

UfsBRANCH GQ885273

3017fin:

A369COPE GQ885274

AchARACH GQ885275

AdoNEOPT GQ885276

AeliPYCNO GQ885277

AmaDIPLO GQ885278

Arg2BIURA GQ885279

Asa3BRANCH GQ885280

Avu3MALA GQ885281

Cro2XIPHOS GQ885282

Crp2ARACH GQ885283

Ctas2CHILO GQ885284

DmaBRANCH GQ885285

DtyMYSTACO GQ885286

EafCOPE GQ885287

EfrDIPLUR GQ885288

EgigARACH GQ885289

EinEPHEM GQ885290

ElePYCNO GQ885291

ErwONYCH GQ885292

EuryPAURO GQ885293

HariARACH GQ885294

HspARACH GQ885295

IpumARACH GQ885296

IveODONAT GQ885297

JapDIPLUR GQ885298

LemMALA GQ885299

LlyODONAT GQ885300

LnigARACH GQ885301

LoxTHECOS GQ885302

MayEPHEM GQ885303

MbaARCHEO GQ885304

NeoMALA GQ885305

NmeZYGEN GQ885306

PamNEOPT GQ885307

PepONYCH GQ885308

Pge2DIPLO GQ885309

Pma2ARACH GQ885310

Pol2DIPLO GQ885311

PsaARCHEO GQ885312

PwhARACH GQ885313

ScolCHILO GQ885314

Scu3SYMPH GQ885315

SkleOST GQ885316

SpoCHILO GQ885317

StpARACH GQ885318

Tom2COLL GQ885319

UfsBRANCH GQ885320

3031fin2_3:

AarPENTA GQ885321

AhiPYCNO GQ885322

AmaDIPLO GQ885323

Amb2ARACH GQ885324

Arg2BIURA GQ885325

Asa3BRANCH GQ885326

Avu3MALA GQ885327

BbaTHECOS GQ885328

CfrTHECOS GQ885329

CliZYGEN GQ885330

Col2PYCNO GQ885331

Crp2ARACH GQ885332

Ctas2CHILO GQ885333

Din2ARACH GQ885334

DmaBRANCH GQ885335

DtyMYSTACO GQ885336

EfrDIPLUR GQ885337

EgigARACH GQ885338

ElePYCNO GQ885339

EuryPAURO GQ885340

Han2SYMPH GQ885341

HariARACH GQ885342

HspARACH GQ885343

IpumARACH GQ885344

IveODONAT GQ885345

JapDIPLUR GQ885346

LeanTHECOS GQ885347

LemMALA GQ885348

Lle2BRANCH GQ885349

LlyODONAT GQ885350

LnigARACH GQ885351

LoxTHECOS GQ885352

LynBRANCH GQ885353

MbaARCHEO GQ885354

NeoMALA GQ885355

NmeZYGEN GQ885356

OimCOLL GQ885357

PamNEOPT GQ885358

Pge2DIPLO GQ885359

Pma2ARACH GQ885360

PsaARCHEO GQ885361

ScolCHILO GQ885362

Scu3SYMPH GQ885363

SpoCHILO GQ885364

StpARACH GQ885365

Tom2COLL GQ885366

UfsBRANCH GQ885367

3031fin4_5:

AarPENTA GQ885368

AchARACH GQ885369

AdoNEOPT GQ885370

AeliPYCNO GQ885371

AhiPYCNO GQ885372

AmaDIPLO GQ885373

Amb2ARACH GQ885374

Asa3BRANCH GQ885375

Avu3MALA GQ885376

BbaTHECOS GQ885377

CfrTHECOS GQ885378

CliZYGEN GQ885379

Col2PYCNO GQ885380

Crp2ARACH GQ885381

Ctas2CHILO GQ885382

Din2ARACH GQ885383

DmaBRANCH GQ885384

DtyMYSTACO GQ885385

EafCOPE GQ885386

EfrDIPLUR GQ885387

EgigARACH GQ885388

ElePYCNO GQ885389

ErwONYCH GQ885390

EuryPAURO GQ885391

Han2SYMPH GQ885392

HapaOST GQ885393

HariARACH GQ885394

HspARACH GQ885395

IpumARACH GQ885396

IveODONAT GQ885397

JapDIPLUR GQ885398

LeanTHECOS GQ885399

Lle2BRANCH GQ885400

LlyODONAT GQ885401

LnigARACH GQ885402

LoxTHECOS GQ885403

LynBRANCH GQ885404

MbaARCHEO GQ885405

NeoMALA GQ885406

OimCOLL GQ885407

PamNEOPT GQ885408

PepONYCH GQ885409

Pma2ARACH GQ885410

Pno2ONYCH GQ885411

Pol2DIPLO GQ885412

PsaARCHEO GQ885413

ScolCHILO GQ885414

Scu3SYMPH GQ885415

SpoCHILO GQ885416

StpARACH GQ885417

Tom2COLL GQ885418

3044fin:

AarPENTA GQ885419

AchARACH GQ885420

AdoNEOPT GQ885421

AhiPYCNO GQ885422

AmaDIPLO GQ885423

Amb2ARACH GQ885424

Arg2BIURA GQ885425

Asa3BRANCH GQ885426

Avu3MALA GQ885427

CfrTHECOS GQ885428

CliZYGEN GQ885429

Col2PYCNO GQ885430

Cro2XIPHOS GQ885431

Crp2ARACH GQ885432

Ctas2CHILO GQ885433

Din2ARACH GQ885434

DmaBRANCH GQ885435

EafCOPE GQ885436

EfrDIPLUR GQ885437

EinEPHEM GQ885438

ElePYCNO GQ885439

ErwONYCH GQ885440

Han2SYMPH GQ885441

HmaCEPHAL GQ885442

IpumARACH GQ885443

IveODONAT GQ885444

JapDIPLUR GQ885445

LemMALA GQ885446

Lle2BRANCH GQ885447

LlyODONAT GQ885448

LnigARACH GQ885449

LoxTHECOS GQ885450

LynBRANCH GQ885451

MayEPHEM GQ885452

MbaARCHEO GQ885453

MtdTARD GQ885454

NeoMALA GQ885455

NmeZYGEN GQ885456

OimCOLL GQ885457

PepONYCH GQ885458

Pge2DIPLO GQ885459

Pno2ONYCH GQ885460

Pol2DIPLO GQ885461

PsaARCHEO GQ885462

PwhARACH GQ885463

ScolCHILO GQ885464

Scu3SYMPH GQ885465

SkleOST GQ885466

SpoCHILO GQ885467

StpARACH GQ885468

Tom2COLL GQ885469

UfsBRANCH GQ885470

3055fin:

A369COPE GQ885471

AchARACH GQ885472

AdoNEOPT GQ885473

AeliPYCNO GQ885474

AhiPYCNO GQ885475

AmaDIPLO GQ885476

Amb2ARACH GQ885477

Arg2BIURA GQ885478

Avu3MALA GQ885479

BbaTHECOS GQ885480

CfrTHECOS GQ885481

CliZYGEN GQ885482

Col2PYCNO GQ885483

Cro2XIPHOS GQ885484

Crp2ARACH GQ885485

Ctas2CHILO GQ885486

Din2ARACH GQ885487

DmaBRANCH GQ885488

EafCOPE GQ885489

EfrDIPLUR GQ885490

EinEPHEM GQ885491

ElePYCNO GQ885492

ErwONYCH GQ885493

EuryPAURO GQ885494

Han2SYMPH GQ885495

HariARACH GQ885496

HmaCEPHAL GQ885497

HspARACH GQ885498

IveODONAT GQ885499

JapDIPLUR GQ885500

LeanTHECOS GQ885501

LemMALA GQ885502

LnigARACH GQ885503

LynBRANCH GQ885504

MayEPHEM GQ885505

MtdTARD GQ885506

NeoMALA GQ885507

NmeZYGEN GQ885508

OimCOLL GQ885509

PamNEOPT GQ885510

PepONYCH GQ885511

Pma2ARACH GQ885512

Pno2ONYCH GQ885513

PsaARCHEO GQ885514

PwhARACH GQ885515

ScolCHILO GQ885516

Scu3SYMPH GQ885517

SkleOST GQ885518

StpARACH GQ885519

Tom2COLL GQ885520

3059fin:

A369COPE GQ885521

AarPENTA GQ885522

AchARACH GQ885523

AdoNEOPT GQ885524

AeliPYCNO GQ885525

AhiPYCNO GQ885526

AmaDIPLO GQ885527

Amb2ARACH GQ885528

Arg2BIURA GQ885529

Asa3BRANCH GQ885530

Avu3MALA GQ885531

BbaTHECOS GQ885532

CfrTHECOS GQ885533

CliZYGEN GQ885534

Col2PYCNO GQ885535

Crp2ARACH GQ885536

Ctas2CHILO GQ885537

Din2ARACH GQ885538

DmaBRANCH GQ885539

DtyMYSTACO GQ885540

EafCOPE GQ885541

EfrDIPLUR GQ885542

EgigARACH GQ885543

EinEPHEM GQ885544

ErwONYCH GQ885545

EuryPAURO GQ885546

HariARACH GQ885547

HmaCEPHAL GQ885548

HspARACH GQ885549

IpumARACH GQ885550

IveODONAT GQ885551

JapDIPLUR GQ885552

LeanTHECOS GQ885553

LemMALA GQ885554

Lle2BRANCH GQ885555

LlyODONAT GQ885556

LnigARACH GQ885557

MtdTARD GQ885558

NeoMALA GQ885559

NmeZYGEN GQ885560

OimCOLL GQ885561

PamNEOPT GQ885562

PepONYCH GQ885563

Pma2ARACH GQ885564

PsaARCHEO GQ885565

PwhARACH GQ885566

ScolCHILO GQ885567

SkleOST GQ885568

SpoCHILO GQ885569

StpARACH GQ885570

Tom2COLL GQ885571

UfsBRANCH GQ885572

3064fin:

A369COPE GQ885573

AarPENTA GQ885574

AchARACH GQ885575

AdoNEOPT GQ885576

AeliPYCNO GQ885577

AhiPYCNO GQ885578

AmaDIPLO GQ885579

Amb2ARACH GQ885580

Arg2BIURA GQ885581

Asa3BRANCH GQ885582

Avu3MALA GQ885583

BbaTHECOS GQ885584

CfrTHECOS GQ885585

CliZYGEN GQ885586

Col2PYCNO GQ885587

Crp2ARACH GQ885588

Ctas2CHILO GQ885589

Din2ARACH GQ885590

DmaBRANCH GQ885591

EafCOPE GQ885592

EfrDIPLUR GQ885593

EgigARACH GQ885594

EinEPHEM GQ885595

ElePYCNO GQ885596

ErwONYCH GQ885597

EuryPAURO GQ885598

Han2SYMPH GQ885599

HapaOST GQ885600

HariARACH GQ885601

HmaCEPHAL GQ885602

HspARACH GQ885603

IpumARACH GQ885604

IveODONAT GQ885605

JapDIPLUR GQ885606

LeanTHECOS GQ885607

Lle2BRANCH GQ885608

LlyODONAT GQ885609

LnigARACH GQ885610

LoxTHECOS GQ885611

LynBRANCH GQ885612

MayEPHEM GQ885613

MtdTARD GQ885614

NeoMALA GQ885615

NmeZYGEN GQ885616

OimCOLL GQ885617

PamNEOPT GQ885618

PepONYCH GQ885619

Pge2DIPLO GQ885620

Pma2ARACH GQ885621

Pno2ONYCH GQ885622

Pol2DIPLO GQ885623

PsaARCHEO GQ885624

PwhARACH GQ885625

ScolCHILO GQ885626

Scu3SYMPH GQ885627

SkleOST GQ885628

SpoCHILO GQ885629

StpARACH GQ885630

Tom2COLL GQ885631

UfsBRANCH GQ885632

3066fin:

A369COPE GQ885633

AarPENTA GQ885634

AchARACH GQ885635

AdoNEOPT GQ885636

AmaDIPLO GQ885637

Amb2ARACH GQ885638

Arg2BIURA GQ885639

Avu3MALA GQ885640

CliZYGEN GQ885641

Col2PYCNO GQ885642

Cro2XIPHOS GQ885643

Crp2ARACH GQ885644

Ctas2CHILO GQ885645

EafCOPE GQ885646

EfrDIPLUR GQ885647

EgigARACH GQ885648

EinEPHEM GQ885649

ErwONYCH GQ885650

HariARACH GQ885651

HspARACH GQ885652

IpumARACH GQ885653

IveODONAT GQ885654

LemMALA GQ885655

Lle2BRANCH GQ885656

LlyODONAT GQ885657

LnigARACH GQ885658

LynBRANCH GQ885659

MayEPHEM GQ885660

MtdTARD GQ885661

NeoMALA GQ885662

NmeZYGEN GQ885663

OimCOLL GQ885664

PamNEOPT GQ885665

PepONYCH GQ885666

Pge2DIPLO GQ885667

Pma2ARACH GQ885668

Pol2DIPLO GQ885669

PsaARCHEO GQ885670

PwhARACH GQ885671

ScolCHILO GQ885672

SkleOST GQ885673

SpoCHILO GQ885674

StpARACH GQ885675

Tom2COLL GQ885676

3070fin:

A369COPE GQ885677

AarPENTA GQ885678

AchARACH GQ885679

AdoNEOPT GQ885680

AmaDIPLO GQ885681

Arg2BIURA GQ885682

Asa3BRANCH GQ885683

Avu3MALA GQ885684

CfrTHECOS GQ885685

CliZYGEN GQ885686

Cro2XIPHOS GQ885687

Crp2ARACH GQ885688

Din2ARACH GQ885689

DmaBRANCH GQ885690

EafCOPE GQ885691

EgigARACH GQ885692

ErwONYCH GQ885693

EuryPAURO GQ885694

Han2SYMPH GQ885695

HariARACH GQ885696

HspARACH GQ885697

IpumARACH GQ885698

IveODONAT GQ885699

LemMALA GQ885700

Lle2BRANCH GQ885701

LlyODONAT GQ885702

LnigARACH GQ885703

LoxTHECOS GQ885704

LynBRANCH GQ885705

MayEPHEM GQ885706

MtdTARD GQ885707

NeoMALA GQ885708

OimCOLL GQ885709

PamNEOPT GQ885710

PepONYCH GQ885711

Pge2DIPLO GQ885712

Pma2ARACH GQ885713

PsaARCHEO GQ885714

PwhARACH GQ885715

ScolCHILO GQ885716

Scu3SYMPH GQ885717

SkleOST GQ885718

SpoCHILO GQ885719

StpARACH GQ885720

Tom2COLL GQ885721

UfsBRANCH GQ885722

3089fin:

A369COPE GQ885723

AarPENTA GQ885724

AchARACH GQ885725

AdoNEOPT GQ885726

AeliPYCNO GQ885727

AhiPYCNO GQ885728

AmaDIPLO GQ885729

Amb2ARACH GQ885730

Arg2BIURA GQ885731

Asa3BRANCH GQ885732

Avu3MALA GQ885733

BbaTHECOS GQ885734

CfrTHECOS GQ885735

CliZYGEN GQ885736

Col2PYCNO GQ885737

Cro2XIPHOS GQ885738

Crp2ARACH GQ885739

Ctas2CHILO GQ885740

Din2ARACH GQ885741

DtyMYSTACO GQ885742

EafCOPE GQ885743

EfrDIPLUR GQ885744

EgigARACH GQ885745

EinEPHEM GQ885746

ElePYCNO GQ885747

ErwONYCH GQ885748

EuryPAURO GQ885749

Han2SYMPH GQ885750

HapaOST GQ885751

HariARACH GQ885752

HmaCEPHAL GQ885753

HspARACH GQ885754

IpumARACH GQ885755

IveODONAT GQ885756

JapDIPLUR GQ885757

LeanTHECOS GQ885758

LemMALA GQ885759

Lle2BRANCH GQ885760

LlyODONAT GQ885761

LnigARACH GQ885762

LynBRANCH GQ885763

MayEPHEM GQ885764

MtdTARD GQ885765

NeoMALA GQ885766

NmeZYGEN GQ885767

OimCOLL GQ885768

PepONYCH GQ885769

Pge2DIPLO GQ885770

Pma2ARACH GQ885771

Pno2ONYCH GQ885772

PsaARCHEO GQ885773

PwhARACH GQ885774

ScolCHILO GQ885775

Scu3SYMPH GQ885776

SkleOST GQ885777

SpoCHILO GQ885778

StpARACH GQ885779

Tom2COLL GQ885780

UfsBRANCH GQ885781

3094fin:

AchARACH GQ885782

AdoNEOPT GQ885783

AeliPYCNO GQ885784

AhiPYCNO GQ885785

AmaDIPLO GQ885786

Amb2ARACH GQ885787

Arg2BIURA GQ885788

Asa3BRANCH GQ885789

BbaTHECOS GQ885790

CfrTHECOS GQ885791

CliZYGEN GQ885792

Col2PYCNO GQ885793

Cro2XIPHOS GQ885794

Crp2ARACH GQ885795

Ctas2CHILO GQ885796

Din2ARACH GQ885797

DmaBRANCH GQ885798

DtyMYSTACO GQ885799

EafCOPE GQ885800

EfrDIPLUR GQ885801

EgigARACH GQ885802

ElePYCNO GQ885803

ErwONYCH GQ885804

EuryPAURO GQ885805

Han2SYMPH GQ885806

HapaOST GQ885807

HariARACH GQ885808

HmaCEPHAL GQ885809

HspARACH GQ885810

IpumARACH GQ885811

IveODONAT GQ885812

JapDIPLUR GQ885813

LeanTHECOS GQ885814

LemMALA GQ885815

Lle2BRANCH GQ885816

LlyODONAT GQ885817

LnigARACH GQ885818

LoxTHECOS GQ885819

LynBRANCH GQ885820

MtdTARD GQ885821

NeoMALA GQ885822

NmeZYGEN GQ885823

OimCOLL GQ885824

PamNEOPT GQ885825

PepONYCH GQ885826

Pge2DIPLO GQ885827

Pma2ARACH GQ885828

Pol2DIPLO GQ885829

PsaARCHEO GQ885830

PwhARACH GQ885831

ScolCHILO GQ885832

Scu3SYMPH GQ885833

SkleOST GQ885834

SpoCHILO GQ885835

StpARACH GQ885836

UfsBRANCH GQ885837

3114fin:

A369COPE GQ885838

AdoNEOPT GQ885839

AeliPYCNO GQ885840

AhiPYCNO GQ885841

Arg2BIURA GQ885842

Asa3BRANCH GQ885843

Avu3MALA GQ885844

BbaTHECOS GQ885845

CfrTHECOS GQ885846

CliZYGEN GQ885847

Crp2ARACH GQ885848

Ctas2CHILO GQ885849

Din2ARACH GQ885850

EafCOPE GQ885851

EfrDIPLUR GQ885852

EgigARACH GQ885853

EinEPHEM GQ885854

ElePYCNO GQ885855

ErwONYCH GQ885856

EuryPAURO GQ885857

Han2SYMPH GQ885858

IpumARACH GQ885859

IveODONAT GQ885860

JapDIPLUR GQ885861

LeanTHECOS GQ885862

LemMALA GQ885863

Lle2BRANCH GQ885864

LlyODONAT GQ885865

LnigARACH GQ885866

LoxTHECOS GQ885867

MayEPHEM GQ885868

NeoMALA GQ885869

NmeZYGEN GQ885870

PamNEOPT GQ885871

Pge2DIPLO GQ885872

Pno2ONYCH GQ885873

PsaARCHEO GQ885874

PwhARACH GQ885875

ScolCHILO GQ885876

Scu3SYMPH GQ885877

SpoCHILO GQ885878

Tom2COLL GQ885879

UfsBRANCH GQ885880

3121fin:

A369COPE GQ885881

AarPENTA GQ885882

AchARACH GQ885883

AdoNEOPT GQ885884

AeliPYCNO GQ885885

AhiPYCNO GQ885886

Amb2ARACH GQ885887

Arg2BIURA GQ885888

Asa3BRANCH GQ885889

Avu3MALA GQ885890

CliZYGEN GQ885891

Crp2ARACH GQ885892

Ctas2CHILO GQ885893

Din2ARACH GQ885894

DmaBRANCH GQ885895

EfrDIPLUR GQ885896

EgigARACH GQ885897

EinEPHEM GQ885898

ElePYCNO GQ885899

ErwONYCH GQ885900

EuryPAURO GQ885901

Han2SYMPH GQ885902

HariARACH GQ885903

HmaCEPHAL GQ885904

IpumARACH GQ885905

IveODONAT GQ885906

JapDIPLUR GQ885907

LemMALA GQ885908

Lle2BRANCH GQ885909

LlyODONAT GQ885910

LnigARACH GQ885911

LynBRANCH GQ885912

MayEPHEM GQ885913

MtdTARD GQ885914

NeoMALA GQ885915

PamNEOPT GQ885916

PepONYCH GQ885917

Pge2DIPLO GQ885918

Pma2ARACH GQ885919

Pno2ONYCH GQ885920

PsaARCHEO GQ885921

PwhARACH GQ885922

ScolCHILO GQ885923

Scu3SYMPH GQ885924

SkleOST GQ885925

SpoCHILO GQ885926

StpARACH GQ885927

UfsBRANCH GQ885928

3136fin:

A369COPE GQ885929

AarPENTA GQ885930

AchARACH GQ885931

AdoNEOPT GQ885932

AeliPYCNO GQ885933

AhiPYCNO GQ885934

AmaDIPLO GQ885935

Amb2ARACH GQ885936

Arg2BIURA GQ885937

Asa3BRANCH GQ885938

Avu3MALA GQ885939

BbaTHECOS GQ885940

CfrTHECOS GQ885941

CliZYGEN GQ885942

Col2PYCNO GQ885943

Cro2XIPHOS GQ885944

Crp2ARACH GQ885945

Ctas2CHILO GQ885946

Din2ARACH GQ885947

DmaBRANCH GQ885948

EafCOPE GQ885949

EfrDIPLUR GQ885950

EgigARACH GQ885951

EinEPHEM GQ885952

ElePYCNO GQ885953

ErwONYCH GQ885954

EuryPAURO GQ885955

Han2SYMPH GQ885956

HariARACH GQ885957

HmaCEPHAL GQ885958

HspARACH GQ885959

IpumARACH GQ885960

IveODONAT GQ885961

JapDIPLUR GQ885962

LeanTHECOS GQ885963

LemMALA GQ885964

Lle2BRANCH GQ885965

LlyODONAT GQ885966

LnigARACH GQ885967

LoxTHECOS GQ885968

LynBRANCH GQ885969

MayEPHEM GQ885970

MbaARCHEO GQ885971

MtdTARD GQ885972

NeoMALA GQ885973

NmeZYGEN GQ885974

OimCOLL GQ885975

PamNEOPT GQ885976

PepONYCH GQ885977

Pge2DIPLO GQ885978

Pma2ARACH GQ885979

Pol2DIPLO GQ885980

PsaARCHEO GQ885981

PwhARACH GQ885982

ScolCHILO GQ885983

SkleOST GQ885984

SpoCHILO GQ885985

StpARACH GQ885986

Tom2COLL GQ885987

UfsBRANCH GQ885988

3152fin:

A369COPE GQ885989

AarPENTA GQ885990

AchARACH GQ885991

AdoNEOPT GQ885992

AmaDIPLO GQ885993

Amb2ARACH GQ885994

Arg2BIURA GQ885995

Asa3BRANCH GQ885996

Avu3MALA GQ885997

BbaTHECOS GQ885998

Col2PYCNO GQ885999

Cro2XIPHOS GQ886000

Crp2ARACH GQ886001

Ctas2CHILO GQ886002

Din2ARACH GQ886003

DmaBRANCH GQ886004

EafCOPE GQ886005

EfrDIPLUR GQ886006

EgigARACH GQ886007

EinEPHEM GQ886008

ErwONYCH GQ886009

Han2SYMPH GQ886010

HariARACH GQ886011

HmaCEPHAL GQ886012

HspARACH GQ886013

IpumARACH GQ886014

IveODONAT GQ886015

JapDIPLUR GQ886016

LeanTHECOS GQ886017

Lle2BRANCH GQ886018

LlyODONAT GQ886019

LnigARACH GQ886020

LynBRANCH GQ886021

MayEPHEM GQ886022

MtdTARD GQ886023

NeoMALA GQ886024

NmeZYGEN GQ886025

OimCOLL GQ886026

PamNEOPT GQ886027

PepONYCH GQ886028

Pge2DIPLO GQ886029

Pma2ARACH GQ886030

Pno2ONYCH GQ886031

Pol2DIPLO GQ886032

PsaARCHEO GQ886033

ScolCHILO GQ886034

Scu3SYMPH GQ886035

SkleOST GQ886036

SpoCHILO GQ886037

Tom2COLL GQ886038

UfsBRANCH GQ886039

3153fin:

A369COPE GQ886040

AarPENTA GQ886041

AchARACH GQ886042

AdoNEOPT GQ886043

AeliPYCNO GQ886044

AhiPYCNO GQ886045

AmaDIPLO GQ886046

Amb2ARACH GQ886047

Arg2BIURA GQ886048

Asa3BRANCH GQ886049

Avu3MALA GQ886050

BbaTHECOS GQ886051

CfrTHECOS GQ886052

CliZYGEN GQ886053

Col2PYCNO GQ886054

Cro2XIPHOS GQ886055

Ctas2CHILO GQ886056

Din2ARACH GQ886057

DmaBRANCH GQ886058

EafCOPE GQ886059

EgigARACH GQ886060

EinEPHEM GQ886061

ElePYCNO GQ886062

ErwONYCH GQ886063

EuryPAURO GQ886064

Han2SYMPH GQ886065

HapaOST GQ886066

HariARACH GQ886067

HspARACH GQ886068

IpumARACH GQ886069

IveODONAT GQ886070

LeanTHECOS GQ886071

LemMALA GQ886072

Lle2BRANCH GQ886073

LlyODONAT GQ886074

LoxTHECOS GQ886075

LynBRANCH GQ886076

MayEPHEM GQ886077

MbaARCHEO GQ886078

NeoMALA GQ886079

NmeZYGEN GQ886080

OimCOLL GQ886081

PamNEOPT GQ886082

PepONYCH GQ886083

Pge2DIPLO GQ886084

Pma2ARACH GQ886085

Pno2ONYCH GQ886086

Pol2DIPLO GQ886087

PsaARCHEO GQ886088

ScolCHILO GQ886089

Scu3SYMPH GQ886090

SkleOST GQ886091

SpoCHILO GQ886092

StpARACH GQ886093

Tom2COLL GQ886094

UfsBRANCH GQ886095

3196fin1_3:

A369COPE GQ886096

AarPENTA GQ886097

AchARACH GQ886098

AdoNEOPT GQ886099

AeliPYCNO GQ886100

AhiPYCNO GQ886101

AmaDIPLO GQ886102

Amb2ARACH GQ886103

Arg2BIURA GQ886104

Asa3BRANCH GQ886105

Avu3MALA GQ886106

BbaTHECOS GQ886107

CfrTHECOS GQ886108

CliZYGEN GQ886109

Col2PYCNO GQ886110

Crp2ARACH GQ886111

Ctas2CHILO GQ886112

Din2ARACH GQ886113

DmaBRANCH GQ886114

DtyMYSTACO GQ886115

EafCOPE GQ886116

EfrDIPLUR GQ886117

EinEPHEM GQ886118

ElePYCNO GQ886119

EuryPAURO GQ886120

Han2SYMPH GQ886121

HariARACH GQ886122

HmaCEPHAL GQ886123

HspARACH GQ886124

IpumARACH GQ886125

IveODONAT GQ886126

JapDIPLUR GQ886127

LemMALA GQ886128

Lle2BRANCH GQ886129

LlyODONAT GQ886130

LnigARACH GQ886131

LoxTHECOS GQ886132

LynBRANCH GQ886133

MayEPHEM GQ886134

MbaARCHEO GQ886135

MtdTARD GQ886136

NeoMALA GQ886137

NmeZYGEN GQ886138

OimCOLL GQ886139

PamNEOPT GQ886140

Pge2DIPLO GQ886141

Pma2ARACH GQ886142

Pno2ONYCH GQ886143

Pol2DIPLO GQ886144

PsaARCHEO GQ886145

PwhARACH GQ886146

ScolCHILO GQ886147

Scu3SYMPH GQ886148

SkleOST GQ886149

SpoCHILO GQ886150

StpARACH GQ886151

Tom2COLL GQ886152

UfsBRANCH GQ886153

3196fin5_6:

AarPENTA GQ886154

AchARACH GQ886155

AdoNEOPT GQ886156

AeliPYCNO GQ886157

AhiPYCNO GQ886158

AmaDIPLO GQ886159

Amb2ARACH GQ886160

Arg2BIURA GQ886161

Asa3BRANCH GQ886162

Avu3MALA GQ886163

BbaTHECOS GQ886164

CfrTHECOS GQ886165

CliZYGEN GQ886166

Col2PYCNO GQ886167

Cro2XIPHOS GQ886168

Crp2ARACH GQ886169

Ctas2CHILO GQ886170

Din2ARACH GQ886171

DmaBRANCH GQ886172

DtyMYSTACO GQ886173

EafCOPE GQ886174

EfrDIPLUR GQ886175

EgigARACH GQ886176

EinEPHEM GQ886177

ElePYCNO GQ886178

EuryPAURO GQ886179

Han2SYMPH GQ886180

HariARACH GQ886181

HmaCEPHAL GQ886182

HspARACH GQ886183

IpumARACH GQ886184

IveODONAT GQ886185

JapDIPLUR GQ886186

LeanTHECOS GQ886187

LemMALA GQ886188

Lle2BRANCH GQ886189

LlyODONAT GQ886190

LnigARACH GQ886191

LoxTHECOS GQ886192

LynBRANCH GQ886193

MayEPHEM GQ886194

MbaARCHEO GQ886195

NeoMALA GQ886196

NmeZYGEN GQ886197

OimCOLL GQ886198

PamNEOPT GQ886199

PepONYCH GQ886200

Pge2DIPLO GQ886201

Pma2ARACH GQ886202

Pno2ONYCH GQ886203

Pol2DIPLO GQ886204

PsaARCHEO GQ886205

PwhARACH GQ886206

ScolCHILO GQ886207

Scu3SYMPH GQ886208

SkleOST GQ886209

SpoCHILO GQ886210

StpARACH GQ886211

Tom2COLL GQ886212

UfsBRANCH GQ886213

3202fin:

AarPENTA GQ886214

AchARACH GQ886215

AdoNEOPT GQ886216

AeliPYCNO GQ886217

AhiPYCNO GQ886218

AmaDIPLO GQ886219

Amb2ARACH GQ886220

Arg2BIURA GQ886221

Avu3MALA GQ886222

BbaTHECOS GQ886223

CliZYGEN GQ886224

Col2PYCNO GQ886225

Cro2XIPHOS GQ886226

Crp2ARACH GQ886227

Ctas2CHILO GQ886228

DmaBRANCH GQ886229

DtyMYSTACO GQ886230

EafCOPE GQ886231

EfrDIPLUR GQ886232

EinEPHEM GQ886233

ErwONYCH GQ886234

EuryPAURO GQ886235

Han2SYMPH GQ886236

HmaCEPHAL GQ886237

IveODONAT GQ886238

JapDIPLUR GQ886239

LeanTHECOS GQ886240

LemMALA GQ886241

Lle2BRANCH GQ886242

LlyODONAT GQ886243

LnigARACH GQ886244

LoxTHECOS GQ886245

LynBRANCH GQ886246

MayEPHEM GQ886247

MbaARCHEO GQ886248

MtdTARD GQ886249

NmeZYGEN GQ886250

OimCOLL GQ886251

PamNEOPT GQ886252

PepONYCH GQ886253

Pge2DIPLO GQ886254

Pma2ARACH GQ886255

Pno2ONYCH GQ886256

Pol2DIPLO GQ886257

PsaARCHEO GQ886258

PwhARACH GQ886259

ScolCHILO GQ886260

Scu3SYMPH GQ886261

SkleOST GQ886262

SpoCHILO GQ886263

StpARACH GQ886264

Tom2COLL GQ886265

8018fin:

AchARACH GQ886266

AmaDIPLO GQ886267

Amb2ARACH GQ886268

Arg2BIURA GQ886269

Asa3BRANCH GQ886270

CfrTHECOS GQ886271

CliZYGEN GQ886272

Col2PYCNO GQ886273

Cro2XIPHOS GQ886274

Crp2ARACH GQ886275

Ctas2CHILO GQ886276

Din2ARACH GQ886277

DmaBRANCH GQ886278

EafCOPE GQ886279

EgigARACH GQ886280

EinEPHEM GQ886281

HapaOST GQ886282

HariARACH GQ886283

HmaCEPHAL GQ886284

HspARACH GQ886285

IpumARACH GQ886286

IveODONAT GQ886287

JapDIPLUR GQ886288

LeanTHECOS GQ886289

Lle2BRANCH GQ886290

LlyODONAT GQ886291

LnigARACH GQ886292

LynBRANCH GQ886293

MayEPHEM GQ886294

NmeZYGEN GQ886295

OimCOLL GQ886296

PamNEOPT GQ886297

PepONYCH GQ886298

Pma2ARACH GQ886299

Pno2ONYCH GQ886300

PsaARCHEO GQ886301

PwhARACH GQ886302

ScolCHILO GQ886303

Scu3SYMPH GQ886304

SkleOST GQ886305

SpoCHILO GQ886306

StpARACH GQ886307

Tom2COLL GQ886308

UfsBRANCH GQ886309

8028fin:

AchARACH GQ886310

AdoNEOPT GQ886311

AeliPYCNO GQ886312

AhiPYCNO GQ886313

AmaDIPLO GQ886314

Amb2ARACH GQ886315

BbaTHECOS GQ886316

CfrTHECOS GQ886317

Col2PYCNO GQ886318

Crp2ARACH GQ886319

Ctas2CHILO GQ886320

DtyMYSTACO GQ886321

EafCOPE GQ886322

ElePYCNO GQ886323

ErwONYCH GQ886324

Han2SYMPH GQ886325

HapaOST GQ886326

HariARACH GQ886327

HmaCEPHAL GQ886328

HspARACH GQ886329

IveODONAT GQ886330

JapDIPLUR GQ886331

LemMALA GQ886332

Lle2BRANCH GQ886333

LlyODONAT GQ886334

LoxTHECOS GQ886335

MtdTARD GQ886336

OimCOLL GQ886337

PepONYCH GQ886338

Pge2DIPLO GQ886339

Pma2ARACH GQ886340

Pno2ONYCH GQ886341

PsaARCHEO GQ886342

PwhARACH GQ886343

ScolCHILO GQ886344

Scu3SYMPH GQ886345

SkleOST GQ886346

SpoCHILO GQ886347

StpARACH GQ886348

8029fin:

A369COPE GQ886349

AarPENTA GQ886350

AchARACH GQ886351

AdoNEOPT GQ886352

AeliPYCNO GQ886353

AhiPYCNO GQ886354

AmaDIPLO GQ886355

Amb2ARACH GQ886356

Arg2BIURA GQ886357

Asa3BRANCH GQ886358

Avu3MALA GQ886359

BbaTHECOS GQ886360

CliZYGEN GQ886361

Col2PYCNO GQ886362

Crp2ARACH GQ886363

Ctas2CHILO GQ886364

Din2ARACH GQ886365

DmaBRANCH GQ886366

DtyMYSTACO GQ886367

EafCOPE GQ886368

EfrDIPLUR GQ886369

EgigARACH GQ886370

EinEPHEM GQ886371

ElePYCNO GQ886372

ErwONYCH GQ886373

EuryPAURO GQ886374

Han2SYMPH GQ886375

HapaOST GQ886376

HariARACH GQ886377

HmaCEPHAL GQ886378

HspARACH GQ886379

IpumARACH GQ886380

IveODONAT GQ886381

JapDIPLUR GQ886382

LemMALA GQ886383

Lle2BRANCH GQ886384

LlyODONAT GQ886385

LnigARACH GQ886386

LoxTHECOS GQ886387

MayEPHEM GQ886388

MbaARCHEO GQ886389

NeoMALA GQ886390

NmeZYGEN GQ886391

OimCOLL GQ886392

PamNEOPT GQ886393

PepONYCH GQ886394

Pge2DIPLO GQ886395

Pma2ARACH GQ886396

Pno2ONYCH GQ886397

Pol2DIPLO GQ886398

PsaARCHEO GQ886399

PwhARACH GQ886400

ScolCHILO GQ886401

Scu3SYMPH GQ886402

SpoCHILO GQ886403

StpARACH GQ886404

Tom2COLL GQ886405

UfsBRANCH GQ886406

8053fin:

AarPENTA GQ886407

AchARACH GQ886408

AdoNEOPT GQ886409

AeliPYCNO GQ886410

AhiPYCNO GQ886411

AmaDIPLO GQ886412

Amb2ARACH GQ886413

Asa3BRANCH GQ886414

Avu3MALA GQ886415

BbaTHECOS GQ886416

CfrTHECOS GQ886417

CliZYGEN GQ886418

Col2PYCNO GQ886419

Cro2XIPHOS GQ886420

Crp2ARACH GQ886421

Din2ARACH GQ886422

DtyMYSTACO GQ886423

EfrDIPLUR GQ886424

EgigARACH GQ886425

EinEPHEM GQ886426

ElePYCNO GQ886427

ErwONYCH GQ886428

EuryPAURO GQ886429

Han2SYMPH GQ886430

HariARACH GQ886431

HmaCEPHAL GQ886432

HspARACH GQ886433

IpumARACH GQ886434

IveODONAT GQ886435

JapDIPLUR GQ886436

LeanTHECOS GQ886437

LemMALA GQ886438

LlyODONAT GQ886439

LnigARACH GQ886440

LoxTHECOS GQ886441

LynBRANCH GQ886442

MayEPHEM GQ886443

NeoMALA GQ886444

NmeZYGEN GQ886445

OimCOLL GQ886446

PamNEOPT GQ886447

PepONYCH GQ886448

Pma2ARACH GQ886449

Pno2ONYCH GQ886450

Pol2DIPLO GQ886451

PsaARCHEO GQ886452

PwhARACH GQ886453

ScolCHILO GQ886454

Scu3SYMPH GQ886455

SkleOST GQ886456

SpoCHILO GQ886457

Tom2COLL GQ886458

UfsBRANCH GQ886459

8070fin:

A369COPE GQ886460

AarPENTA GQ886461

AchARACH GQ886462

AdoNEOPT GQ886463

AhiPYCNO GQ886464

AmaDIPLO GQ886465

Amb2ARACH GQ886466

BbaTHECOS GQ886467

CfrTHECOS GQ886468

CliZYGEN GQ886469

Cro2XIPHOS GQ886470

Crp2ARACH GQ886471

Ctas2CHILO GQ886472

Din2ARACH GQ886473

DmaBRANCH GQ886474

EafCOPE GQ886475

EfrDIPLUR GQ886476

EinEPHEM GQ886477

EuryPAURO GQ886478

HariARACH GQ886479

HmaCEPHAL GQ886480

HspARACH GQ886481

IpumARACH GQ886482

IveODONAT GQ886483

JapDIPLUR GQ886484

LeanTHECOS GQ886485

LemMALA GQ886486

Lle2BRANCH GQ886487

LlyODONAT GQ886488

LnigARACH GQ886489

LoxTHECOS GQ886490

LynBRANCH GQ886491

MayEPHEM GQ886492

MbaARCHEO GQ886493

NmeZYGEN GQ886494

PamNEOPT GQ886495

PepONYCH GQ886496

Pge2DIPLO GQ886497

Pma2ARACH GQ886498

Pol2DIPLO GQ886499

PsaARCHEO GQ886500

PwhARACH GQ886501

ScolCHILO GQ886502

Scu3SYMPH GQ886503

SpoCHILO GQ886504

8091fin:

A369COPE GQ886505

AarPENTA GQ886506

AchARACH GQ886507

AhiPYCNO GQ886508

Amb2ARACH GQ886509

Asa3BRANCH GQ886510

Avu3MALA GQ886511

Cro2XIPHOS GQ886512

Din2ARACH GQ886513

EafCOPE GQ886514

EgigARACH GQ886515

EinEPHEM GQ886516

Han2SYMPH GQ886517

HariARACH GQ886518

HspARACH GQ886519

IveODONAT GQ886520

JapDIPLUR GQ886521

LemMALA GQ886522

Lle2BRANCH GQ886523

LlyODONAT GQ886524

LnigARACH GQ886525

LoxTHECOS GQ886526

LynBRANCH GQ886527

MbaARCHEO GQ886528

NeoMALA GQ886529

NmeZYGEN GQ886530

PamNEOPT GQ886531

PepONYCH GQ886532

Pge2DIPLO GQ886533

Pno2ONYCH GQ886534

Pol2DIPLO GQ886535

PsaARCHEO GQ886536

PwhARACH GQ886537

StpARACH GQ886538

UfsBRANCH GQ886539

acc:

A369COPE GQ886540

AeliPYCNO GQ886541

AhiPYCNO GQ886542

Amb2ARACH GQ886543

Arg2BIURA GQ886544

Asa3BRANCH GQ886545

Avu3MALA GQ886546

BbaTHECOS GQ886547

CfrTHECOS GQ886548

CliZYGEN GQ886549

Col2PYCNO GQ886550

Cro2XIPHOS GQ886551

Crp2ARACH GQ886552

Ctas2CHILO GQ886553

Din2ARACH GQ886554

DmaBRANCH GQ886555

DtyMYSTACO GQ886556

EafCOPE GQ886557

EgigARACH GQ886558

ElePYCNO GQ886559

ErwONYCH GQ886560

EuryPAURO GQ886561

HariARACH GQ886562

HmaCEPHAL GQ886563

HspARACH GQ886564

IpumARACH GQ886565

LeanTHECOS GQ886566

LemMALA GQ886567

Lle2BRANCH GQ886568

MtdTARD GQ886569

NeoMALA GQ886570

OimCOLL GQ886571

PamNEOPT GQ886572

PepONYCH GQ886573

Pma2ARACH GQ886574

Pno2ONYCH GQ886575

PsaARCHEO GQ886576

PwhARACH GQ886577

ScolCHILO GQ886578

SpoCHILO GQ886579

StpARACH GQ886580

Tom2COLL GQ886581

UfsBRANCH GQ886582

aspec2_6:

AeliPYCNO GQ886583

AhiPYCNO GQ886584

Amb2ARACH GQ886585

Arg2BIURA GQ886586

Avu3MALA GQ886587

BbaTHECOS GQ886588

CfrTHECOS GQ886589

CliZYGEN GQ886590

Col2PYCNO GQ886591

Cro2XIPHOS GQ886592

Crp2ARACH GQ886593

Ctas2CHILO GQ886594

DmaBRANCH GQ886595

EgigARACH GQ886596

ElePYCNO GQ886597

ErwONYCH GQ886598

HariARACH GQ886599

HmaCEPHAL GQ886600

HspARACH GQ886601

JapDIPLUR GQ886602

LeanTHECOS GQ886603

LemMALA GQ886604

MayEPHEM GQ886605

NeoMALA GQ886606

OimCOLL GQ886607

PamNEOPT GQ886608

PsaARCHEO GQ886609

ScolCHILO GQ886610

SpoCHILO GQ886611

Tom2COLL GQ886612

UfsBRANCH GQ886613

aspec11_12:

AeliPYCNO GQ886614

AhiPYCNO GQ886615

Amb2ARACH GQ886616

Arg2BIURA GQ886617

Asa3BRANCH GQ886618

Avu3MALA GQ886619

BbaTHECOS GQ886620

CfrTHECOS GQ886621

CliZYGEN GQ886622

Col2PYCNO GQ886623

Cro2XIPHOS GQ886624

Crp2ARACH GQ886625

Ctas2CHILO GQ886626

Din2ARACH GQ886627

DmaBRANCH GQ886628

DtyMYSTACO GQ886629

EafCOPE GQ886630

EgigARACH GQ886631

ElePYCNO GQ886632

HariARACH GQ886633

HmaCEPHAL GQ886634

JapDIPLUR GQ886635

LeanTHECOS GQ886636

LemMALA GQ886637

Lle2BRANCH GQ886638

MayEPHEM GQ886639

MtdTARD GQ886640

NeoMALA GQ886641

OimCOLL GQ886642

PamNEOPT GQ886643

PepONYCH GQ886644

PsaARCHEO GQ886645

PwhARACH GQ886646

ScolCHILO GQ886647

SpoCHILO GQ886648

Tom2COLL GQ886649

UfsBRANCH GQ886650

aspec19_21:

AeliPYCNO GQ886651

AhiPYCNO GQ886652

Amb2ARACH GQ886653

Arg2BIURA GQ886654

Asa3BRANCH GQ886655

Avu3MALA GQ886656

BbaTHECOS GQ886657

CfrTHECOS GQ886658

CliZYGEN GQ886659

Col2PYCNO GQ886660

Crp2ARACH GQ886661

Ctas2CHILO GQ886662

Din2ARACH GQ886663

DmaBRANCH GQ886664

DtyMYSTACO GQ886665

EafCOPE GQ886666

EgigARACH GQ886667

ElePYCNO GQ886668

ErwONYCH GQ886669

EuryPAURO GQ886670

Han2SYMPH GQ886671

HmaCEPHAL GQ886672

IpumARACH GQ886673

JapDIPLUR GQ886674

LeanTHECOS GQ886675

LemMALA GQ886676

Lle2BRANCH GQ886677

MayEPHEM GQ886678

MtdTARD GQ886679

NeoMALA GQ886680

OimCOLL GQ886681

PamNEOPT GQ886682

PepONYCH GQ886683

Pno2ONYCH GQ886684

PsaARCHEO GQ886685

PwhARACH GQ886686

ScolCHILO GQ886687

SpoCHILO GQ886688

Tom2COLL GQ886689

UfsBRANCH GQ886690

ef1a:

AarPENTA GQ886691

AdoNEOPT GQ886692

AeliPYCNO GQ886693

AhiPYCNO GQ886694

DmaBRANCH GQ886695

DtyMYSTACO GQ886696

EgigARACH GQ886697

EinEPHEM GQ886698

ErwONYCH GQ886699

IpumARACH GQ886700

IveODONAT GQ886701

LlyODONAT GQ886702

LnigARACH GQ886703

PepONYCH GQ886704

Pno2ONYCH GQ886705

PwhARACH GQ886706

ef2:

AarPENTA GQ886707

AchARACH GQ886708

AdoNEOPT GQ886709

AeliPYCNO GQ886710

AhiPYCNO GQ886711

Amb2ARACH GQ886712

Crp2ARACH GQ886713

Din2ARACH GQ886714

DmaBRANCH GQ886715

DtyMYSTACO GQ886716

EgigARACH GQ886717

EinEPHEM GQ886718

ErwONYCH GQ886719

HariARACH GQ886720

HspARACH GQ886721

IpumARACH GQ886722

LlyODONAT GQ886723

LnigARACH GQ886724

PepONYCH GQ886725

Pma2ARACH GQ886726

PwhARACH GQ886727

StpARACH GQ886728

polii:

AarPENTA GQ886729

AchARACH GQ886730

AdoNEOPT GQ886731

AeliPYCNO GQ886732

AhiPYCNO GQ886733

Amb2ARACH GQ886734

Crp2ARACH GQ886735

Ctas2CHILO GQ886736

Din2ARACH GQ886737

DmaBRANCH GQ886738

EgigARACH GQ886739

EinEPHEM GQ886740

ErwONYCH GQ886741

IpumARACH GQ886742

IveODONAT GQ886743

LlyODONAT GQ886744

LnigARACH GQ886745

PepONYCH GQ886746

Pma2ARACH GQ886747

Pno2ONYCH GQ886748

PwhARACH GQ886749

StpARACH GQ886750

**Previously cited GenBank accession numbers for all genes except EF-1alpha, EF-2 and PolII can be found in:**

Ref. 16 (Regier, J.C., Shultz, J.W., Ganley, A.R.D., Hussey, A., Shi, D., Ball, B. Stajich, J.E., Cummings, M.P., Martin, J.W., and Cunningham, C.W. 2008. Resolving Arthropod Phylogeny: Exploring Phylogenetic Signal within 41kb of Protein-coding Nuclear Gene Sequence. Syst. Biol. 57: 920-938.)

**Previously cited GenBank accession numbers for EF-1alpha, EF-2 and PolII can be found in:**

Regier, J.C., J.W. Shultz, and R.E. Kambic. 2005. Pancrustacean phylogeny: Hexapods are terrestrial crustaceans and maxillopods are not monophyletic. Proc. R. Soc. Lond. B 272:305-401.

Regier, J.C., H.M. Wilson, and J.W. Shultz. 2005. Phylogenetic analysis of Myriapoda using three nuclear protein-coding genes. Mol. Phylog. Evol. 34:147-158.

^a^ Taken from [11].
